# Photocatalytic Reductive Desulfonation of Aryl Tosylates

**DOI:** 10.1021/acs.joc.5c02061

**Published:** 2025-11-18

**Authors:** Kasmier Vicioso, Jonathan Santoro, Zachary Coren, Rashanique D. Quarels

**Affiliations:** 1 Department of Chemistry and Biochemistry, 3536 Rowan University, Glassboro, New Jersey 08028, United States; 2 Department of Biological and Biomedical Sciences, 3536 Rowan University, Glassboro, New Jersey 08028, United States

## Abstract

This communication describes the reductive deprotection of aryl tosylates to their corresponding phenols in up to 93% yield using visible-light photocatalysis. In this new methodology, the photoexcited enolate generated from the deprotonation of 10-bromoanthracen-9-one and subsequent excitation is capable of selectively cleaving the S–O bond on the aryl tosylate substrate, releasing the phenolate anion. Moreover, this deprotection method does not perturb common functional groups. Thus, this transformation affords an orthogonal deprotection strategy and has potential for use in the late-stage deprotection of aryl tosylates in organic synthesis and medicinal chemistry. The design, scope, and limitations are discussed.

## Graphical abstract

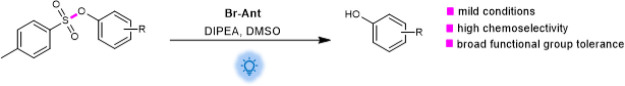

## Introduction

The *para*-toluenesulfonyl (tosyl) group is a common protecting group for alcohols, phenols, and amines in multistep organic synthesis.^1^ Tosylates are key synthons in various reactions such as substitutions, eliminations, and cross-couplings. Toluenesulfonate esters are stable to heat, acidic conditions, and lithium aluminum hydride. Tosylates are highly versatile due to their relative stability (aryl) and excellent leaving group ability (alkyl). The deprotection of aryl toluenesulfonates is often carried out in the presence of strong bases, under dissolving metal conditions, using electrochemical methods.^1–7^ A few of the common aryl tosylate deprotection strategies are highlighted in Scheme 1. Wolfrom et al. published the first recorded tosyl deprotection report as part of their strategy to protect the phenols in the synthesis of osajaxanthone.^1b^ The authors relied on the use of potassium hydroxide, a strong base, for the *p*-toluenesulfonate cleavage.^1b^ In 2000, Nayak introduced a mild method for the deprotection of tosylate esters and *p*-methylbenzenesulfonamides using low-valent titanium reagents. Notably, the method was tolerant of methyl and chloride substitution on the aromatic ring.^1c^ Tsai et al. expanded the use of dissolving metal reduction for the deprotection of tosylates.^1d^

**Scheme 1:**
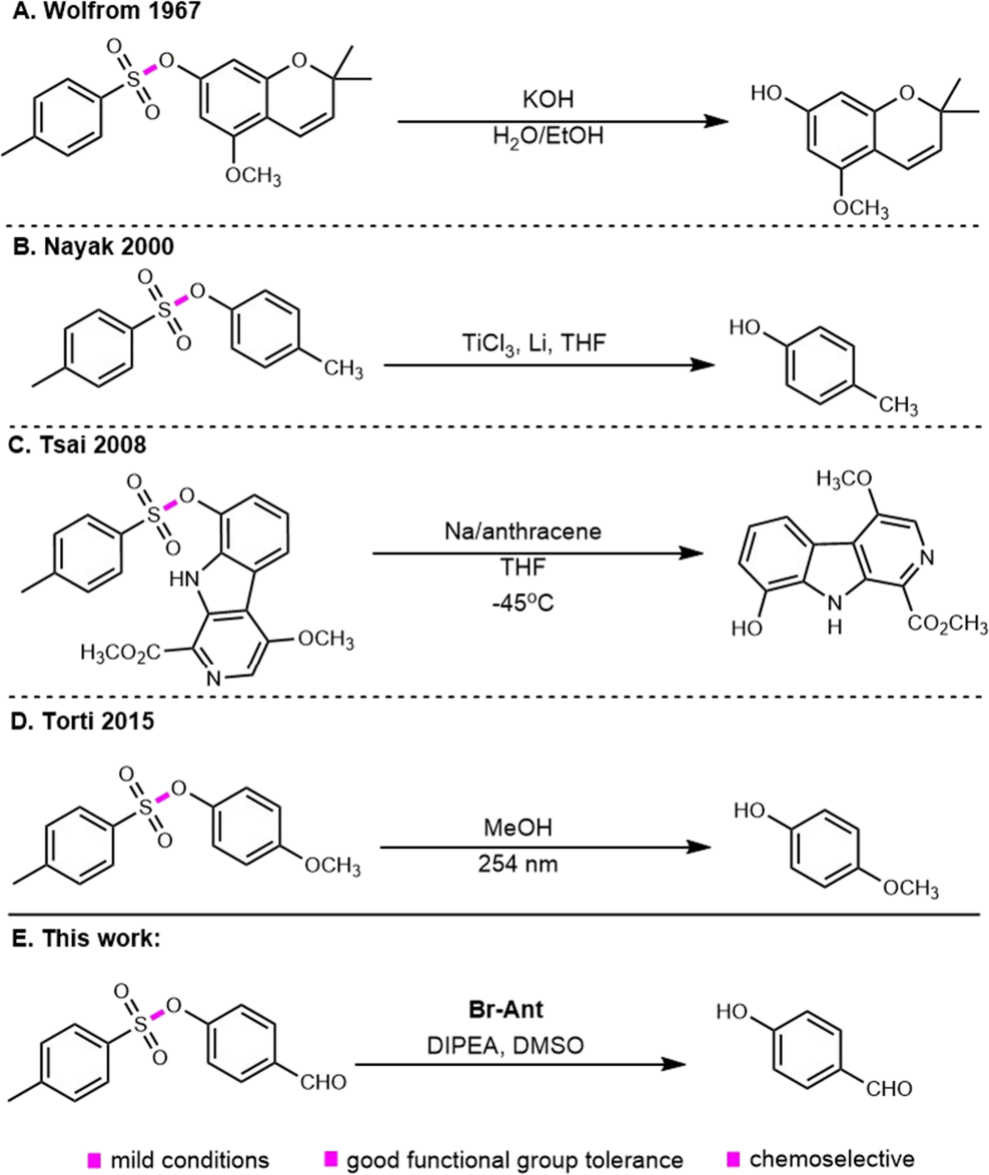
Deprotection of Aryl Tosylates

Mairanovsky’s early work and review on electrochemical removal of protecting groups revealed that tosyl cleavage (S–O) was selective and high-yielding (70–90%).^2^ Both alkyl and aryl toluenesulfonates were converted to the corresponding alcohols and phenols requiring 8–12 equiv of magnesium in methanol.^3^ Choi et al. demonstrated that aryl benzenesulfonates are capable of deprotection (S–O cleavage) and, more surprisingly, nucleophilic substitution (C–O cleavage). The product resulting from the heating was dependent on the amine nucleophile, solvent, and the substituents on the phenoxide.^4^ Similarly, Eroğlu et al. observed S–O cleavage to form phenoxides upon heating aryl tosylates with Grignard reagents.^5^ Gao and Yang^6^ as well as Iglesias et al.^7^ were able to achieve C–O cleavage utilizing nickel catalysis under thermal conditions. Of important note, each team observed lower yields for electron-rich aryl tosylates.^6,7^

In 2015, Torti and co-workers explored the photochemistry of aryl tosylates, particularly in their role as photoacid generators; see Scheme 1.^8^ Their work highlighted the direct activation of phenols. However, the use of ultraviolet irradiation necessary for photolysis was incompatible with various functional groups such as aldehydes, ketones, and nitro groups. Perez et al. demonstrated that CuInS_2_/ZnS quantum dots could be used as photocatalysts for the reductive deprotection of various phenyl aryl sulfonyl-protected phenols.^11^ Perez and co-workers tested 11 benzenesulfonates with various substitutions. The authors were able to demonstrate that the method was compatible with common functional groups, such as carbamate, benzyl, and even aryl tosylate. Notably, phenyl benzenesulfonate and phenyl *p*-toluenesulfonate were unreactive in the presence of the irradiated CuInS_2_/ZnS quantum dots.^11^ Owing to the ubiquitous use and economical pricing of the *p*-toluenesulfonyl protecting group, a mild and functional group-tolerant photocatalytic reductive deprotection strategy of aryl tosylates would advance organic synthesis.

We questioned whether aryl tosylates could be cleaved using visible-light photocatalysis. Specifically, we are interested in the use of inexpensive and minimally toxic organic photocatalysts to enable reductive deprotection. Organic synthesizers are a viable alternative for the expensive ruthenium and iridium photocatalyst complexes.^9,10^ Recent expansion of organic dyes and small molecules as photocatalysts has helped to broaden the photoredox capabilities of organic synthesizers.^10^

More recently, Schmalzbauer et al. reported that an anionic excited-state donor can transfer an electron to a neutral acceptor, leading to the formation of both a radical and a radical anion intermediate.^12^ The study done by Schmalzbauer and co-workers highlighted the effectiveness of 9-anthrone derivatives as potent reductants, successfully converting aryl chlorides into aryl radicals for synthesis of biaryl compounds.^12^ This finding is particularly noteworthy, as aryl chlorides, such as aryl tosylates, are notoriously difficult to reduce. Anionic molecules generally serve as superior electron donors compared to their neutral counterparts due to increased electron–electron repulsion, which facilitates electron transfer.^10,12^ As a result, the presence of a negative charge significantly enhances the ease of electron donation.

In 2022, Glaser and Wenger developed a dual-photoredox approach by merging an organic photocatalyst and a copper catalyst. The authors developed methods for hydrodehalogenation, as well as the desulfonation of sulfonamides and aryl sulfonates.^13^ The six aryl sulfonate examples demonstrated by Glaser and Wenger showed that their dual catalysis method was amenable to several common functional groups such as ketones, amides, halides, and methyl ethers. Additionally, Pavlovska et al. demonstrated the N–S cleavage of various sulfonamides using metal-free photocatalytic reductive desulfonylation mediated by deazaalloxazines using visible light.^14^ The authors also extended their method to *p*-tolyl trifluoromethanesulfonate and *p*-tolyl 4-methylbenzenesulfonate. Notably, the flavin photocatalyst deazaalloxazine was able to selectively cleave the S–O of both aryl sulfonates.

## Results and Discussion

The origin of this study was the application of phenols as radical precursors. Recently, photocatalysts exhibiting strong reductive redox properties have gained attention as effective reagents for facilitating reduction reactions and radical generation under mild conditions.^8–12,15^ Several of the conditions we explored resulted in the selective deprotection of the aryl tosylate at varying efficiency. Using the 10-bromoanthracen-9­(10H)-one catalyst (**Br-ANT**),^17^ irradiation^18^ of the substrate 4-formylphenyl 4-methylbenzenesulfonate (**1a**), and potassium carbonate in anhydrous DMSO resulted in the generation of 4-hydroxybenzaldehyde (**2a**) in moderate yield (Table 1, entry 1). The behavior of the substrate was benchmarked using cesium carbonate as the base (Table 1, entry 2), and the yield improved significantly. Use of amine bases (Table 1, entries 3 and 4) provided 4-hydroxybenzaldehyde in higher yields and higher conversion than previously observed in entry 1. *N,N*-diisopropylethylamine (DIPEA) was found to give complete conversion of the tosylate with an 82% isolated yield of the resulting phenol. Further exploration of the reaction revealed that trace amounts of the desulfonation product were observed without the presence of the 10-bromoanthrone catalyst (Table 1, entry 6). We postulated that under irradiation^18^ in DMSO, DIPEA was activated and served as a sacrificial reductant. However, it was observed that the reaction was not viable without light or base, as only starting material **1a** was recovered after isolation via column chromatography.

**Table 1:**
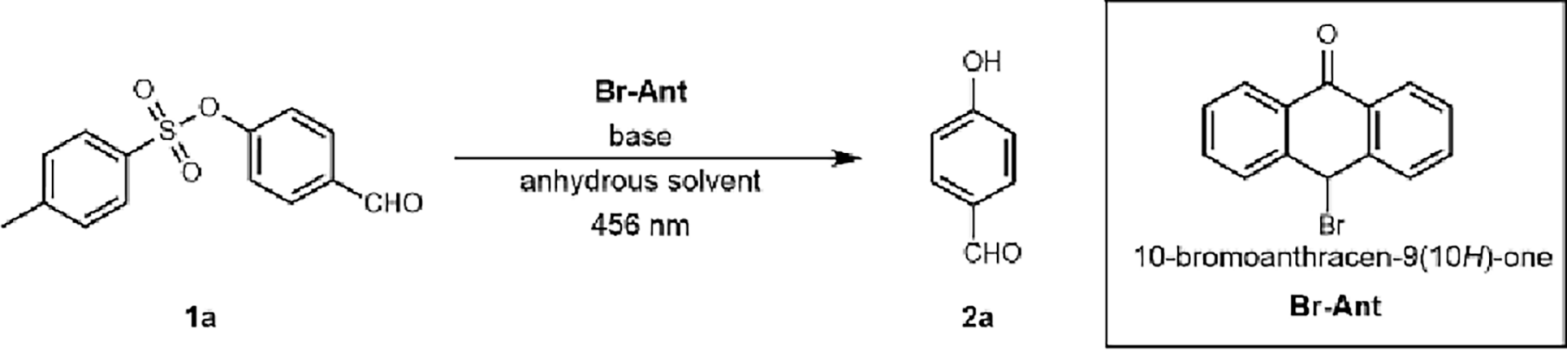
Optimization of Reaction Conditions^a^

entry	scale (mmol)	base	concentration (M)	catalyst mol %	solvent	yield^b^ (%)
1^c^	0.2	K_2_CO_3_	0.1	20	DMSO	26
2^c^	0.2	Cs_2_CO_3_	0.1	20	DMSO	71
3	0.2	DIPEA	0.1	20	DMSO	82
4	0.2	Et_3_N	0.1	20	DMSO	51
5	0.2	DIPEA	0.1	10	DMSO	47
6	0.2	DIPEA	0.1	0	DMSO	trace
7	0.2	none	0.1	20	DMSO	0
8^d^	0.2	DIPEA	0.1	20	DMSO	0

a Reaction conditions: substrate 1 (0.2 mmol) and catalyst Br-Ant (0.04 mmol) were dissolved in anhydrous DMSO (2.0 mL) in a 1-dram sealed vial equipped with a micro stir bar under nitrogen atmosphere. DIPEA (0.6 mmol) was added via syringe needle after two cycles of freeze–pump–thaw followed by one additional freeze–pump–thaw cycle. While stirring, the reaction was exposed to Blue LED lights (456 nm) for 24 h. **Caution!** 10-Bromoanthrone is light-sensitive. The spent catalyst was discarded in the organic waste as a solution in ethyl acetate upon degradation. **Caution!** Extreme care should be taken both in the handling of the cryogen liquid nitrogen and its use in the Schlenk line trap to avoid the condensation of oxygen from air. **Caution!** This procedure requires the use of specialized visible-light sources. Precaution is required when working with the Kessil lamps, and protective eyewear must be used at all times.

b Yields were determined by isolation via column chromatography.

### Substrate Scope

With the preliminary results in hand, the photocatalytic method was extended to include substrates with varying electronic structure. All photocatalytic experiments were done in triplicate, and average yields were reported. A variety of aryl tosylates bearing electron-poor aryl substituents, including cyano, nitro, ester, and ketone, were tolerated and afforded the corresponding phenols in moderate to good yields (Scheme 2). Single-aryl substitution with electron-withdrawing groups were converted to the corresponding phenols (**2a**, **2d**, and **2e** in 82, 78, and 50%, respectively). Aryl tosylates with both electron-withdrawing and electron-donating groups were also compatible (**2b** and **2c**). Electron-rich aryl substituents, in particular alkyl amine, benzyl, methoxy, and methyl groups with varying substitution patterns (*m-*, *o-*, and *p-*), were also well tolerated under the standard conditions. Aryl tosylates with a single methoxy group (*ortho*-**2l** and *meta-* **2m**) were converted to the resulting phenols in an excellent yield. Further evaluation of alkyl substituted aryl tosylates was also isolated in favorable quantities (**2o**–**2r**). The additional benzene ring of the 1-naphthol substrate did not alter the reactivity (**2s**). Notably, substrate **2f** was successfully obtained as the only product under the standard reaction conditions without damaging the reactive olefin. These results demonstrate that our methodology is chemoselective under these strongly reducing potentials.

**Scheme 2:**
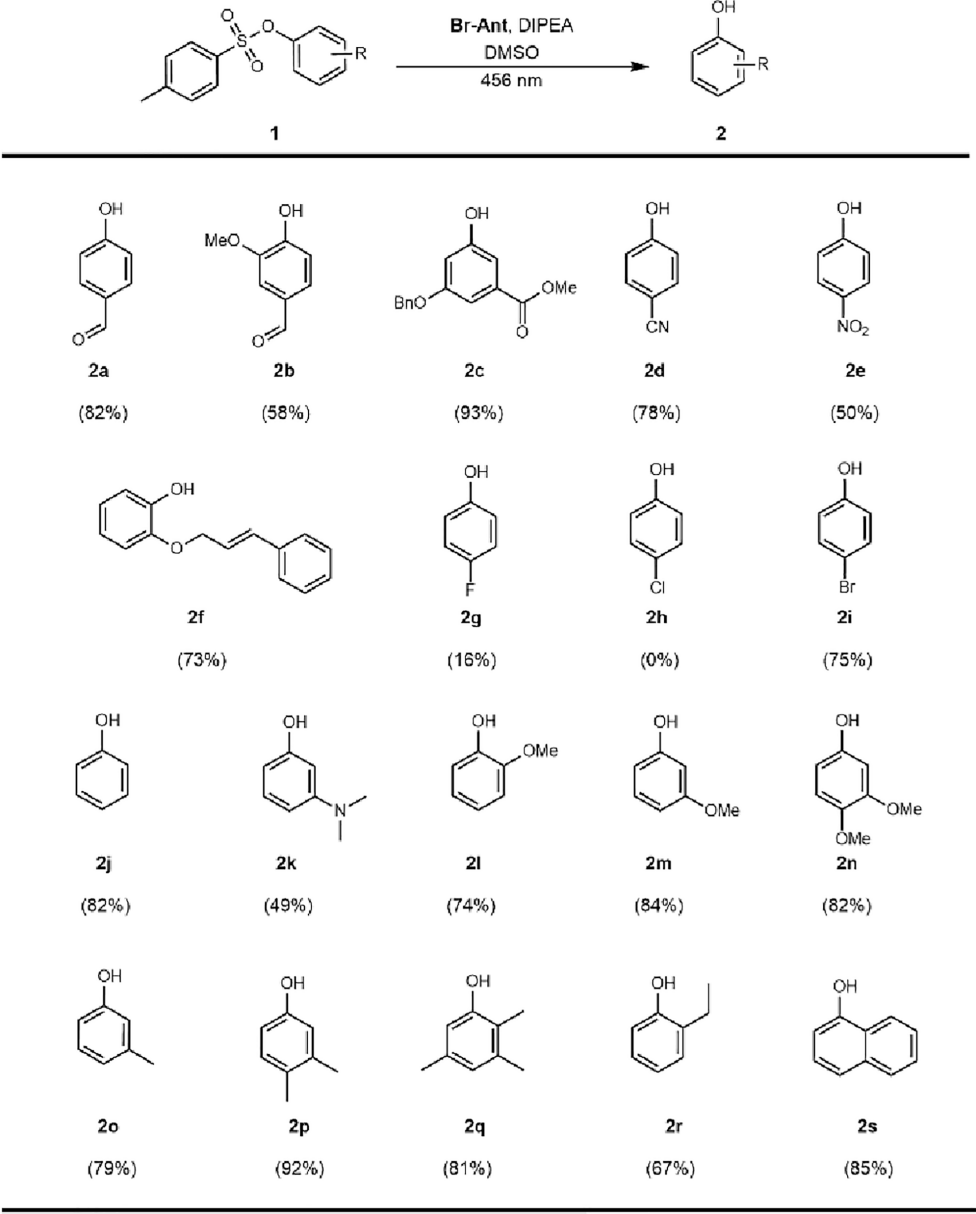
Substrate Scope of Aryl Tosylates^a^

a Reaction conditions: substrate 1 (0.2 mmol) and catalyst Br-Ant (0.04 mmol) were dissolved in anhydrous DMSO (2.0 mL) in a 1-dram sealed vial equipped with a micro stir bar under nitrogen atmosphere. DIPEA (0.6 mmol) was added via syringe needle after two cycles of freeze–pump–thaw followed by one additional freeze–pump–thaw cycle. While stirring the reaction was exposed to Blue LED lights (456 nm) for 24 h. **Caution!** 10-Bromoanthrone is light-sensitive. The spent catalyst was discarded in the organic waste as a solution in ethyl acelate upon degradation. **Caution!** Extreme care should be taken both in the handling of the cyrogen liquid nitrogen and its use in the Schlenk line trap to avoid the condensation of oxygen from air. **Caution!** This procedure requires the use of specialized visible-light sources. Precaution is required when working with the Kessil lamps, and protective eyewear must be used at all times.
b Yields were determined by isolation via column chromatography.

We also investigated whether aryl tosylates with halogenated substituents would participate in selective detosylation or result in competitive side reactions. Under standard conditions, irradiation of the aryl bromide **1i** led to isolation of 4-bromophenol **2i** as the major product in 75% yield. However, the aryl fluoride **1g** yielded significantly less of the desired 4-fluorophenol **2g** (16%) even though complete conversion of the starting tosylate **1g** was observed. The 4-chlorophenyl-4-methylbenzenesulfonate, **1h**, was also completely converted after 24 h; however, no evidence of **2h** was observed. We postulate that competitive reduction of chloride and subsequent polymerization occurred due to similar reduction potentials of the chloride (C–Cl) and tosylate (S–O) bonds.^11,17^

### Mechanistic Experiment and Proposed Mechanism

Short-lived radicals are common intermediates of photoredox catalysis.^10d^ In order to elucidate more details of the reaction mechanism, we executed a radical trapping experiment; see Scheme 3. The desulfonation of 4-formylphenyl 4-methylbenzenesulfonate **1a** was investigated under standard conditions in the presence of the radical scavenger 2,2,6,6-tetramethylpiperdine-1-oxyl (TEMPO). The tosylate **1a** was recovered in nearly quantitative yield after workup and column chromatography. The result of this study suggests that the addition of TEMPO completely shuts down the photoredox process by quenching the excited-state organocatalyst. Additionally, the detosylation method was performed on a 2.0 mmol scale, 10×, using tosylate **1a**. The resulting phenol **2a** was isolated in a 70% yield. More information regarding the TEMPO experiment and larger scale synthesis can be found in the Supporting Information.

**Scheme 3:**
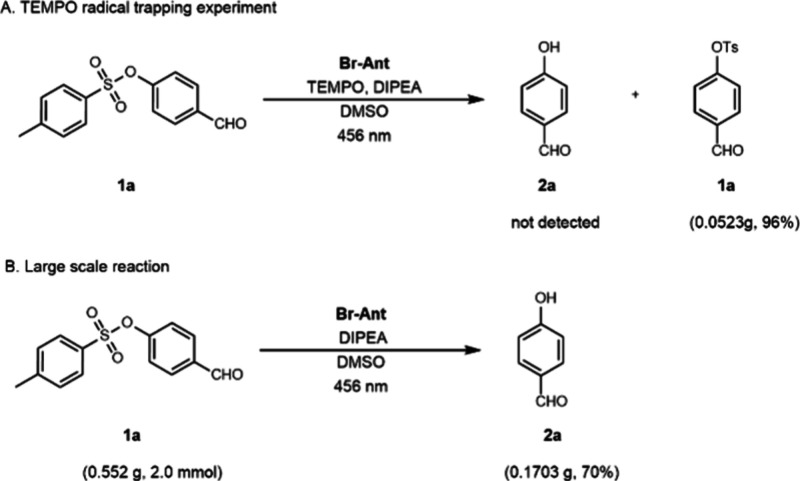
Mechanistic Investigation

Based on literature precedence and our radical trapping results, we propose that in the presence of *N,N*-diisopropylethylamine (DIPEA), **Br-ANT** is deprotonated producing the ^–^ **Br-ANT**, the enolate as shown in Scheme 4. Kerzig and Goez first investigated the photoionization of 9-anthrolate (^−^ **AOL**) in water using near UV laser light (355 nm).^16^ They determined that the first photon generates the excited anionic species,*^–^ **AOL**. The excited-state anion serves as an electron donor, yielding an anthroxy radical (·**AOL**). Regeneration of ^–^ **AOL** occurs upon receiving an electron from the sacrificial donor. Konig and co-workers^12,15^ previously observed that the enolate of 9-anthrone causes a red shift in the absorption spectrum, and we assert that a similar phenomenon occurs with **Br-ANT** shifting the enolate absorption band in the visible range. ^–^ **Br-ANT** is then excited with blue LED light leading to the formation of * ^–^ **Br-ANT**, the strongly reducing excited anion (see Scheme 2). Single-electron transfer from * ^–^ **Br-ANT** to the aryl tosylate **1** results in the short-lived radical anion **IV**. Loss of the sulfonyl group (S–O cleavage) leaves phenolate anion **V** that is later quenched upon acidic workup. Additionally, we postulate that DIPEA serves as a sacrificial electron donor necessary to turnover the catalyst.

**Scheme 4:**
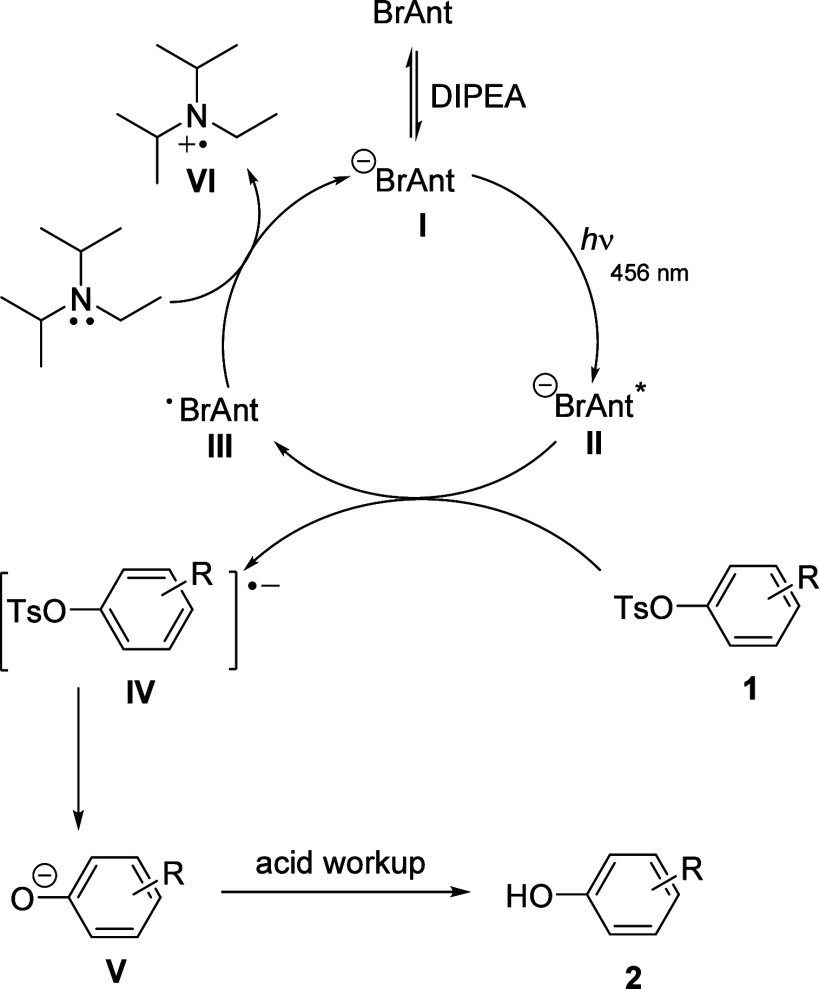
Plausible Mechanism

## Conclusions

In summary, we have developed a photoredox-catalyzed deprotection reaction of aryl tosylates resulting in a moderate to high yield of the resulting phenols. We acknowledge that halogens, especially aryl chlorides and fluorides, undergo competitive side reactions. The photoreductive tosylate deprotection method demonstrated divergence from other synthetic methods avoiding the use of strong nucleophiles or strong acids for S–O bond cleavage. Our approach offers chemoselectivity in the presence of ester and ether bonds. This strategy also tolerates various functional groups (i.e., alkene, ketone, cyano, and nitro groups) and holds promise for application in the late-stage deprotection of aryl tosylate protecting groups on small molecules as well as complex bioactive molecules.

## Supplementary Material



## Data Availability

The data underlying this study are available in the published article and its Supporting Information. For any additional information, please contact the corresponding author.

## Notes

General information, general procedures for the synthesis of sulfonates, reaction conditions, scale-up experiment, mechanism studies, characterization of sulfonates and phenol products, copies of NMR spectra (PDF)
