# Supplementary material for: Photocatalytic Reductive Desulfonation of Aryl Tosylates
Source: J Org Chem. 2025 Nov 18;90(47):16828–32. doi: 10.1021/acs.joc.5c02061 (PMC13010339; doi:10.1021/acs.joc.5c02061)
Supplement: Supplementary file 1 [file jo5c02061_si_001.pdf]

Photocatalytic Reductive Desulfonation of Aryl Tosylates

Kasmier Vicioso, Jonathan Santoro, Zachary Coren, and Rashanique D. Quarels*

Department of Chemistry and Biochemistry, Rowan University, Glassboro, NJ 08028, United States
*Corresponding author. Email quarels@rowan.edu

Supporting information

Table of Contents

1. General Considerations..S-1
2. Synthesis and Characterization of Aryl Tosylates...S-1
3. Synthesis of 10-Bromoanthrone Catalyst: S-7
4. Optimization of Reaction Conditions...S-8
5. General Procedure for the Deprotection of Aryl Tosylates S-10
6. TEMPO Radical Trapping Experiment...S-14
7. Large Scale Reaction..S-14
8. References S-15
9. NMR Spectra S-17

1. **General Considerations**

Unless otherwise noted, all commercial reagents were purchased from commercial suppliers (VWR, TCI, Ambeed Sigma, and Alfa Aesar). The inorganic bases and phenols were used without further purification. *N, N*-Diisopropylethylamine and triethylamine were stirred in calcium hydride, distilled, and store in amber vials over 3 angstrom molecular sieves in an inert atmosphere for at least 24 hours before use. All solvents were purchased from Thermo Scientific Chemicals in their inhibitor-free forms. Thin layer chromatography (TLC) was performed using aluminum-backed silica gel plates (200 μm, F254) from Silicycle, Incorporated. The visualization of the developed TLC plates was achieved using a 254-nm UV lamp. *p*-Anisaldehyde, potassium permanganate, and/or iodine solutions were used to visualize the resulting plates after development. Sodium sulfate was used as a drying agent after extractions for all reactions. Flash column chromatography was performed on silica gel (Silicycle, 40-63 microns) and eluted with hexanes/ethyl acetate. All reactions were carried out under nitrogen atmosphere in oven-dried glassware or 1-dram vials using standard Schlenk techniques.

Irradiation was performed using a single 34W Kessil PR160L-456 nm blue LED lamp (100% intensity) per experiment in borosilicate glass disposable pressure release vials. ^{1}H NMR and ^{13}C nuclear magnetic resonance (NMR) spectra were recorded on a Bruker 400 (400 and 101 MHz) spectrometer at Rowan University. All ^{1}H NMR chemical shifts are reported in parts per million relative to tetramethylsilane or residual protiated solvent as a reference and ^{13}C NMR chemical shifts are reported in parts per million relative to the solvent as a reference. ^{1}H and ^{13}C chemical shifts were internally referenced to residual solvent signals (CDCl3 referenced at δ = 7.26 and 77.02 ppm).[1] Some NMR samples run in $CDCl_3$ contain residual water from the supplier (δ = 1.56 ppm). Potassium carbonate was added to reduce the water content in the NMR solvent. Multiplicities are abbreviated as follows: singlet (s), doublet (d), triplet (t), quartet (q), doublet-doublet (dd), triplet-doublet (td), multiplet (m), and broad (br). High resolution mass spectra (HRMS) were obtained at Princeton University mass spectrometry facilities using an Agilent 6230 TOF LC/MS.

2. **Synthesis and Characterization of Aryl Tosylates.**

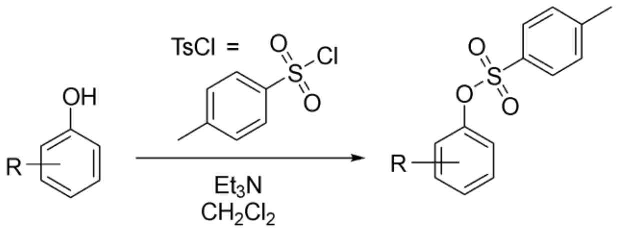

General procedure A. The following procedure is an adaptation from the method shared by Tian *et* al.[2] A stirred solution of phenol (1.0 eq) and triethylamine (Et_3N, 1.2 eq) is stirred in anhydrous dichloromethane (DCM, 0.1 M). The solution was allowed to stir for 15 minutes at room temperature. After 15 minutes, *p*-toulenesulfonyl chloride (1.1 equ) was added to the stirring solution. The reaction mixture was stirred vigorously (~500 rpm) until TLC monitoring indicated full conversion of starting phenol (typically 3-4 hours). Upon completion, the reaction was diluted with 30 mL of DCM, transferred to a separatory funnel, and washed with 30 mL of deionized water. The aqueous layer was further extracted with DCM (twice, 30 mL each). The combined

organic layers were washed with saturated brine (aq. sat. NaCl), then dried with sodium sulfate (Na_2SO_4). The resulting mixture was filtered using gravity filtration and concentrated in vacuo. The crude mixture was purified using flash column chromatography using ethyl acetate in hexanes to afford the product.

1a - 4-formylphenyl 4-methylbenzenesulfonate

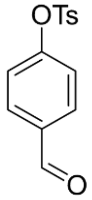

The title compound was prepared using general procedure A with 0.500 g 4-hydroxybenzaldehyde, 0.69 mL triethylamine, and 41 mL dichloromethane. The resulting residue was purified via flash chromatography(5-20% ethyl acetate/hexanes), and obtained as a white solid (0.73 g, 65%). **^{1}H NMR (400 MHz, $CDCl_3$) δ:** 9.97 (s, 1H), 7.84 (d, *J* = 8.8 Hz, 2H), 7.72 (d, *J* = 8.1 Hz, 2H), 7.33 (d, *J* = 8.0 Hz, 2H), 7.18 (d, *J* = 8.5 Hz, 2H), 2.46 (s, 3H);**^{13}C {^{1}H} NMR (101 MHz, CDCl3) δ:**190.7, 153.9, 145.9, 134.8, 132.1, 131.3, 130.0, 128.5, 123.1, 21.8; **HRMS (ESI) m/z:** [M+H]+ Calculated for $C_{14}H_{13}O_4S$ 277.0535; Found 277.0536; **MP range**: 75.5-76.9 ºC. Spectral data are consistent with those previously reported.[7]

1b - 4-formyl-2-methoxyphenyl 4-methylbenzenesulfonate

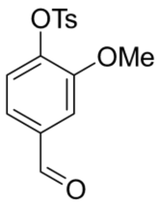

The title compound was prepared using general procedure A with 0.500 g 4-hydroxy-3-methoxybenzaldehyde, 0.55 mL triethylamine, and 33 mL dichloromethane. The resulting residue was purified via flash chromatography (5-30% ethyl acetate/hexanes), and isolated as a beige solid (0.47 g, 46%). **^{1}H NMR (400 MHz, CDCl3) δ:** 9.93 (s, 1H), 7.76 (d, *J* = 8.1 Hz, 2H), 7.43 (dd, *J* = 8.1, 1.8 Hz, 1H), 7.36 (dd, *J* = 5.1, 3.2 Hz, 2H), 7.32 (d, *J* = 8.1 Hz, 2H), 3.64 (s, 3H), 2.45 (s, 3H).; **^{13}C {^{1}H} NMR (101 MHz, CDCl3) δ:** 190.9, 152.6, 145.5, 143.1, 135.8, 133.0, 129.5, 128.6, 124.6, 124.4, 111.0, 55.8, 21.7.; **HRMS (ESI) m/z:** [M+H]+ Calculated for $C_{15}H_{15}O_5S$ 307.0640; Found 307.0647; **MP range**: 124.1-126.3 ºC. Spectral data are consistent with those previously reported.[6]

1c - methyl 3-(benzyloxy)-5-(tosyloxy)benzoate

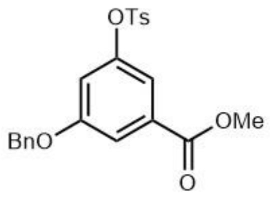

The title compound was prepared over three steps. The first two steps were adapted from a literature procedure.[3] A solution of 3,5-dihydroxybenzoic acid (1.50 g, 9.73 mmol) in 0.4 M MeOH was stirred for 10 minutes. Followed by the addition of 1.00 mL of concentrated sulfuric acid. The reaction was monitored by TLC for the consumption of carboxylic acid. Upon completion, the reaction was transferred to a separatory funnel, quenched with saturated $NaHCO_3$, extracted with ethyl acetate (3X, 50 mL each), washed brine (50 mL), dried with Na_2SO_4, and concentrated in vacuo. The residue was purified using flash chromatography (30% ethyl acetate/hexanes) to obtain methyl 3,5-dihydroxybenzoate (1.32 g, 80%). The methyl ester was dissolved in anhydrous DMF (0.3 M). Potassium carbonate (0.65 g, 4.71 mmol) was added to the solution and allowed to stir for an additional 20 minutes. Benzyl bromide (0.93 mL, 7.85 mmol) was added to the reaction mixture. The benzylation was monitored by TLC. After 18 h, the reaction was quenched with 1M HCl, extracted with ethyl acetate (3X, 100 mL each), and washed with ice water (3X, 100 mL). The residue was purified using flash

chromatography (10-30% ethyl acetate/hexanes) and the desired methyl 3-(benzyloxy)-5-hydroxybenzoate intermediate was obtained (1.07 g, 53%). The title compound was prepared using general procedure A with 1.07 g methyl 3-(benzyloxy)-5-hydroxybenzoate, 0.70 mL triethylamine, and 42 mL dichloromethane. The resulting residue was purified via flash chromatography (5-20% ethyl acetate/hexanes), and the product was isolated as a white solid (0.85 g, 50%), **^{1}H NMR (400 MHz, CDCl3) δ:** 7.71 (d, J = 8.3 Hz, 2H), 7.55 (dd, J = 2.4, 1.3 Hz, 1H), 7.42 – 7.33 (m, 5H), 7.33 – 7.29 (m, 2H), 7.27 – 7.23 (m, 2H), 6.86 (t, J = 2.3 Hz, 1H), 5.02 (s, 2H), 3.88 (s, 3H), 2.45 (s, 3H); **^{13}C {^{1}H} NMR (101 MHz, CDCl3) δ:** 165.6, 159.4, 150.2, 145.6, 135.8, 132.4, 129.9, 128.5, 127.6, 115.9, 114.4, 114.0, 70.6, 52.5, 21.7; **HRMS (ESI) m/z:** [M-H] Calculated for $C_{16}H_{15}O_6S$ 335.0595; Found 335.0592; **MP range**: 89.7-91.1 ºC

1d - 4-cyanophenyl 4-methylbenzenesulfonate
The title compound was prepared using general procedure A with 0.500 g 4-hydroxybenzonitrile, 0.70 mL triethylamine, and 33 mL dichloromethane. The resulting residue was purified via flash chromatography(5-30% ethyl acetate/hexanes), and the product was isolated as a beige solid. beige/brown solid (0.78 g, 68%), **^{1}H NMR (400 MHz, $CDCl_3$) δ:** δ 7.71 (d, *J* = 7.8 Hz, 2H), 7.61 (d, *J* = 8.1 Hz, 2H), 7.34 (d, *J* = 8.0 Hz, 2H), 7.13 (d, *J* = 8.0 Hz, 2H), 2.47 (s, 3H); **^{13}C {^{1}H} NMR (101 MHz, $CDCl_3$) δ:**152.5, 146.1, 133.9, 131.7, 130.1, 128.5, 123.5, 117.8, 111.2, 21.8 **MP range:** 90.8-92.6 ºC. Spectral data are consistent with those previously reported.[7]

1e - 4-nitrophenyl 4-methylbenzenesulfonate
The title compound was prepared using general procedure A with 0.500 g 4-nitrophenol, 0.60 mL triethylamine, and 36 mL dichloromethane. The resulting residue was purified via flash chromatography (5-30% ethyl acetate/hexanes), and the product was isolated as a white solid (0.71 g, 62%), **^{1}H NMR (400 MHz, $CDCl_3$) δ:** 8.19 (d, *J* = 9.1 Hz, 2H), 7.73 (d, *J* = 8.3 Hz, 2H), 7.35 (d, *J* = 8.2 Hz, 2H), 7.18 (d, *J* = 9.1 Hz, 2H), 2.47 (s, 3H); **^{13}C {^{1}H} NMR (101 MHz, $CDCl_3$) δ:** 153.9, 146.2, 131.8, 130.1, 128.5, 125.4, 123.2, 21.8; **MP range**: 96.5-97.3 ºC. Spectral data are consistent with those previously reported.[6]

1f – 2-(cinnamyloxy)phenyl 4-methylbenzenesulfonate

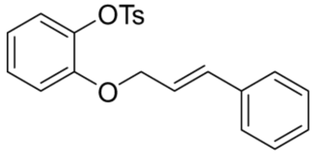

The title compound was prepared over two steps. A solution of catechol (2.00 g, 18.15 mmol) in anhydrous DMF (0.1 M) was stirred for 5 minutes. Potassium carbonate (1.76 g, 12.72 mmol) was added to the solution and allowed to stir for an additional 20 minutes. Cinnamyl bromide was added as single-portion and the reaction allowed to stir for four hours. The reaction was monitored by TLC for the consumption of the bromide. Upon completion, the reaction was quenched with HCl, extracted with ethyl acetate (3 times, 100 mL each), and washed with ice water (3 times, 100 mL). The residue was purified using flash chromatography (0-5% ethyl acetate/hexanes) and obtained as a mixture of the desired phenol and the dialkylation product. The title compound was prepared using general procedure A with 3.7 g of the alkylation mixture from step one, 2.7 mL triethylamine, and 164 mL dichloromethane. The resulting residue was purified via flash chromatography (0-10% ethyl acetate/hexanes), and isolated as a white solid in an isolated yield of 48% (3.34 g) over two steps. **^{1}H NMR (400 MHz, $CDCl_3$) δ:** 7.78 – 7.76 (d, 2H), 7.39 – 7.16 (m, 9H), 6.94 – 6.88 (m, 2H), 6.57 (dt, *J* = 16.0, 1.6 Hz, 1H), 6.14 (dt, *J* = 16.0, 5.7 Hz, 1H), 4.48 (dd, *J* = 5.7, 1.5 Hz, 2H), 2.33 (s, 3H);**^{13}C {^{1}H} NMR (101 MHz, $CDCl_3$) δ:** 150.8, 144.9, 138.7, 136.3, 133.4, 132.9, 129.4, 128.6, 128.0, 124.4, 123.7, 121.0, 114.1, 69.2, 21.6. **HRMS (ESI) m/z:** [M+Na]+ Calculated for $C_{22}H_{20}O_4SNa$ 403.0980; Found 403.0982; **MP range:** 120.7-122.2 ºC.

1g - 4-fluoro phenyl 4-methylbenzenesulfonate

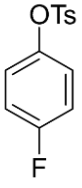

The title compound was prepared using general procedure A with 0.500 g 4-fluorophenol, 0.75 mL triethylamine, and 45 mL dichloromethane. The resulting residue was purified via flash chromatography(2-15% ethyl acetate/hexanes), and the product isolated as a white crystalline solid (0.65 g, 55%) **^{1}H NMR (400MHz, $CDCl_3$) δ:** 7.69 (d, J = 8.2 Hz, 2H), 7.32 (d, J = 8.1 Hz, 2H), 7.00 – 6.90 (m, 4H), 2.45 (s, 3H);**^{13}C {^{1}H} NMR (101 MHz, $CDCl_3$) δ:** 162.2, 159.8, 145.6, 145.4, 132.2, 129.8, 128.6, 124.1, 124.0, 116.5, 116.2, 21.7; **MP range**: 58.8-61.3 ºC. Spectral data are consistent with those previously reported.[7]

1h - 4-chlorophenyl 4-methylbenzenesulfonate

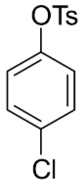

The title compound was prepared using general procedure A with 0.500 g 4-chlorophenol, 0.47 mL triethylamine, and 39 mL dichloromethane. The resulting residue was purified via flash chromatography(2-15% ethyl acetate/hexanes) and the product isolated as a white solid (0.56 g, 51%), **^{1}H NMR (400 MHz, $CDCl_3$) δ:** 7.70 (d, *J* = 8.3 Hz, 2H), 7.32 (d, *J* = 8.3 Hz, 2H), 7.25 (d, *J* = 9.0 Hz, 2H), 6.92 (d, *J* = 8.8 Hz, 2H), 2.45 (s, 3H); **^{13}C {^{1}H} NMR (101 MHz, $CDCl_3$) δ:** 148.1, 145.6, 132.8, 132.0, 129.9, 129.7, 128.6, 123.8, 21.7; **MP range**: 80.8-82.4 ºC. Spectral data are consistent with those previously reported.[5,7]

1i - 4-bromophenyl 4-methylbenzenesulfonate

The title compound was prepared using general procedure A with 0.500 g 4-bromophenol, 0.40 mL triethylamine, and 29 mL dichloromethane. The resulting residue was purified via flash chromatography (2-15% ethyl acetate/hexanes) and the product isolated as a white solid (0.15 g, 16%), **^{1}H NMR (400 MHz, $CDCl_3$) δ:** 7.69 (d, *J* = 8.3 Hz, 2H), 7.40 (d, *J* = 8.8 Hz, 2H), 7.32 (d, *J* = 8.2 Hz, 2H), 6.86 (d, *J* = 8.8 Hz, 2H), 2.45 (s, 3H); **^{13}C {^{1}H} NMR (101 MHz, $CDCl_3$) δ:**

148.6, 145.7, 132.7, 131.9, 129.9, 128.6, 124.2, 120.6, 21.8; ; **MP range**: 88.3-89.8 ºC. Spectral data are consistent with those previously reported.[7]

1j - phenyl 4-methylbenzenesulfonate

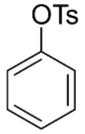

The title compound was prepared using general procedure A with 0.93 mL phenol, 1.80 mL triethylamine, and 106 mL dichloromethane. The resulting residue was purified via flash chromatographyand the product isolated as a white solid (0.63 g, 24%). **^{1}H NMR (400 MHz, $CDCl_3$) δ:** 7.70 (d, *J* = 8.1 Hz, 2H), 7.31 – 7.22 (m, 5H), 6.98 (d, *J* = 8.0 Hz, 2H), 2.45 (s, 3H); **^{13}C {^{1}H} NMR (101 MHz, $CDCl_3$) δ**: 149.7, 145.3, 132.4, 129.7, 129.6, 128.5, 127.1, 122.4, 21.7; **HRMS (ESI) m/z:** [M-H]Calculated for $C_{13}H_{12}O_3S$ 249.0585; Found 248.0573; **MP range**: 94.8-96.8 ºC. Spectral data are consistent with those previously reported.[6,7]

1k - 3-(dimethylamino)phenyl 4-methylbenzenesulfonate

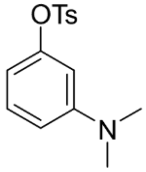

The title compound was prepared using general procedure A with 0.500 g 3-(dimethylamino)phenol, 0.61 mL triethylamine, and 37 mL dichloromethane. The resulting residue was purified via flash chromatography (10-40% ethyl acetate/hexanes), and the product isolated as a purple solid (0.56 g, 53%), **^{1}H NMR (400 MHz, $CDCl_3$) δ:** δ 7.74 (d, *J* = 8.0 Hz, 2H), 7.30 (d, *J* = 7.9 Hz, 2H), 7.07 (t, *J* = 8.2 Hz, 1H), 6.54 (d, *J* = 8.3 Hz, 1H), 6.29 (s, 1H), 6.23 (d, *J* = 8.0 Hz, 1H), 2.86 (s, 6H), 2.44 (s, 3H); **^{13}C {^{1}H} NMR (101 MHz, $CDCl_3$) δ:** 151.4, 150.8, 145.0, 132.7, 129.6, 128.6, 110.7, 109.4, 106.1, 40.3, 21.7; **HRMS (ESI) m/z:** [M+H]+ Calculated for $C_{15}H_{17}NO_3S$ 292.1007; Found 292.1013; **MP range**: 80.3-81.3 ºC.

1l - 2-methoxyphenyl 4-methylbenzenesulfonate

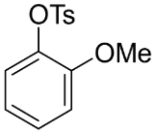

The title compound was prepared using general procedure A with 0.50 mL 2-methoxyphenol, 0.75 mL triethylamine, and 45 mL dichloromethane. The resulting residue was purified via flash chromatography(5-20% ethyl acetate/hexanes) and the product isolated as a pale yellow solid (0.99 g, 79%), **^{1}H NMR (400 MHz, $CDCl_3$) δ:** 7.75 (d, J = 8.2 Hz, 2H), 7.29 (d, J = 8.0 Hz, 2H), 7.19 (td, J = 7.9, 1.6 Hz, 1H), 7.14 (dd, J = 8.0, 1.6 Hz, 1H), 6.88 (td, J = 7.8, 1.5 Hz, 1H), 6.84 (dd, J = 8.3, 1.4 Hz, 1H), 3.56 (s, 3H), 2.44 (s, 3H); **^{13}C {^{1}H} NMR (101 MHz, $CDCl_3$) δ:** 151.8, 144.9, 138.5, 133.3, 129.3, 128.6, 128.0, 124.1, 120.6, 112.7, 55.5, 21.7; **HRMS (ESI) m/z:** [M+Na]+ Calculated for $C_{14}H_{14}O_4SNa$ 301.0510; Found 301.0512; **MP range**: 83.8-84.7 ºC. Spectral data are consistent with those previously reported.[7]

1m - 3-methoxyphenyl 4-methylbenzenesulfonate

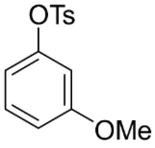

The title compound was prepared using general procedure A with 0.50 mL 2-methoxyphenol, 0.75 mL triethylamine, and 45 mL dichloromethane. The resulting residue was purified via flash chromatography(5-20% ethyl acetate/hexanes) and the product isolated as a white solid (1.02 g, 82%). **^{1}H NMR (400 MHz, $CDCl_3$) δ:** 7.73 (d, J = 8.1 Hz, 2H), 7.31 (d, J = 8.0 Hz, 2H), 7.16 (t, J = 8.1 Hz, 1H), 6.81 – 6.75 (m, 1H), 6.59 – 6.50 (m, 2H), 3.72 (s, 3H), 2.45 (s, 3H); **^{13}C {^{1}H} NMR (101 MHz, $CDCl_3$) δ:** 160.4 150.5, 145.3, 132.5, 129.9, 129.7, 128.5, 114.3, 113.1, 108.2, 55.4, 21.7; **HRMS (ESI) m/z:** [M+H]+ Calculated for $C_{14}H_{15}O_4S$ 279.0691; Found 279.0691; **MP range**: 55.8-56.6 ºC. Spectral data are consistent with those previously reported.[7]

1n - 3,4-dimethoxyphenyl 4-methylbenzenesulfonate

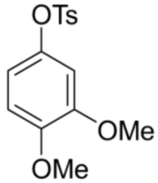

The title compound was prepared using general procedure A with 0.500 g 3,4-dimethoxyphenol, 0.54 mL triethylamine, and 32 mL dichloromethane. The resulting residue was purified via flash chromatography(5-30% ethyl acetate/hexanes) and the product isolated as a white solid (0.64 g, 64% yield). **^{1}H NMR (400 MHz, CDCl$_3$) δ:** 7.71 (d, J = 8.2 Hz, 2H), 7.31 (d, J = 8.0 Hz, 2H), 6.70 (d, J = 8.6 Hz, 1H), 6.55 – 6.44 (m, 2H), 3.84 (s, 3H), 3.74 (s, 3H), 2.45 (s, 3H); **^{13}C {^{1}H} NMR (101 MHz, CDCl$_3$) δ:** 149.2, 147.9, 145.3, 143.1, 132.4, 129.7, 128.7, 113., 110.8, 106.5, 56.1, 56.0, 21.7; **HRMS (ESI) m/z:** [M+H]+ Calculated for $C_{15}H_{17}O_5S$ 309.0797; Found 309.0794; **MP range**: 55.9-57.1 ºC.

1o - *m*-tolyl 4-methylbenzenesulfonate

The title compound was prepared using general procedure A with 0.50 mL 3-methylphenol, 0.80

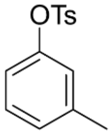

mL triethylamine, and 48 mL dichloromethane. The resulting residue was purified via flash chromatography (2-15% ethyl acetate/hexanes) and the product isolated as a white solid (0.93 g, 74%). **^{1}H NMR (400 MHz, CDCl$_3$) δ:** 7.71 (d, J = 8.3 Hz, 2H), 7.31 (d, J = 8.3 Hz, 2H), 7.14 (t, J = 7.9 Hz, 1H), 7.04 (d, J = 7.6 Hz, 1H), 6.86 (s, 1H), 6.72 (d, J = 9.4 Hz, 1H), 2.45 (s, 3H), 2.29 (s, 3H); **^{13}C {^{1}H} NMR (101 MHz, CDCl$_3$) δ:** 149.6, 145.2, 140.0, 132.6, 129.7, 129.2, 128.5, 127.8, 123.0, 119.1, 21.7, 21.2.; **HRMS (ESI) m/z:** [M+Na]+ Calculated for $C_{14}H_{14}O_3SNa$ 285.0561; Found 285.0559; **MP range**: 50.8-52.4 ºC.

1p - 3, 4-dimethylphenyl 4-methylbenzenesulfonate

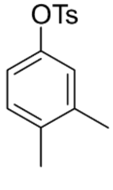

The title compound was prepared using general procedure A with 0.856 g 3,4-dimethylphenol, 1.20 mL triethylamine, and 70 mL dichloromethane. The resulting residue was purified via flash chromatography (2-15% ethyl acetate/hexanes)and the product isolated as a white solid (1.82 g, 94%). **^{1}H NMR (400 MHz, CDCl$_3$) δ:** 7.72 (d, J = 8.3 Hz, 2H), 7.31 (d, J = 8.1 Hz, 2H), 6.99 (d, J = 8.2 Hz, 1H), 6.82 (d, J = 2.5 Hz, 1H), 6.63 (dd, J = 8.3, 2.6 Hz, 1H), 2.45 (s, 3H), 2.20 (s, 3H), 2.18 (s, 3H); **^{13}C {^{1}H} NMR (101 MHz, CDCl$_3$) δ:** 147.6, 145.1, 138.2, 135.6, 132.7, 130.3, 129.7, 128.5, 123.3, 119.2, 21.7, 19.8, 19.2; **HRMS (ESI) m/z:** [M+H]+ Calculated for $C_{15}H_{17}O_3S$ 277.0898; Found 277.0910; **MP range**: 120.8-121.1 ºC.

1q - 2, 3, 5-trimethylphenyl 4-methylbenzenesulfonate

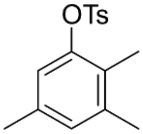

The title compound was prepared using general procedure A with 0.502 g 2,3,5-trimethylphenol, 0.62 mL triethylamine, and 37 mL dichloromethane. The resulting residue was purified via flash chromatography(2-20% ethyl acetate/hexanes) and the product isolated as a white solid (0.77 g, 72%). **^{1}H NMR (400 MHz, CDCl$_3$) δ:** 7.75 (d, *J* = 8.3 Hz, 2H), 7.32 (d, *J* = 8.1 Hz, 2H), 6.86 (s, 1H), 6.67 (s, 1H), 2.46 (s, 3H), 2.21 (s, 3H), 2.18 (s, 3H), 1.92 (s, 3H); **^{13}C {^{1}H} NMR (101 MHz, CDCl$_3$) δ:** 148.1, 145.1, 138.5, 135.9, 133.4, 129.7, 129.3, 128.4, 126.9, 120.3, 21.7, 20.8, 20.0, 12.4; **HRMS (ESI) m/z:** [M+Na]+ Calculated for $C_{16}H_{18}O_3SNa$ 313.0874; Found 313.0871; **MP range**: 91.1-92.2 ºC.

1r - 2-ethylphenyl 4-methylbenzenesulfonate

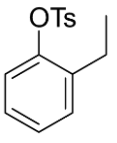

The title compound was prepared using general procedure A with 1.00 mL 2-ethylphenol, 1.38 mL triethylamine, and 83 mL dichloromethane. The resulting residue was purified via flash chromatography(2-20% ethyl acetate/hexanes) and the product isolated as a pale yellow solid (1.60g, 70%), **^{1}H NMR (400 MHz, CDCl$_3$) δ:** 7.71 (d, J

= 8.3 Hz, 2H), 7.30 (d, J = 8.1 Hz, 2H), 7.12 – 7.05 (m, 2H), 6.90 – 6.84 (m, 2H), 2.60 (q, J = 7.6 Hz, 2H), 2.45 (s, 3H), 1.20 (t, J = 7.6 Hz, 3H); **^{13}C {^{1}H} NMR (101 MHz, $CDCl_3$) δ:** 147.6, 145.2, 143.2, 132.6, 129.7, 128.9, 128.5, 122.1, 28.2, 21.7, 15.4; **HRMS (ESI) m/z:** [M+Na]+ Calculated for $C_{15}H_{16}O_3SNa$ 299.0718; Found 299.0707.; **MP range**: 64.8-66.8 °C.

1s - Napthalen-1-yl-4-methylbenzenesulfonate **;** MP range**: 75.5-76.9 °C.**

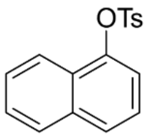

The title compound was prepared using general procedure A with 0.506 g 1-naphthol, 0.59 mL triethylamine, and 35 mL dichloromethane. The resulting residue was purified via flash chromatographyto yield a pale brown solid (0.45 g, 43%). **^{1}H NMR (400 MHz, $CDCl_3$) δ:** 7.90 (d, J = 8.3 Hz, 1H), 7.78 (td, J = 15.7, 14.5, 8.1 Hz, 4H), 7.45 (dt, J = 19.0, 7.1 Hz, 2H), 7.37 (t, J = 8.0 Hz, 1H), 7.28 (d, J = 8.1 Hz, 2H), 7.20 (d, J = 7.6 Hz, 1H), 2.42 (s, 3H); **^{13}C {^{1}H} NMR (101 MHz, $CDCl_3$) δ:** 145.8, 145.4, 134.7, 132; **MP range**: 75.5-76.9 °C..7, 129.8, 128.5, 127.7, 127.3, 127.1, 126.7, 125.1, 121.8, 118.4, 21.7; **HRMS (ESI) m/z:** [M+H] Calculated for $C_{17}H_{15}O_3S$ 299.0712; Found 299.0735; **MP range**: 87.7-89.8 °C. Spectral data are consistent with those previously reported.[5-7]

3. Synthesis of 10-Bromoanthrone Catalyst:

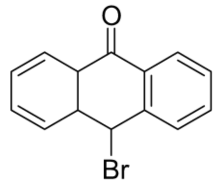

The catalyst was prepared using an adapted procedure from Schmalzbauer and coworkers.[4] Commercially available 9-anthrone (1.00 g, 5.15 mmol) was weighed and added to a 100 mL round bottom flask equipped with a stir bar. Dichloromethane (7.0 mL) was added to the round bottom flask. Separately, a stirred suspension Br_2 (1.0 mL dissolved in 4 mL CH_2Cl_2) was added dropwise in the dark within 15 minutes. One milliliter of the Br_2 solution was added to the stirring solution of 9-anthrone. The resulting mixture was vigorously stirred for 30 minutes and monitored by TLC. Upon completion by TLC, the crude mixture was concentrated *in vacuo,* filtered off and washed with hexanes (30 mL). The crude material was further purified by stirring in boiling hexanes to give the title compound as pale-yellow powder (1.01 g, 72%). The catalyst was stored in an amber container in a dry dark place to preserve its purity. **^{1}H NMR (400 MHz, $CDCl_3$) δ:** 8.28 (dd, *J* = 7.8, 1.3 Hz, 2H), 7.75 – 7.69 (m, 2H), 7.66 (td, *J* = 7.5, 1.5 Hz, 2H), 7.54 (td, *J* = 7.6, 1.3 Hz, 2H), 6.65 (s, 1H); **^{13}C {^{1}H} NMR (101 MHz, CDCl3) δ:** 183.0, 141.0, 133.6, 131.1, 130.1, 129.3, 127.9, 43.8. **HRMS (ESI) m/z:** [M-Br]+ Calculated for $C_{14}H_9O$ 193.0648; Found 193.0649; **MP range**: 122.6-125.3 °C.

Caution! 10-Bromoanthrone is light-sensitive. The spent catalyst was discarded in the organic waste as a solution in ethyl acetate upon degradation.

4. **Optimization of Reaction Conditions**

Reaction optimization was conducted according to the following procedure: an oven-dried 1 dram vial was equipped with an oven-dried Teflon-coated magnetic stir bar and charged with phenyl tosylate (0.1 mmol, 1.0 equiv.), photocatalyst (20 mol%), and base (if solid). Degassed anhydrous solvent (variable) was added via syringe and the reaction vessel was sealed with a Teflon septum and capped. Electrical tape was used to seal the sides of the cap, and the reaction vessel was frozen using liquid nitrogen, evacuated and backfilled with nitrogen gas, and allowed to thaw two times using standard Schlenk technique. If an amine base was used, a syringe was used to add the amine (3.0 equiv.) to the vial. The free-pump-thaw cycle was repeated once more. An additional piece of electrical tape was used to seal the holes in the septa. The reaction solution was irradiated by a single 34W Kessil PR160L-456 nm blue LED lamp (100% intensity) and stirred for 24 hours. Throughout irradiation, a rotary fan was placed near the to the lamp to cool the reaction setup. See the image below. Upon completion, the reaction was quenched with 1 M HCl (10 mL), transferred to a separatory funnel, and separated with 30 mL of ethyl acetate (3 times). The combined layers were washed with 30 mL of ice water (3 times), saturated brine (30 mL), and dried with sodium sulfate. The crude mixture was assessed using ^{1}H NMR spectroscopy. The ratios reported in Table 1 were obtained by integrating key identifiable peaks in the starting phenyl tosylate (doublet, 7.70 ppm) and phenol (doublet, 6.82 ppm).

Caution! This procedure requires the use of specialized visible-light sources. Precaution is required when working with the Kessil lamps and protective eyewear must be used at all times.

Caution! Extreme care should be taken both in the handling of the cryogen liquid nitrogen and its use in the Schlenk line trap to avoid the condensation of oxygen from air.

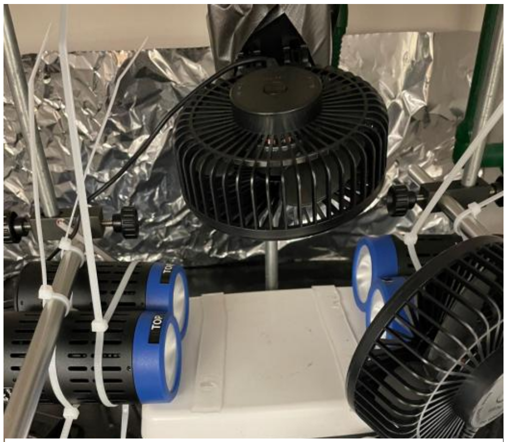

Figure S1. Photolight Setup

Table S1. Reaction optimizations were doing using phenyl tosylate 1j and 10-bromoanthrone. Optimization reactions were conducted on a 0.1 mmol scale and run as indicated in the table. Throughout the optimization, solvent, concentration, and catalyst loading were evaluated. Notably, DMSO and DIPEA were identified as the best combination for the desulfonation reaction.

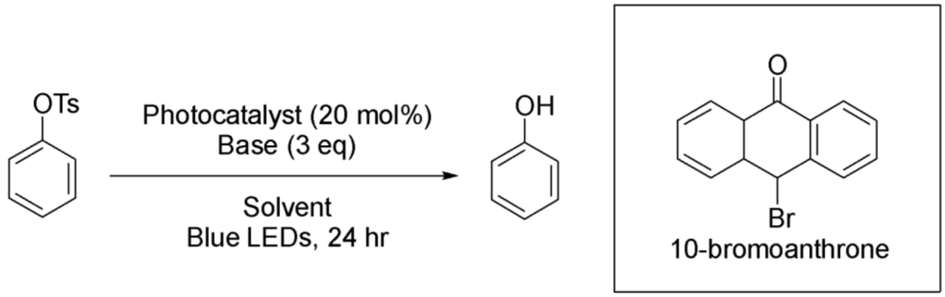

entry	scale (mmol)	base	solvent	solvent concentration (M)	catalyst mol %	time	ratio SM:product
1	0.1	K_2CO_3	DMSO	0.5	20	24 h	0.10:1
2	0.1	K_2CO_3	DMSO	0.2	20	24 h	0.14:1
3	0.1	K_2CO_3	DMSO	0.1	20	24 h	0.15:1
4	0.1	K_2CO_3	DMSO	0.03	20	24 h	0.58:1
5	0.1	K_2CO_3	DMSO	0.02	20	24 h	1.08:1
6	0.1	K_2CO_3	MeCN	0.5	20	24 h	1:0
7	0.1	K_2CO_3	DMF	0.5	20	24 h	0.2:1
8	0.1	K_2CO_3	DMA	0.5	20	24 h	1:0
9	0.1	K_2CO_3	Toluene	0.5	20	24 h	0.16:1
10	0.1	K_2CO_3	THF	0.5	20	24 h	0.48:1
11	0.1	Et_3N	DCM	0.5	20	24 h	0.69:1
12	0.1	Et_3N	DMSO	0.5	20	24 h	0.19:1
13	0.1	DIPEA	DMSO	0.5	20	24 h	0.08:1
14	0.1	K_3PO_4	DMSO	0.5	20	24 h	0.56:1
15	0.1	$NaHCO_3$	DMSO	0.5	20	24 h	4.53:1
16	0.1	DIPEA	DMSO	0.5	10	24 h	0.22:1
17	0.1	DIPEA	DMSO	0.5	30	24 h	0.07:1

5. General Procedure for the Deprotection of Aryl Tosylates

General Procedure B. Reaction optimization was conducted according to the following procedure: an oven-dried 1 dram vial was equipped with an oven-dried Teflon-coated magnetic stir bar and charged with phenyl tosylate (0.20 mmol, 1.0 equiv.) and 10-bromoanthrone (0.0109 g, 0.04 mmol, 0.2 equiv). Degassed anhydrous solvent (2.0 mL, 0.1 M) was added via syringe and the reaction vessel was sealed with a cap. Electrical tape was used to seal the sides of the cap, and the reaction vessel was frozen using liquid nitrogen, evacuated and backfilled with nitrogen gas, and allowed to thaw two times using standard Schlenk technique. DIPEA (0.105 mL, 0.60 mmol, 3.0 equiv.) was added via syringe to the vial. The free-pump-thaw cycle was repeated once more. An additional piece of electrical tape was used to seal the holes in the septa. The reaction solution was irradiated by a single 34W Kessil PR160L-456 nm blue LED lamp (100% intensity) and stirred for 24 hours. The reaction vials were positioned approximately 1.0 inch away from the light source using double-sided tape. Throughout irradiation, a rotary fan was placed near the to the lamp to cool the reaction setup. See Figure S1. Upon completion, the reaction was quenched with 1 M HCl (10 mL), transferred to a separatory funnel, and separated with 30 mL of ethyl acetate (3 times). The combined layers were washed with 30 mL of ice water (3 times), saturated brine (30 mL), and dried with sodium sulfate. The crude mixture was assessed using ^{1}H NMR spectroscopy. The reactions were run in triplicate. The internal standard 1,3,5-trimethoxybenzene was added to one of the three reactions before extraction with ethyl acetate. The phenol products were isolated using flash column chromatography (ethyl acetate/hexanes) and confirmed via NMR and TLC.

2a – 4-hydroxybenzaldehyde

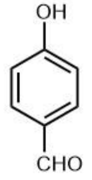

The desulfonation reaction was run using general procedure B and substrate 1a (0.0552 g, 0.2 mmol). A gradient method was used to purify 4-hydroxybenzaldehyde – 100% hexanes up to 30% ethyl acetate in hexanes. A yellow solid was isolated (20.0 mg, 82%). ^{1}H NMR (400 MHz, Chloroform-*d*) δ 9.87 (s, 1H), 7.82 (d, *J* = 8.7 Hz, 2H), 6.97 (d, *J* = 8.6 Hz, 2H), 6.14 (s, 1H); R_f= 0.44 in 30% EtOAc/Hex. The NMR spectra of the purified product matched the NMR of the commercially available 4-hydroxybenzaldehyde.

2b – 4-hydroxy-3-methoxybenzaldehyde

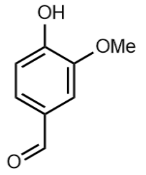

The desulfonation reaction was run using general procedure B and substrate 1b (0.0612 g, 0.2 mmol). A gradient method was used to purify the product – 100% hexanes up to 40% ethyl acetate in hexanes. An off-white solid was isolated (17.9 mg, 58%). ^{1}H NMR (400 MHz, Chloroform-*d*) δ 9.83 (s, 1H), 7.47 – 7.37 (m, 2H), 7.04 (d, *J* = 8.4 Hz, 1H), 6.20 (s, 1H), 3.97 (s, 3H); R_f= 0.60 in 40% EtOAc/Hex. The NMR spectra of the purified product matched the NMR of the commercially available 4-hydroxy-3-methoxybenzaldehyde.

2c – methyl 3-(benzyloxy)-5-hydroxybenzoate

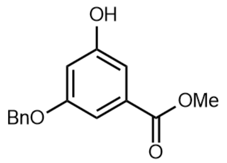

The desulfonation reaction was run using general procedure B and substrate 1c (0.0825 g, 0.2 mmol). A gradient method was used to purify the crude reaction mixture – 100% hexanes up to 30% ethyl acetate in hexanes. A white solid was isolated (48.0 mg, 93%). ^{1}H NMR (400 MHz, Chloroform-*d*) δ 7.46 – 7.29 (m, 5H), 7.24 (dd, *J* = 2.3, 1.2 Hz, 1H), 7.17 (d, *J* = 1.9 Hz, 1H), 6.69 (t, *J* = 2.3 Hz, 1H), 5.05 (s, 2H), 3.89 (s, 4H); ^{13}C NMR (101 MHz, Chloroform-*d*) δ 167.1, 160.0, 136.5, 132.0, 128.6, 128.1, 127.6,

109.6, 107.9, 107.5, 70.2, 52.3; R_f= 0.17 in 20% EtOAc/Hex. **HRMS (ESI) m/z:** [M+Na]+ Calculated for $C_{15}H_{14}NaO_4$ 281.0790 ; Found 281. 0787; **MP range**: 95.8-97.2 ºC .

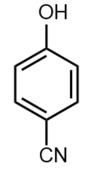

2d – 4-hydroxybenzonitrile

The desulfonation reaction was run using general procedure B and substrate 1d (0.0547 g, 0.2 mmol). A gradient method was used to purify the reaction mixture – 100% hexanes up to 15% ethyl acetate in hexanes. A beige-yellow solid was isolated (18.5 mg, 78%). ^{1}H NMR (400 MHz, Chloroform-*d*) δ 7.56 (d, *J* = 8.5 Hz, 2H), 6.93 (d, *J* = 8.6 Hz, 2H), 6.02 (s, 1H)); R_f= 0.45 in 30% EtOAc/Hex. The NMR spectra of the purified product matched the NMR of the commercially available 4-hydroxybenzonitrile.

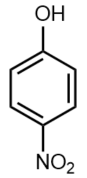

2e – 4-nitrophenol

The desulfonation reaction was run using general procedure B and substrate 1e (0.0586 g, 0.2 mmol). A gradient method was used to purify the crude reaction mixture – 100% hexanes up to 15% ethyl acetate in hexanes. A yellow solid was isolated (13.9 mg, 50%). ^{1}H NMR (400 MHz, Chloroform-d) δ 8.18 (d, *J* = 9.1 Hz, 2H), 6.93 (d, *J* = 9.0 Hz, 2H), 5.76 (s, 1H)); R_f= 0.21 in 20% EtOAc/Hex. The NMR spectra of the purified product matched the NMR of the commercially available 4-nitrophenol.

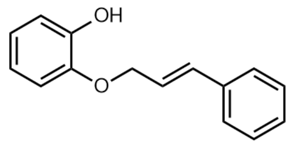

2f – 2-(cinnamyloxy)phenol

The desulfonation reaction was run using general procedure B and substrate 1f (0.0761 g, 0.2 mmol). A gradient method was used to purify the crude reaction mixture – 100% hexanes up to 15% ethyl acetate in hexanes. A white solid was isolated (33.1 mg, 73%). ^{1}H NMR (400 MHz, Chloroform-*d*) δ 7.46 – 7.39 (m, 2H), 7.38 – 7.31 (m, 2H), 7.31 – 7.27 (m, 1H), 6.99 – 6.80 (m, 4H), 6.77 – 6.67 (m, 1H), 6.43 (dt, *J* = 15.9, 6.0 Hz, 1H), 5.69 (s, 1H), 4.77 (dd, *J* = 6.0, 1.4 Hz, 2H); ^{13}C {^{1}H}NMR (101 MHz, Chloroform-*d*) δ 146.0, 145.6, 136.1, 133.9, 128.7, 128.2, 126.6, 123.8, 121.8, 120.1, 114.8, 112.3, 68.8; R_f = 0.36 in 10% EtOAc/Hex. HRMS (ESI) m/z: [M+Na]+ Calculated for $C_{15}H_{14}NaO_2$ 249.0891; Found 249.0896; MP range: 53.8-54.9 ºC.

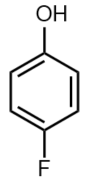

2g – 4-fluorophenol

The desulfonation reaction was run using general procedure B and substrate 1g (0.0533 g, 0.2 mmol). A gradient method was used to purify the crude reaction mixture – 100% hexanes up to 15% ethyl acetate in hexanes. A white solid was isolated (3.6 mg, 16%). ^{1}H NMR (400 MHz, Chloroform-d) δ 6.93 (t, J = 8.6 Hz, 2H), 6.81 – 6.71 (m, 2H), 4.45 (s, 1H); R_f= 0.41 in 12% EtOAc/Hex. The NMR spectra of the purified product matched the NMR of the commercially available 4-fluorophenol.

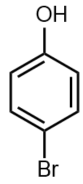

2i – 4-bromophenol

The desulfonation reaction was run using general procedure B and substrate 1i (0.0654 g, 0.2 mmol). A gradient method was used to purify the crude reaction mixture – 100% hexanes up to 15% ethyl acetate in hexanes. A white solid was isolated (25.8 mg, 75%). ^{1}H NMR (400 MHz, Chloroform-*d*) δ 7.34 (d, *J* = 8.7 Hz, 2H), 6.72 (d, *J* = 8.8 Hz, 2H), 4.45 (s, 1H); R_f= 0.27 in 12% EtOAc/Hex. The NMR spectra of the purified product matched the NMR of the commercially available 4-bromophenol.

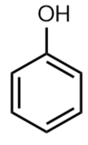

2j – phenol

The desulfonation reaction was run using general procedure B and substrate 1j (0.0497 g, 0.2 mmol). A gradient method was used to purify the crude reaction mixture – 100% hexanes up to 15% ethyl acetate in hexanes. A pinkish-white amorphous solid was isolated (15.5 mg, 82%). ^{1}H NMR (400 MHz, Chloroform-*d*) δ 7.36 – 7.19 (m, 2H), 6.94 (t, *J* = 7.3 Hz, 1H), 6.83 (d, *J* = 8.0 Hz, 2H), 4.72 (s, 1H); R_f= 0.50 in 10% EtOAc/Hex. The NMR spectra of the purified product matched the NMR of the commercially available phenol.

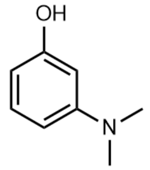

2k –3-(dimethylamino)phenol

The desulfonation reaction was run using general procedure B and substrate 1k (0.0583 g, 0.2 mmol). A gradient method was used to purify the crude reaction mixture – 100% hexanes up to 25% ethyl acetate in hexanes. A brown solid was isolated (13.5 mg, 49%). ^{1}H NMR (400 MHz, Chloroform-*d*) δ 7.09 (t, *J* = 8.0 Hz, 1H), 6.33 (dd, *J* = 8.4, 2.2 Hz, 1H), 6.26 – 6.14 (m, 2H), 2.92 (s, 6H); R_f= 0.33 in 20% EtOAc/Hex. The NMR spectra of the purified product matched the NMR of the commercially available 3-(dimethylamino)phenol.

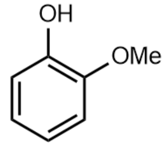

2l – 2-methoxyphenol

The desulfonation reaction was run using general procedure B and substrate 1l (0.0558 g, 0.2 mmol). A gradient method was used to purify the crude reaction mixture – 100% hexanes up to 10% ethyl acetate in hexanes. A light-yellow tinted oil was isolated (11.4 mg, 74%). ^{1}H NMR (400 MHz, Chloroform-d) δ 6.97 – 6.91 (m, 1H), 6.90 – 6.80 (m, 3H), 5.62 (bs, 1H), 3.89 (s, 3H); R_f= 0.68 in 20% EtOAc/Hex. The NMR spectra of the purified product matched the NMR of the commercially available 2-methoxyphenol.

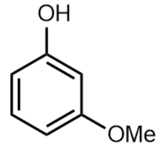

2m – 3-methoxyphenol

The desulfonation reaction was run using general procedure B and substrate 1m (0.0557 g, 0.2 mmol). A gradient method was used to purify the crude reaction mixture – 100% hexanes up to 20% ethyl acetate in hexanes. A pale-yellow oil was isolated (20.4 mg, 84%). ^{1}H NMR (400 MHz, Chloroform-*d*) δ 7.14 (t, *J* = 8.1 Hz, 1H), 6.53 – 6.47 (m, 1H), 6.47 – 6.40 (m, 2H), 4.81 (s, 1H), 3.77 (s, 3H); R_f= 0.54 in 10% EtOAc/Hex. The NMR spectra of the purified product matched the NMR of the commercially available 3-methoxyphenol.

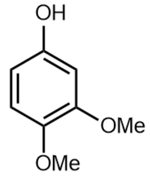

2n – 3,4-dimethoxyphenol

The desulfonation reaction was run using general procedure B and substrate 1n (0.0618 g, 0.2 mmol). A gradient method was used to purify the crude reaction mixture – 100% hexanes up to 30% ethyl acetate in hexanes. A yellowish-white solid was isolated (25.3 mg, 82%). ^{1}H NMR (400 MHz, Chloroform-*d*) δ 6.73 (d, *J* = 8.6 Hz, 1H), 6.47 (d, *J* = 2.8 Hz, 1H), 6.34 (dd, *J* = 8.0 Hz, *J* = 4.0 Hz, 1H), 4.45 (s, 1H), 3.85 (s, 3H), 3.83 (s, 3H); R_f= 0.25 in 30% EtOAc/Hex. The NMR spectra of the purified product matched the NMR of the commercially available 3,4-dimethoxyphenol.

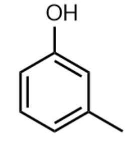

2o – 3-methylphenol

The desulfonation reaction was run using general procedure B and substrate 1o (0.0525 g, 0.2 mmol). A gradient method was used to purify the crude reaction mixture – 100% hexanes up to 30% ethyl acetate in hexanes. A yellow oil was isolated (16.5 mg, 79%). ^{1}H NMR (400 MHz, Chloroform-*d*) δ 7.14 (t, *J* = 7.7 Hz, 1H), 6.77 (d, *J* = 7.6 Hz, 1H), 6.66 (d, *J* = 10.4 Hz, 2H), 4.35 (s, 1H), 2.32 (s, 3H); R_f= 0.24 in 20% EtOAc/Hex. The NMR spectra of the purified product matched the NMR of the commercially available 3-methylphenol.

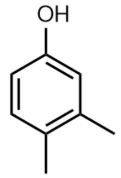

2p – 3,4-dimethylphenol

The desulfonation reaction was run using general procedure B and substrate 1p (0.0553 g, 0.2 mmol). A gradient method was used to purify the crude reaction mixture – 100% hexanes up to 10% ethyl acetate in hexanes. A white solid was isolated (22.6 mg, 92%). ^{1}H NMR (400 MHz, Chloroform-*d*) δ 6.98 (d, *J* = 8.1 Hz, 1H), 6.64 (d, *J* = 2.7 Hz, 1H), 6.57 (dd, *J* = 8.1, 2.7 Hz, 1H), 4.48 (d, *J* = 2.0 Hz, 1H), 2.21 (s, 3H), 2.18 (s, 3H); R_f= 0.71 in 20% EtOAc/Hex. The NMR spectra of the purified product matched the NMR of the commercially available 3,4-dimethylphenol.

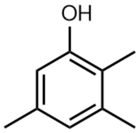

2q – 2,3,5-trimethylphenol

The desulfonation reaction was run using general procedure B and substrate 1q (0.0581 g, 0.2 mmol). A gradient method was used to purify the crude reaction mixture – 100% hexanes up to 15% ethyl acetate in hexanes. A white solid was isolated (22.0 mg, 81%). ^{1}H NMR (400 MHz, Chloroform-d) δ 6.59 (s, 1H), 6.49 – 6.43 (m, 1H), 4.53 (s, 1H), 2.24 (s, 6H), 2.12 (s, 3H); R_f= 0.26 in 20% EtOAc/Hex. The NMR spectra of the purified product matched the NMR of the commercially available 2,3,5-trimethylphenol.

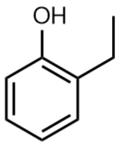

2r – 2-ethylphenol

The desulfonation reaction was run using general procedure B and substrate 1r (0.0553 g, 0.2 mmol). A gradient method was used to purify the crude reaction mixture – 100% hexanes up to 10% ethyl acetate in hexanes. A clear oil was isolated (16.4 mg, 67%). ^{1}H NMR (400 MHz, Chloroform-*d*) δ 7.07 (d, *J* = 8.1 Hz, 2H), 6.76 (d, *J* = 8.4 Hz, 2H), 4.58 (d, *J* = 6.6 Hz, 1H), 2.58 (q, *J* = 7.6 Hz, 2H), 1.20 (t, *J* = 7.6 Hz, 3H); R_f= 0.24 in 20% EtOAc/Hex. The NMR spectra of the purified product matched the NMR of the commercially available 2-ethylphenol.

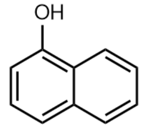

2s – 1-naphthol

The desulfonation reaction was run using general procedure B and substrate 1s (0.0597 g, 0.2 mmol). A gradient method was used to purify the crude reaction mixture – 100% hexanes up to 15% ethyl acetate in hexanes. A dark brown solid was isolated (24.5 mg, 85%). ^{1}H NMR (400 MHz, Chloroform-*d*) δ 8.25 – 8.11 (m, 1H), 7.87–7.73 (m, 1H), 7.55–7.47 (m, 2H), 7.45 (d, J = 8.3 Hz, 1H), 7.31 (t, J = 7.9 Hz, 1H), 6.82 (d, J = 7.4 Hz, 1H), 5.23 (s, 1H); R_f= 0.26 in 10% EtOAc/Hex. The NMR spectra of the purified product matched the NMR of the commercially available 1-naphthol .

6. TEMPO Radical Trapping Experiment

An oven-dried 1 dram vial was equipped with a Teflon-coated magnetic stir bar and charged with phenyl tosylate (0.2 mmol, 1.0 equiv), TEMPO (0.3 mmol, 1.5 equiv), and 10-bromoanthrone (20 mol%). Degassed anhydrous solvent (0.1 M) was added via syringe and the reaction vessel was sealed with a cap and wrapped with electrical tape. The solution in the reaction vessel was frozen using liquid nitrogen, evacuated and backfilled with nitrogen gas, and allowed to thaw two times using the standard Schlenk technique. DIPEA (0.105 mL, 0.6 mmol, 3.0 equiv) was added via syringe to the vial. The free-pump-thaw cycle was repeated once more. An additional piece of electrical tape was used to seal the holes in the septa. The reaction solution was irradiated using 34W Kessil PR160L-456 nm blue LED lamps (100% intensity) and stirred for 24 hours. The reaction was quenched and purified as outlined in General Procedure B. The crude reaction mixture was purified via column chromatography using a gradient system of 2-10% EtOAc/Hexanes. The column fractions were collected, concentrated and characterized by TLC and NMR. The recovered starting material (0.0523 g of 4-formylphenyl 4-methylbenzenesulfonate) was isolated in a 96% yield. The NMR of the recovered starting material matched previous NMR data. An unknown material was isolated from the column (0.0057 g, R_f=0.68 in 20%EtOAc/Hexanes) and subjected to HRMS. A mass of 450.1067 m/z was observed. This mass most closely matched the [M+Na] peak for the TEMPO-catalyst adduct below. The accompanying MS and NMR spectra are also included in the SI.

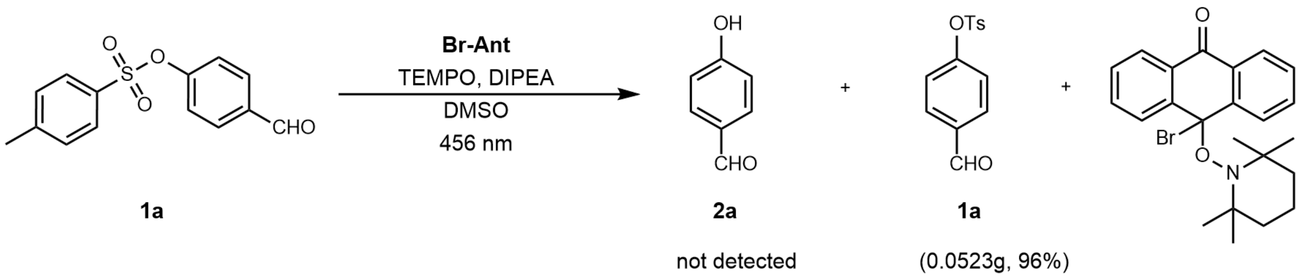

7. Large Scale Reaction (10x)

Reaction was conducted according to general procedure B: an oven-dried 100 mL round bottom flask was equipped with a Teflon-coated magnetic stir bar and charged with phenyl tosylate (0.5520 g, 2.0 mmol, 1.0 equiv.) and 10-bromoanthrone (0.1092 g, 0.2 equiv).

Degassed anhydrous solvent (20.0 mL, 0.1 M) was added via syringe and the reaction vessel was sealed with a Teflon septum. The reaction vessel was frozen using liquid nitrogen, evacuated and backfilled with nitrogen gas, and allowed to thaw two times using standard Schlenk technique. After the second cycle, DIPEA (1.05 mL, 6.0 mmol, 3.0 equiv) was added via syringe to the vial. The free-pump-thaw cycle was repeated once more. An additional piece of electrical tape was used to seal the holes in the septa. The reaction solution was irradiated by a single 34W Kessil PR160L-456 nm blue LED lamp (100% intensity) and stirred for 24 hours. Using a clamp, the round bottom flask was positioned approximately one inch away from the light source. Throughout irradiation, a rotary fan was placed near the to the lamp to cool the reaction setup. After 24 hours, the reaction was quenched with 1 M HCl (60 mL), transferred to a separatory funnel, and separated with 100 mL of ethyl acetate (3x). The combined layers were washed with 100 mL of ice water (3x), saturated brine (100 mL)The crude reaction mixture was purified via column chromatography using 60 grams of silica gel and a gradient system of 2-10% EtOAc/Hexanes. The column fractions were collected, concentrated and characterized by TLC and NMR. The desired 4-hydroxybenzaldehyde was isolated as a yellow solid (0.1703 g, 70%). Additionally, 0.1604 g of recovered 4-formylphenyl 4-methylbenzenesulfonatewas also isolated from the column.

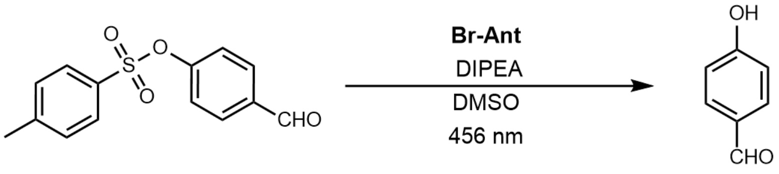

1a (0.552 g, 2.0 mmol)

2a (0.1703 g, 70%)

8. References

1. Babij, N.R.; McCusker, E. O.; Whiteker, G. T.; Canturk, B.; Choy, N.; Creemer, L.C.; De Amicis, C. V.; Hewlett, N.M.; Johnson, P.L.; Knobelsdorf, J.A.; Li, F.; Lorsbach, B.A.; Nugent, B.M.; Ryan, S.J.; Smith, M.R.; Yang, Q., NMR Chemical Shifts of Trace Impurities: Industrially Preferred Solvents Used in Process and Green Chemistry, *Org. Process Res. Dev.,* **2016** *20*, 661-667.
2. Tian, Y.-E. Di Sun, D.; Han, X.-X.; Yang, J.-M.; Zhang, S.; Feng, N.-N.; Zhu, L.-N.; Xu, Z.-Y.; Che, Z-P.; Liu, S.-M.; Lin, X.-M.; Jiang, J.; Chen, G.-Q., Synthesis, anti-oomycete activity, and SAR studies of paeonol derivatives, *J. Asian Nat. Prod. Res.,* **2021**, *23*, 138-149.
3. Sharif, E. U. and O'Doherty, G. A.,Regioselective bromination: An approach to the D-ring of the gilvocarcin antibiotics, *Heterocycles*, **2014**, *88*, 1275-1285.
4. Schmalzbauer, M.; Ghosh, I.; Konig, B., Utilising excited state organic anions for photoredox catalysis: activation of (hetero)aryl chlorides by visible light-absorbing 9-anthrolate anion, *Faraday Discuss.,* **2019**, *215*, 364-378.

5. Entz, E. D.; Russell, J. E. A.; Hooker, L. V.; Neufeldt, S. R.; Small Phosphine Ligands Enable Selective Oxidative Addition of Ar—O over Ar—Cl Bonds at Nickel(0), *J. Am. Chem. Soc.*, **2020**, *142*, 15454–15463.
6. Dhonthulachitty, C.; Kothakapu, S. R.; Neella, C. K., An efficient practical tosylation of phenols, amines, and alcohols employing mild reagent [DMAPTs]+Cl−, *Tetrahedron Letters*, **2016**, *57*, 4620-4623.
7. Muze Lin, M.; Luo, J.; Xie, Y.; Du, G.; Cai, Z.; Dai, B.; He, L., SuFEx Reactions of Sulfonyl Fluorides, Fluorosulfates, and Sulfamoyl Fluorides Catalyzed by N-Heterocyclic Carbenes, *ACS Catal*. **2023**, 13, 14503-14512.

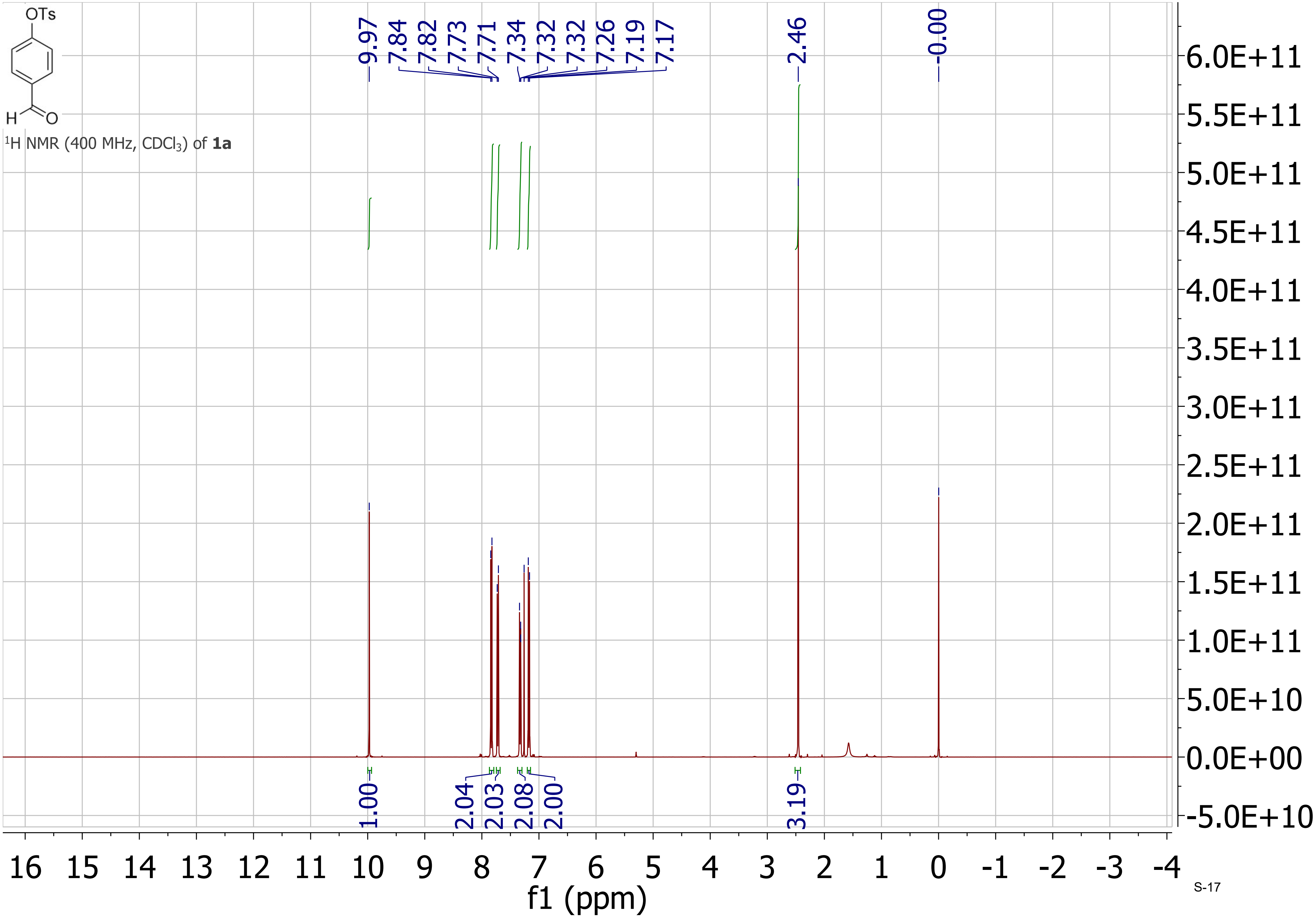
OTs
H
O
¹H NMR (400 MHz, CDCl3) of 1a
9.97
7.84
7.82
7.73
7.71
7.34
7.32
7.32
7.26
7.19
7.17
2.46
-0.00
1.00
2.04
2.03
2.08
2.00
3.19
6.0E+11
5.5E+11
5.0E+11
4.5E+11
4.0E+11
3.5E+11
3.0E+11
2.5E+11
2.0E+11
1.5E+11
1.0E+11
5.0E+10
0.0E+00
-5.0E+10
16 15 14 13 12 11 10 9 8 7 6 5 4 3 2 1 0 -1 -2 -3 -4
f1 (ppm)

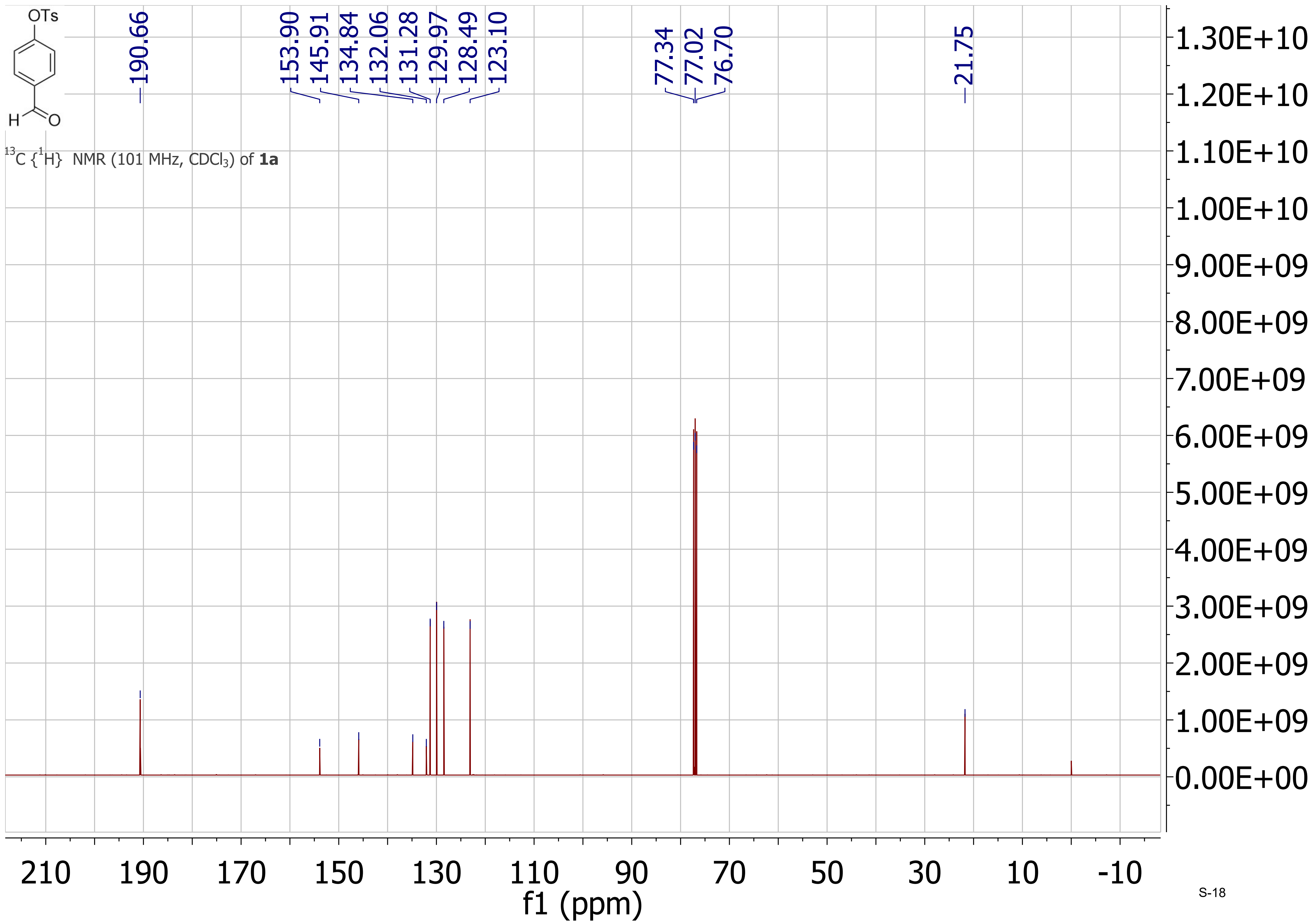

OTs
H
O
190.66
153.90
145.91
134.84
132.06
131.28
129.97
128.49
123.10
77.34
77.02
76.70
21.75
$^{13}C\{^{1}H\}$ NMR (101 MHz, $CDCl_3$) of **1a**
1.30E+10
1.20E+10
1.10E+10
1.00E+10
9.00E+09
8.00E+09
7.00E+09
6.00E+09
5.00E+09
4.00E+09
3.00E+09
2.00E+09
1.00E+09
0.00E+00
210
190
170
150
130
110
90
70
50
30
10
-10
f1 (ppm)

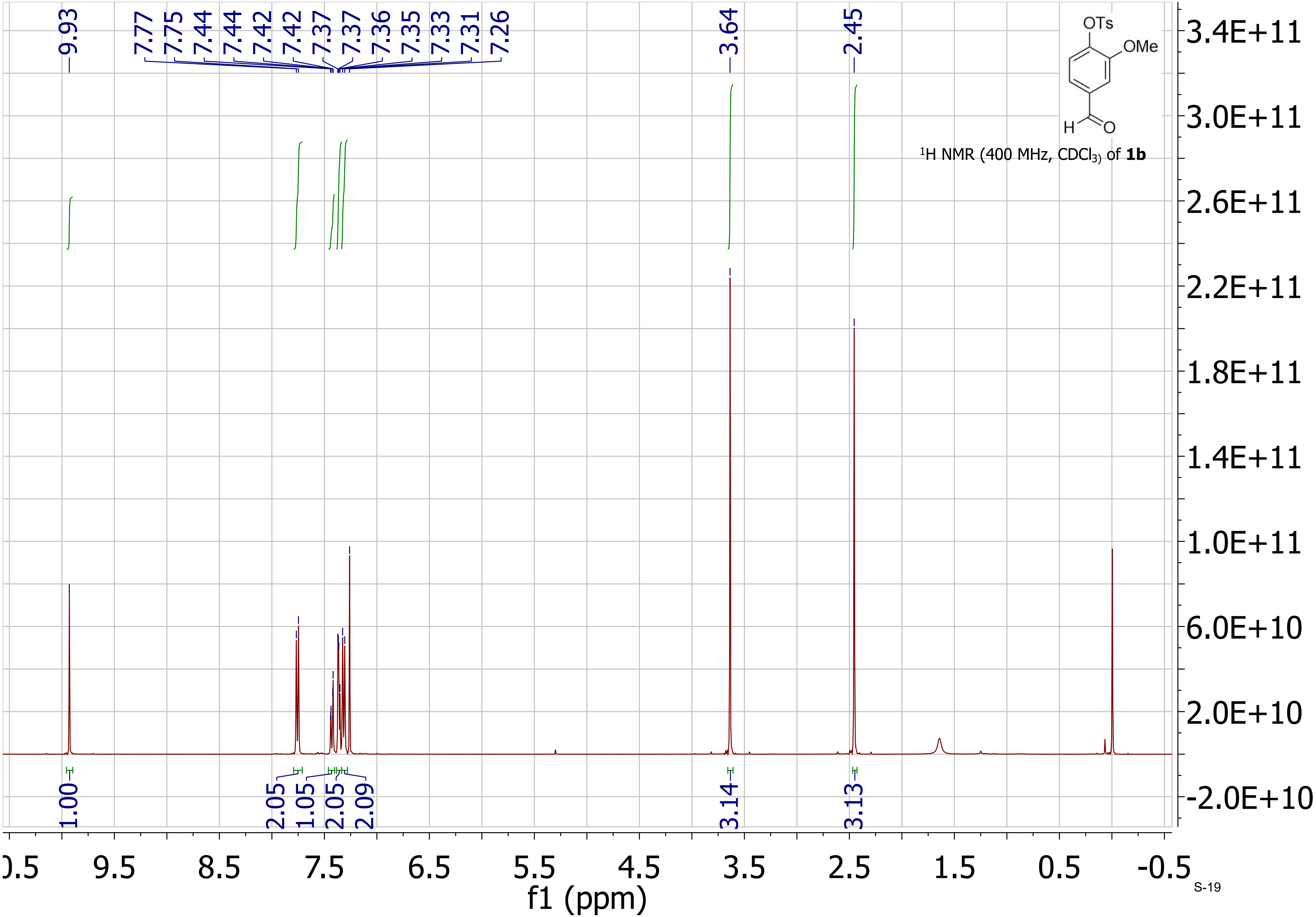
9.93
7.77
7.75
7.44
7.44
7.42
7.42
7.37
7.37
7.36
7.35
7.33
7.31
7.26
3.64
2.45
OTs
OMe
H
O
1H NMR (400 MHz, CDCl3) of 1b
1.00
2.05
1.05
2.05
2.09
3.14
3.13
3.4E+11
3.0E+11
2.6E+11
2.2E+11
1.8E+11
1.4E+11
1.0E+11
6.0E+10
2.0E+10
-2.0E+10
9.5
8.5
7.5
6.5
5.5
4.5
3.5
2.5
1.5
0.5
-0.5
f1 (ppm)

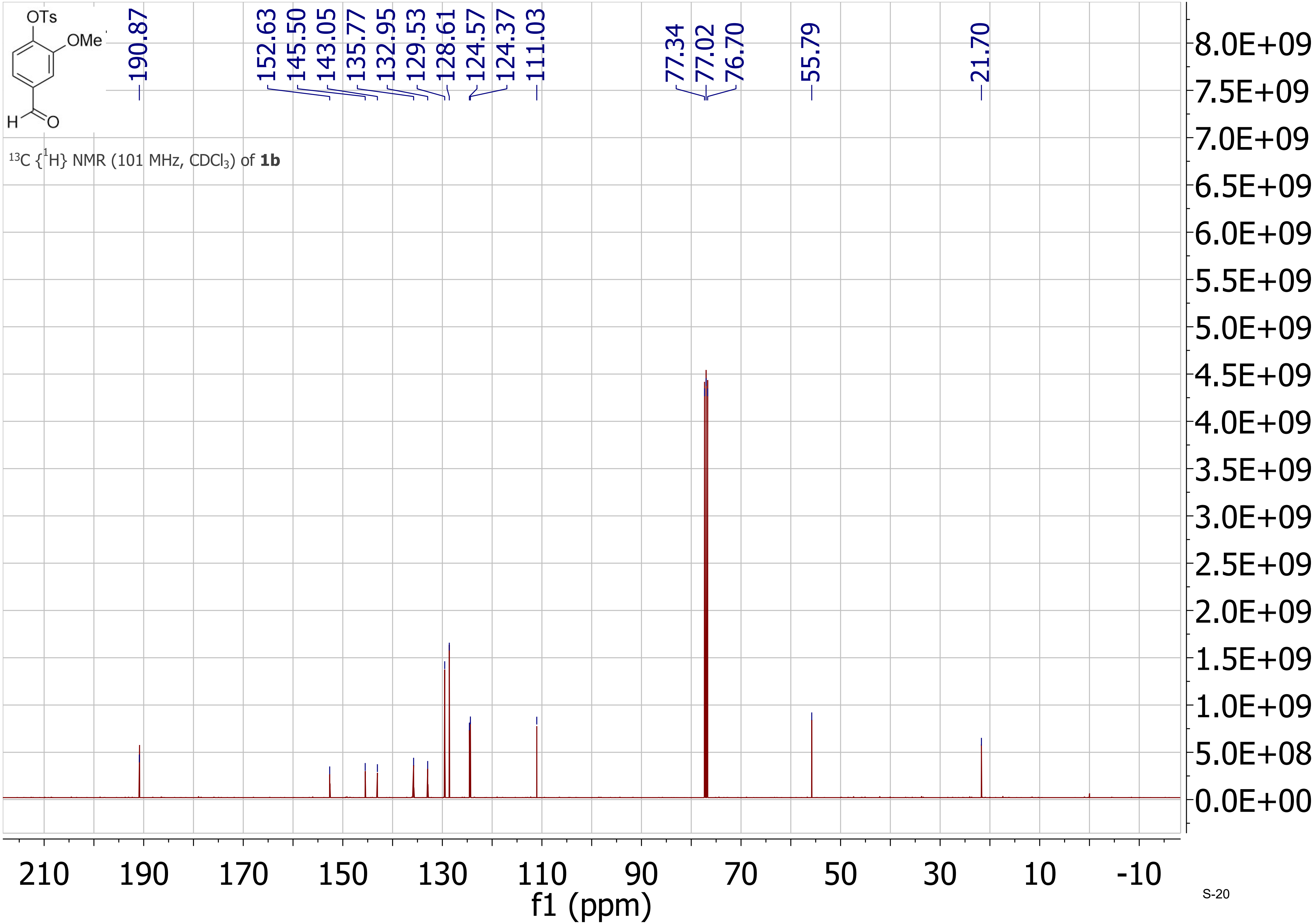
OTs
OMe
H
O
190.87
152.63
145.50
143.05
135.77
132.95
129.53
128.61
124.57
124.37
111.03
77.34
77.02
76.70
55.79
21.70
13C {1H} NMR (101 MHz, CDCl3) of 1b
8.0E+09
7.5E+09
7.0E+09
6.5E+09
6.0E+09
5.5E+09
5.0E+09
4.5E+09
4.0E+09
3.5E+09
3.0E+09
2.5E+09
2.0E+09
1.5E+09
1.0E+09
5.0E+08
0.0E+00
210
190
170
150
130
110
90
70
50
30
10
-10
f1 (ppm)

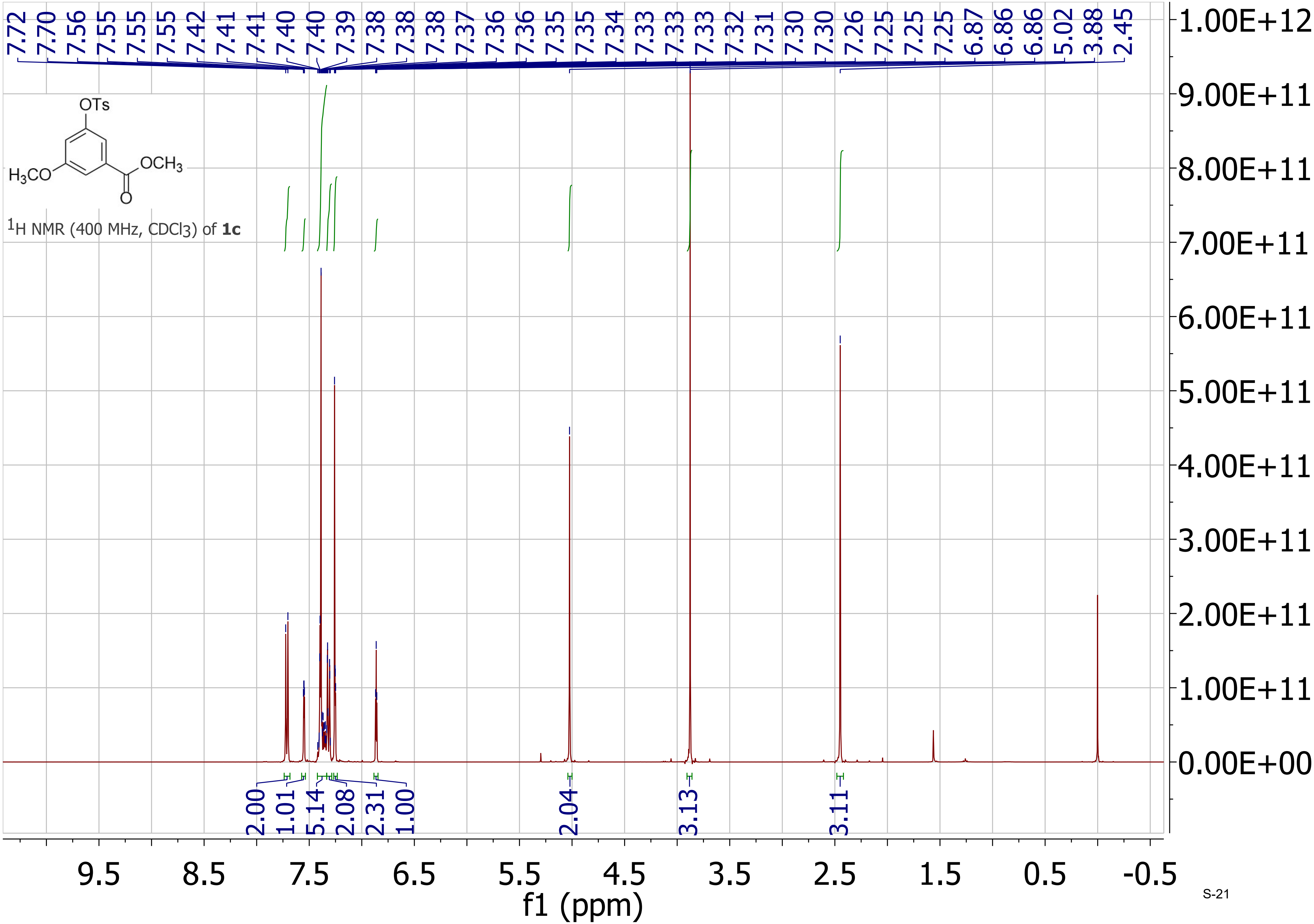
OTs
H3CO
OCH3
O
1H NMR (400 MHz, CDCl3) of 1c
7.72
7.70
7.56
7.55
7.55
7.55
7.42
7.41
7.41
7.40
7.40
7.39
7.38
7.38
7.38
7.37
7.36
7.36
7.35
7.35
7.34
7.33
7.33
7.33
7.32
7.31
7.30
7.30
7.26
7.25
7.25
7.25
6.87
6.86
6.86
5.02
3.88
2.45
1.00E+12
9.00E+11
8.00E+11
7.00E+11
6.00E+11
5.00E+11
4.00E+11
3.00E+11
2.00E+11
1.00E+11
0.00E+00
2.00
1.01
5.14
2.08
2.31
1.00
2.04
3.13
3.11
9.5
8.5
7.5
6.5
5.5
4.5
3.5
2.5
1.5
0.5
-0.5
f1 (ppm)

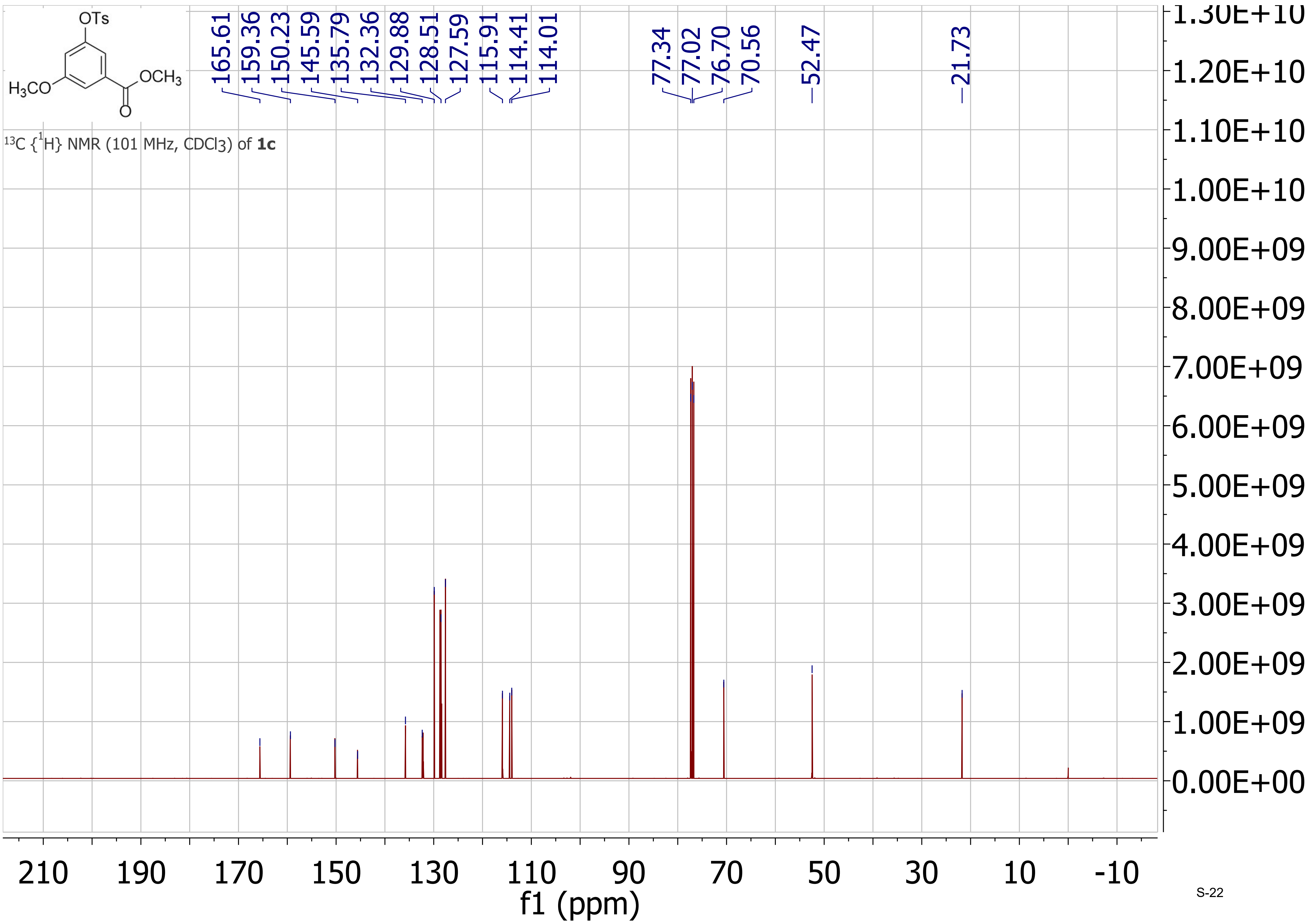

OTs
H_3CO
OCH_3
O
^{13}C {^{1}H} NMR (101 MHz, $CDCl_3$) of **1c**
165.61
159.36
150.23
145.59
135.79
132.36
129.88
128.51
127.59
115.91
114.41
114.01
77.34
77.02
76.70
70.56
52.47
21.73
f1 (ppm)

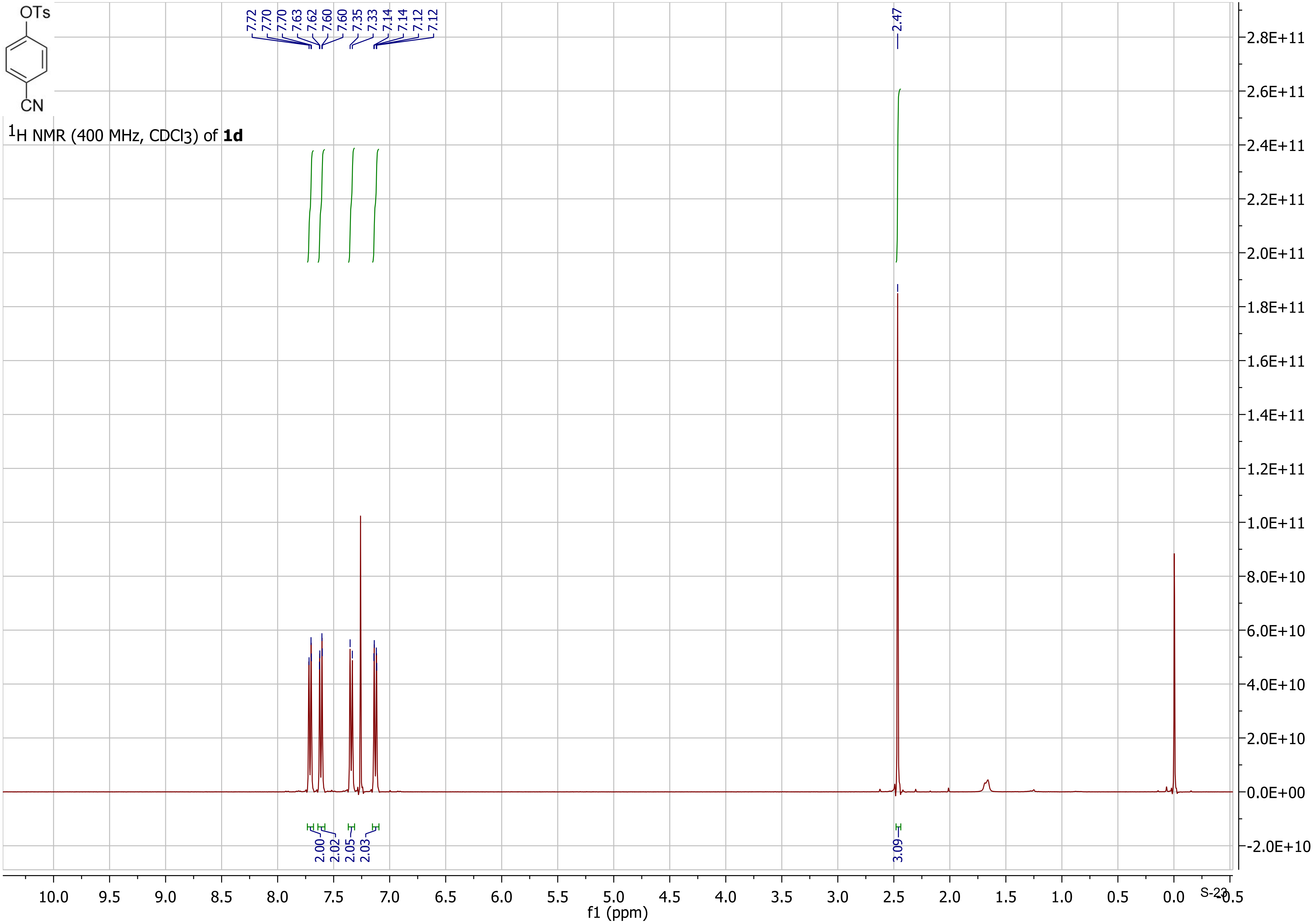
OTs
CN
1H NMR (400 MHz, CDCl3) of 1d
7.72 7.70 7.70 7.63 7.62 7.60 7.60 7.35 7.33 7.14 7.14 7.12 7.12
2.47
2.00 2.02 2.05 2.03
3.09
f1 (ppm)

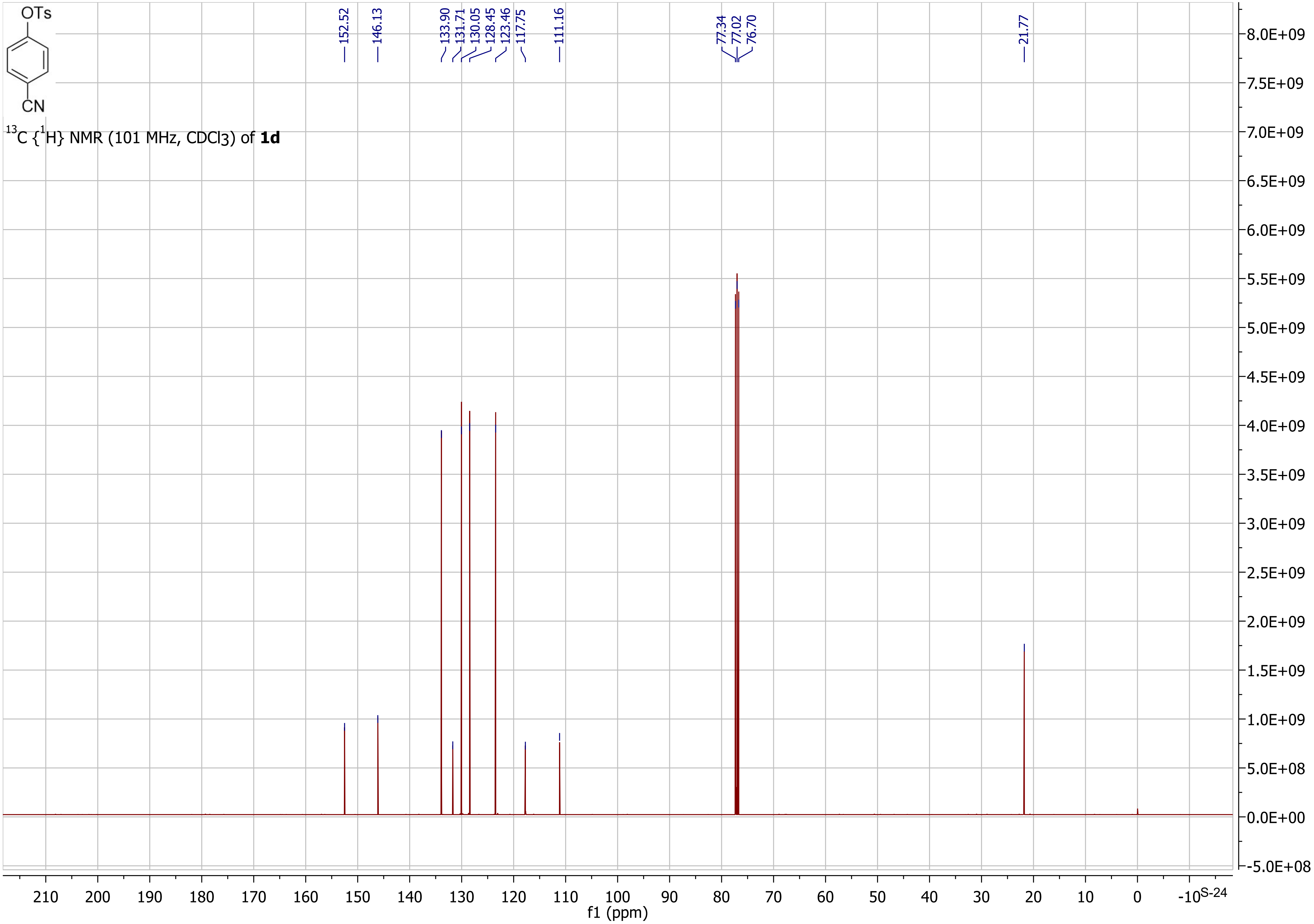
OTs
CN
13C {1H} NMR (101 MHz, CDCl3) of 1d
152.52
146.13
133.90
131.71
130.05
128.45
123.46
117.75
111.16
77.34
77.02
76.70
21.77
f1 (ppm)

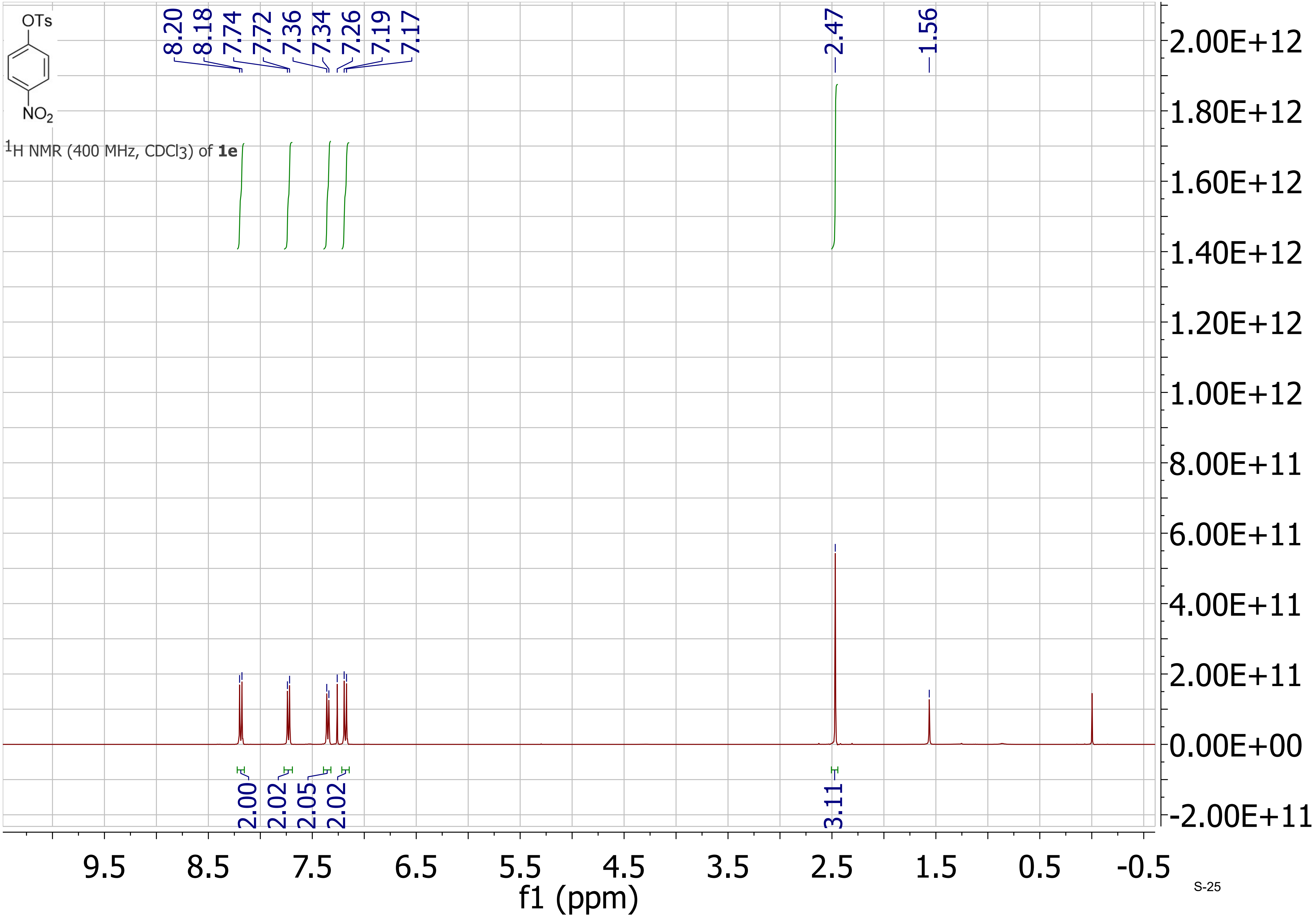
OTs
NO2
1H NMR (400 MHz, CDCl3) of 1e
8.20
8.18
7.74
7.72
7.36
7.34
7.26
7.19
7.17
2.47
1.56
2.00
2.02
2.05
2.02
3.11
f1 (ppm)

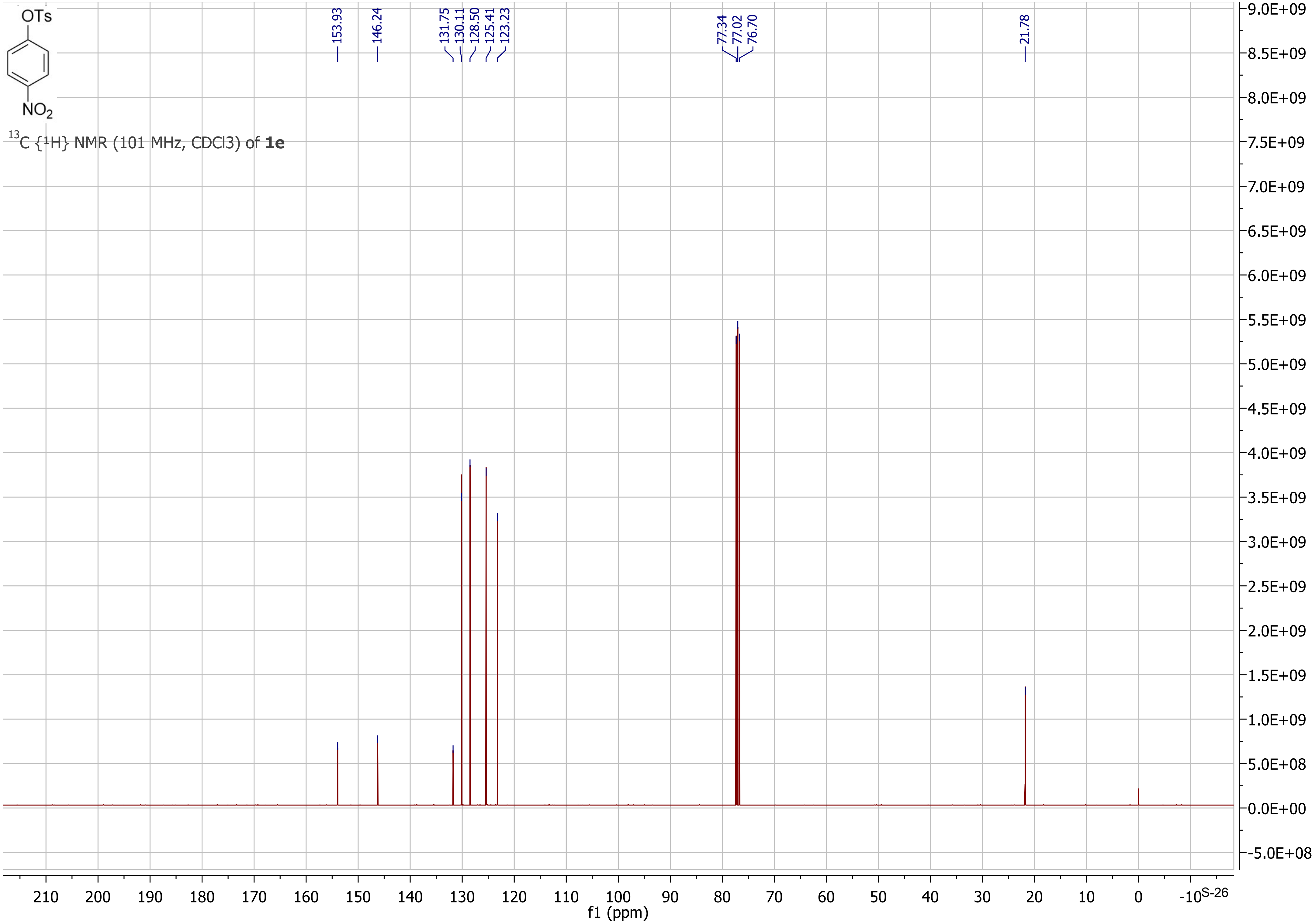
OTs
NO2
13C {1H} NMR (101 MHz, CDCl3) of 1e
153.93
146.24
131.75
130.11
128.50
125.41
123.23
77.34
77.02
76.70
21.78
9.0E+09
8.5E+09
8.0E+09
7.5E+09
7.0E+09
6.5E+09
6.0E+09
5.5E+09
5.0E+09
4.5E+09
4.0E+09
3.5E+09
3.0E+09
2.5E+09
2.0E+09
1.5E+09
1.0E+09
5.0E+08
0.0E+00
-5.0E+08
210 200 190 180 170 160 150 140 130 120 110 100 90 80 70 60 50 40 30 20 10 0 -10
f1 (ppm)

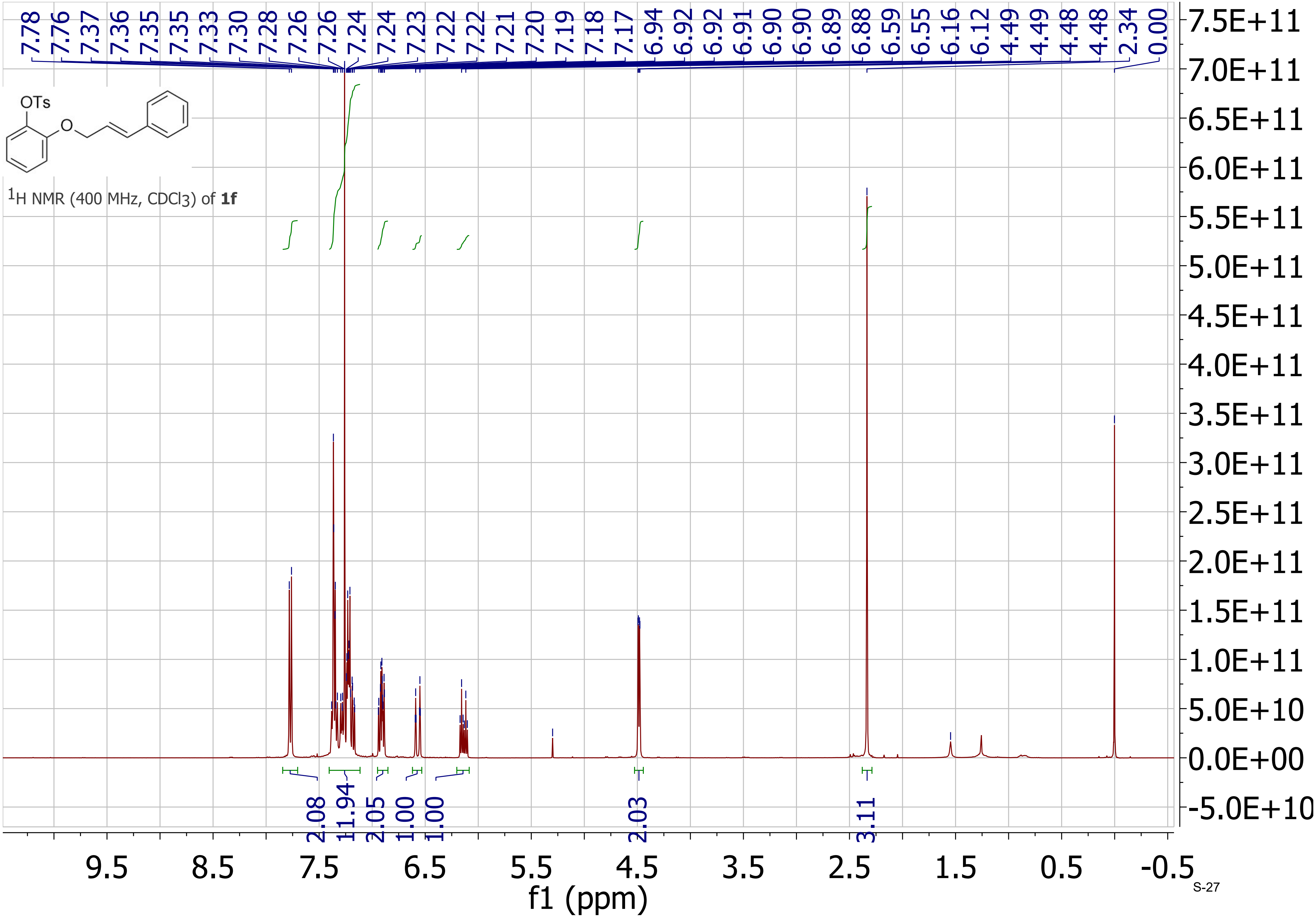

OTs
O
^{1}H NMR (400 MHz, CDCl$_3$) of **1f**
7.78 7.76 7.37 7.36 7.35 7.35 7.33 7.30 7.28 7.26 7.26 7.24 7.24 7.23 7.22 7.22 7.21 7.20 7.19 7.18 7.17 6.94 6.92 6.92 6.91 6.90 6.90 6.89 6.88 6.59 6.55 6.16 6.12 4.49 4.49 4.48 4.48 2.34 0.00
2.08 11.94 2.05 1.00 1.00 2.03 3.11
9.5 8.5 7.5 6.5 5.5 4.5 3.5 2.5 1.5 0.5 -0.5
f1 (ppm)
7.5E+11 7.0E+11 6.5E+11 6.0E+11 5.5E+11 5.0E+11 4.5E+11 4.0E+11 3.5E+11 3.0E+11 2.5E+11 2.0E+11 1.5E+11 1.0E+11 5.0E+10 0.0E+00 -5.0E+10

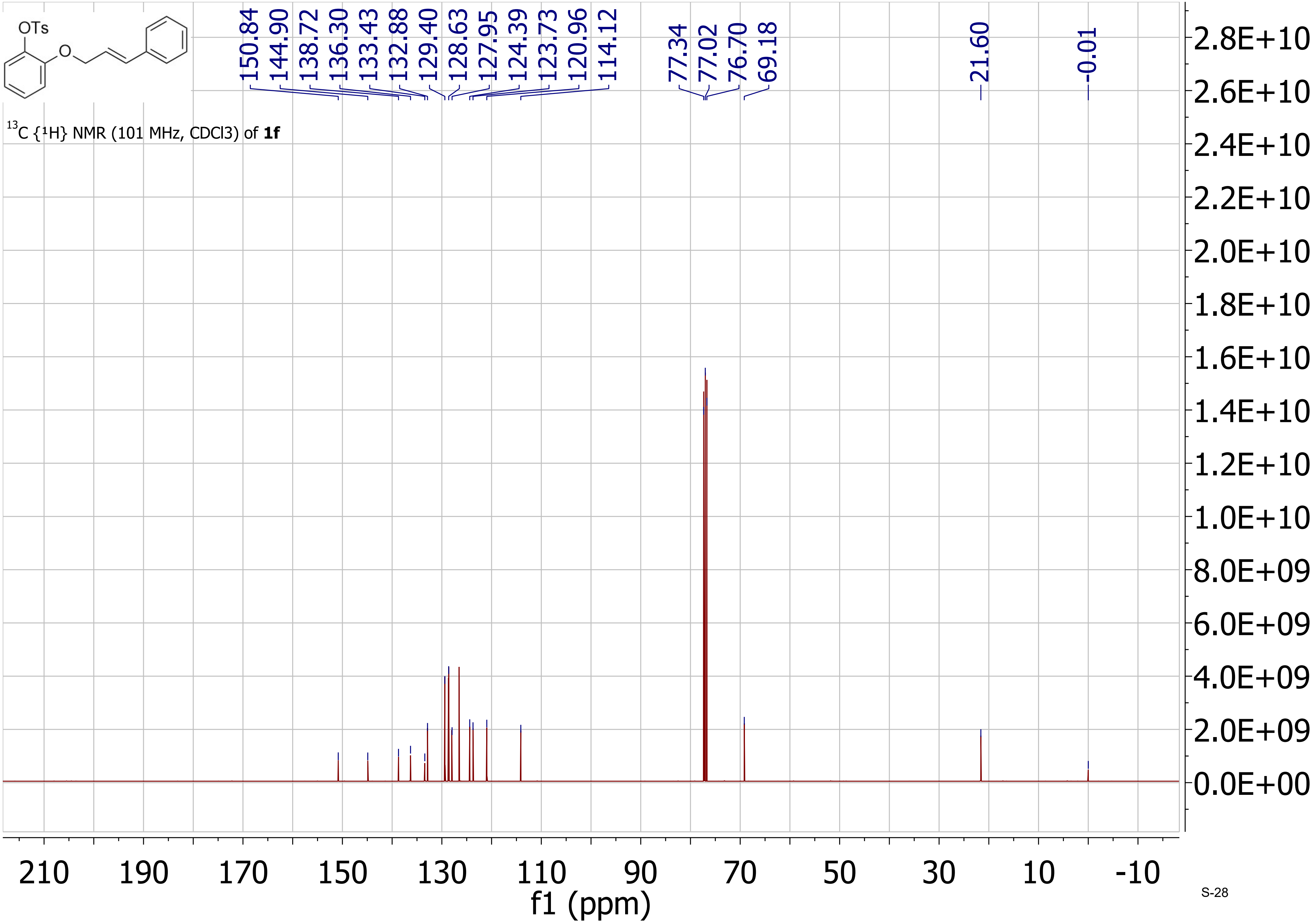

^{13}C {^{1}H} NMR (101 MHz, CDCl3) of **1f**

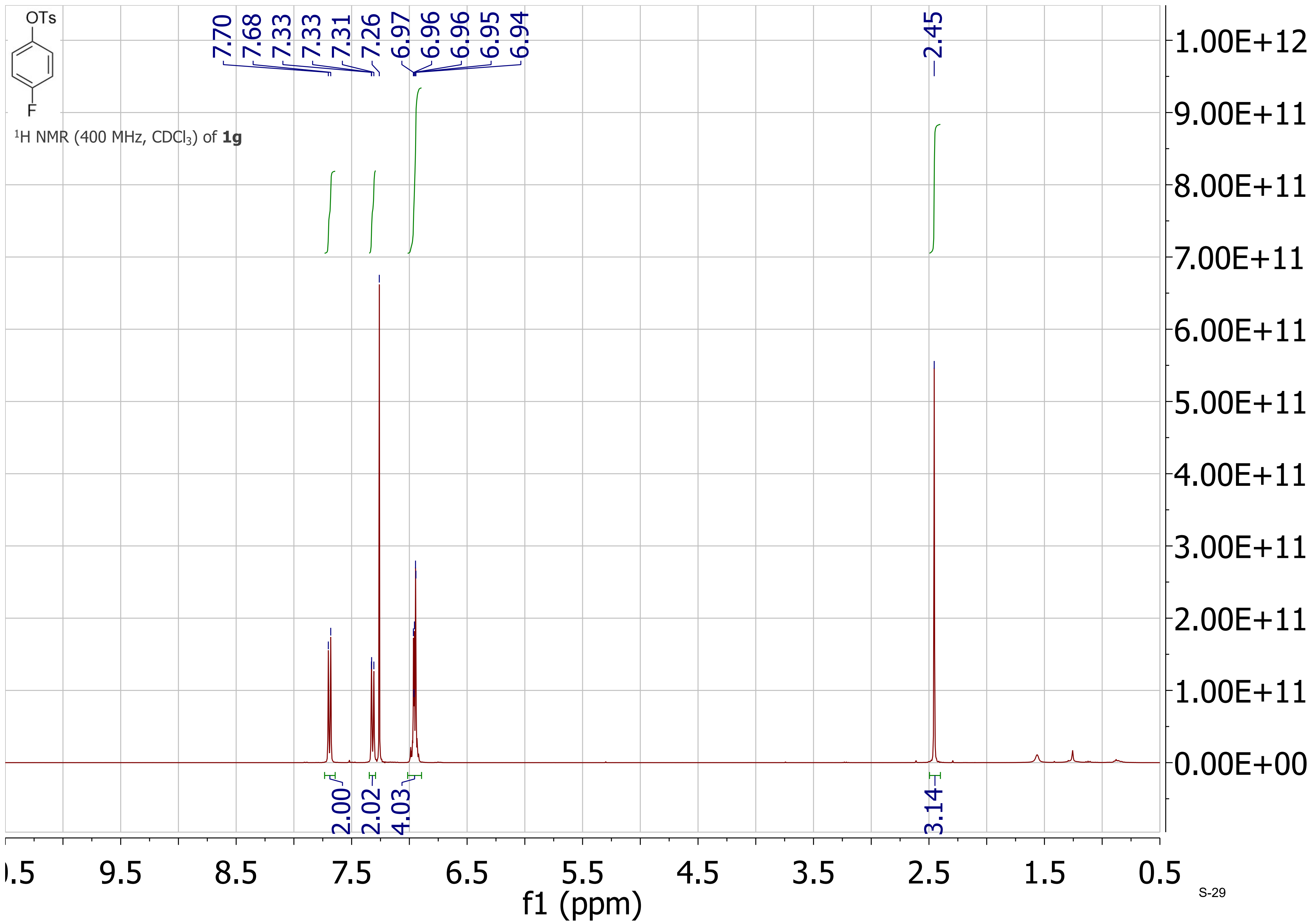
OTs
F
^{1}H NMR (400 MHz, CDCl3) of 1g
7.70 7.68 7.33 7.33 7.31 7.26 6.97 6.96 6.96 6.95 6.94
2.45
2.00 2.02 4.03
3.14
1.00E+12
9.00E+11
8.00E+11
7.00E+11
6.00E+11
5.00E+11
4.00E+11
3.00E+11
2.00E+11
1.00E+11
0.00E+00
9.5 8.5 7.5 6.5 5.5 4.5 3.5 2.5 1.5 0.5
f1 (ppm)

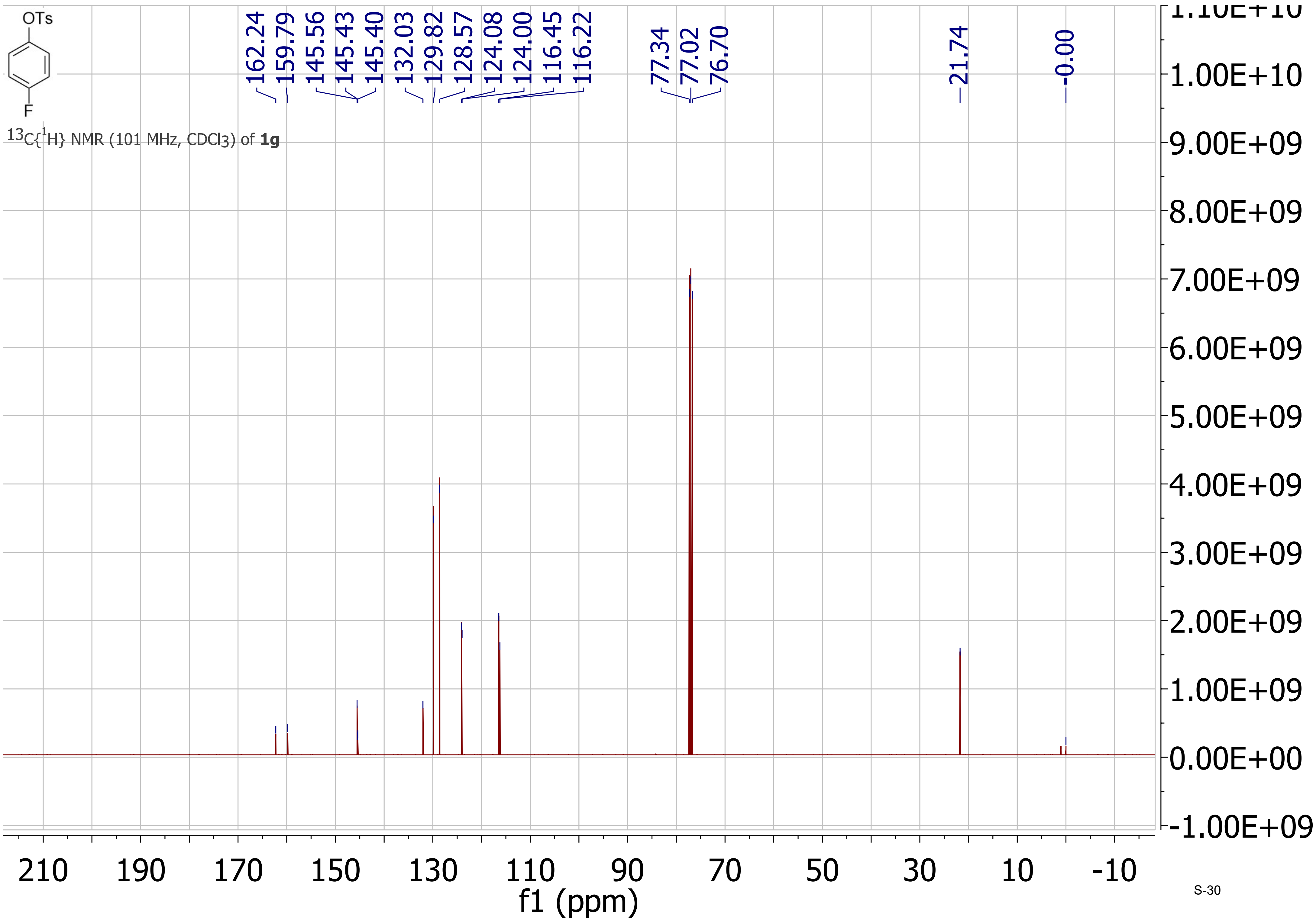
OTs
F
13C{1H} NMR (101 MHz, CDCl3) of 1g
162.24
159.79
145.56
145.43
145.40
132.03
129.82
128.57
124.08
124.00
116.45
116.22
77.34
77.02
76.70
21.74
-0.00
f1 (ppm)

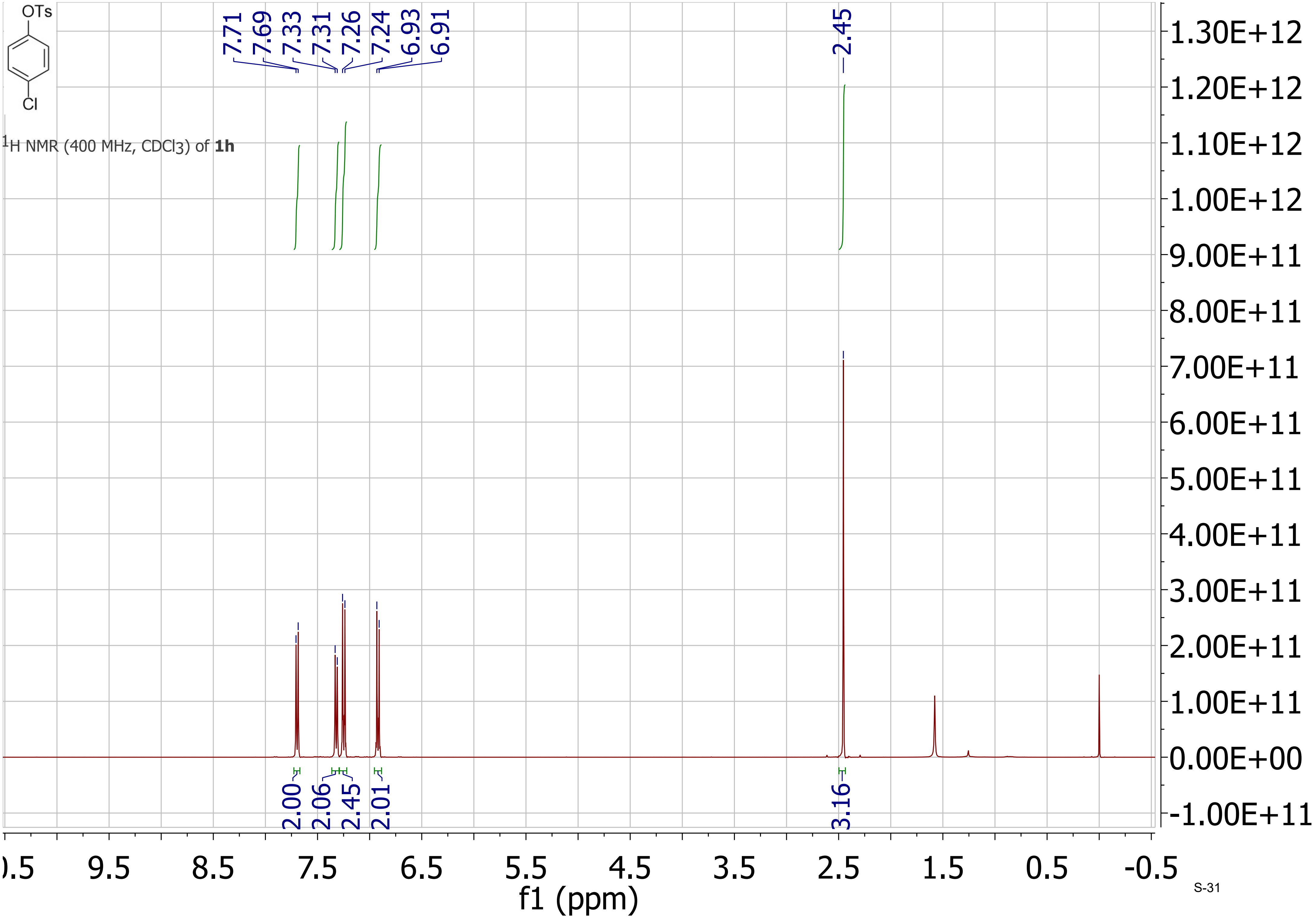
OTs
Cl
1H NMR (400 MHz, CDCl3) of **1h**
7.71
7.69
7.33
7.31
7.26
7.24
6.93
6.91
2.45
2.00
2.06
2.45
2.01
3.16
1.30E+12
1.20E+12
1.10E+12
1.00E+12
9.00E+11
8.00E+11
7.00E+11
6.00E+11
5.00E+11
4.00E+11
3.00E+11
2.00E+11
1.00E+11
0.00E+00
-1.00E+11
).5
9.5
8.5
7.5
6.5
5.5
4.5
3.5
2.5
1.5
0.5
-0.5
f1 (ppm)

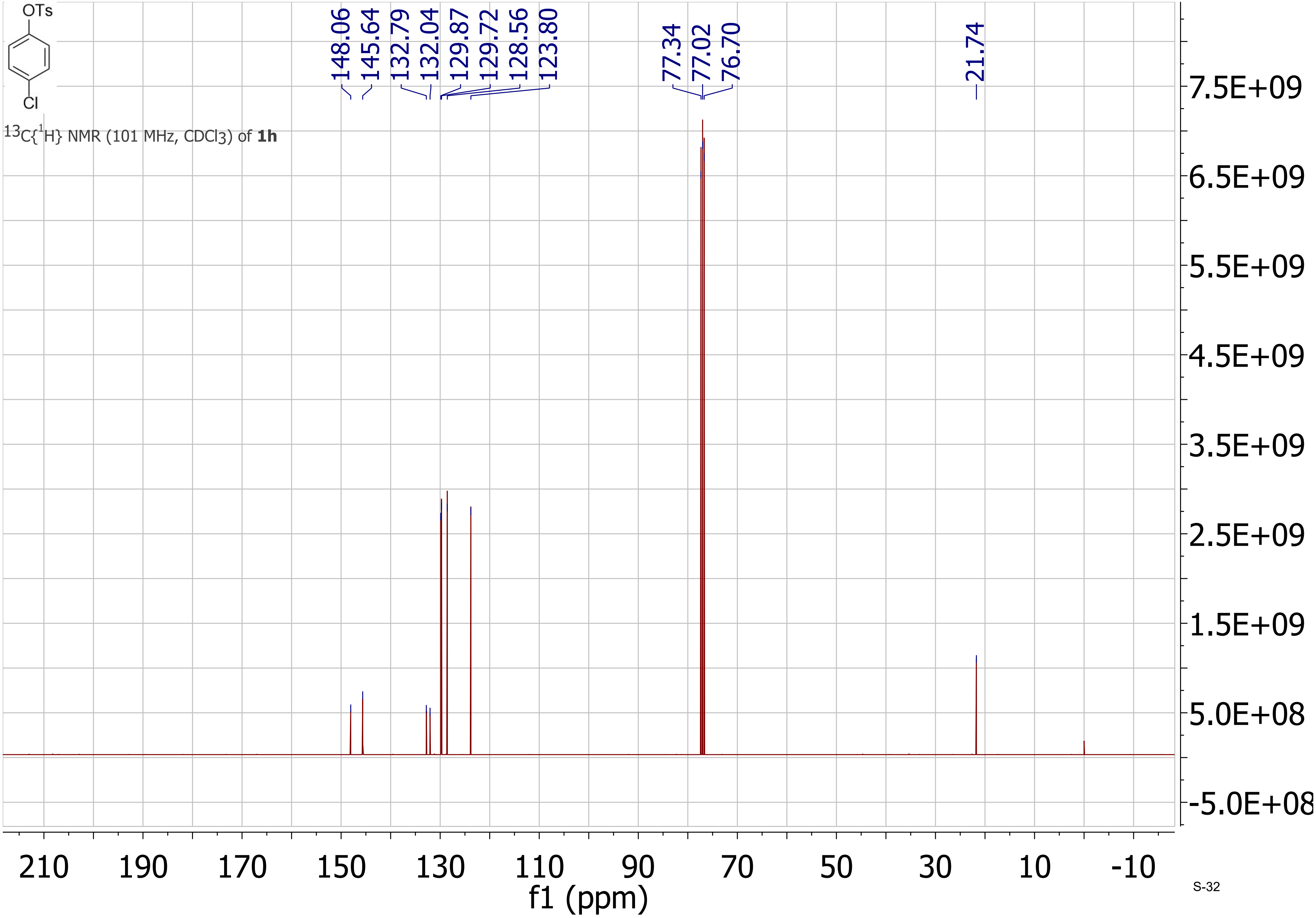

OTs
Cl
13C{1H} NMR (101 MHz, CDCl3) of 1h
148.06
145.64
132.79
132.04
129.87
129.72
128.56
123.80
77.34
77.02
76.70
21.74
7.5E+09
6.5E+09
5.5E+09
4.5E+09
3.5E+09
2.5E+09
1.5E+09
5.0E+08
-5.0E+08
210
190
170
150
130
110
90
70
50
30
10
-10
f1 (ppm)

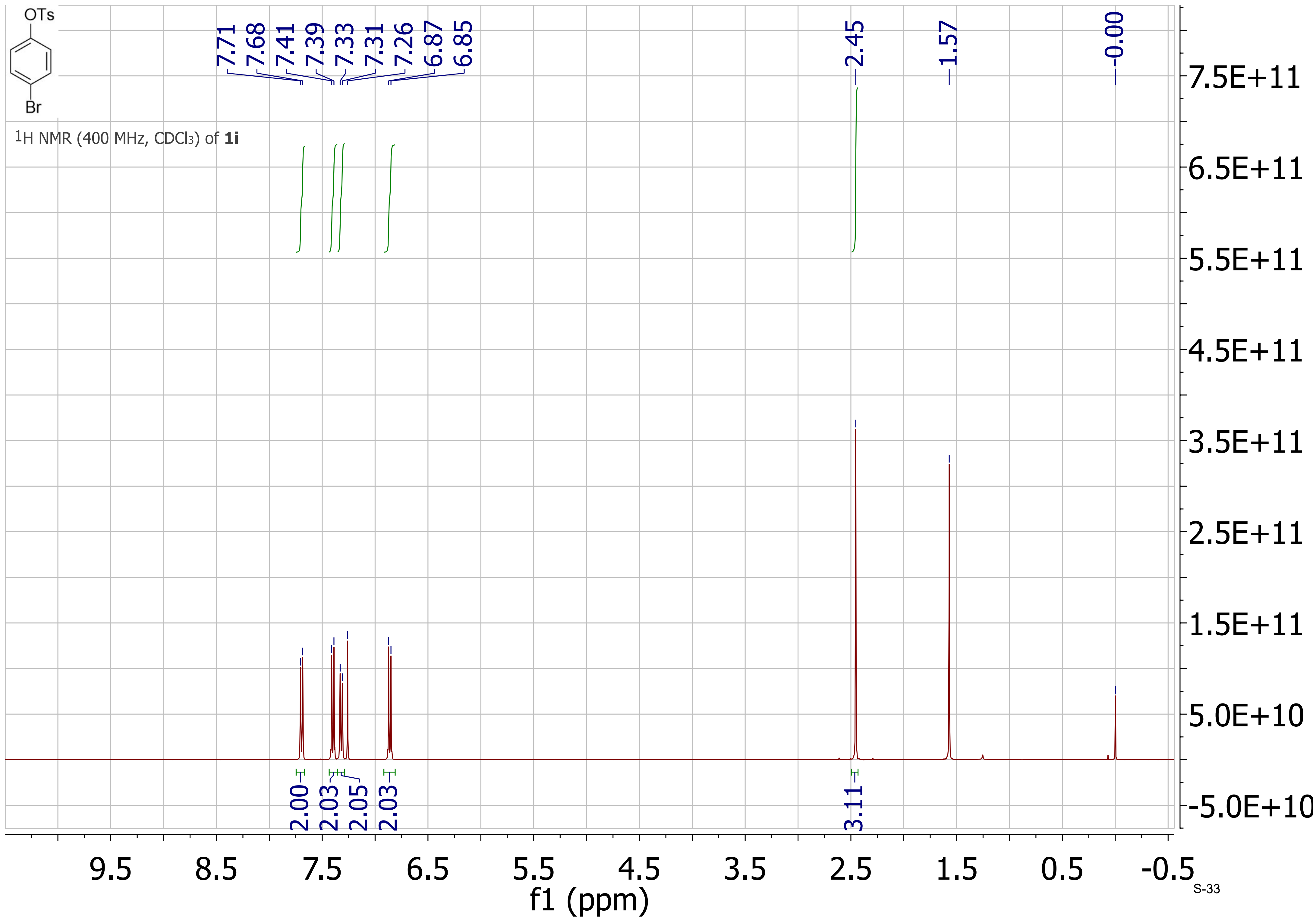

OTs
Br
1H NMR (400 MHz, CDCl3) of **1i**
7.71
7.68
7.41
7.39
7.33
7.31
7.26
6.87
6.85
2.45
1.57
-0.00
2.00
2.03
2.05
2.03
3.11
7.5E+11
6.5E+11
5.5E+11
4.5E+11
3.5E+11
2.5E+11
1.5E+11
5.0E+10
-5.0E+10
9.5
8.5
7.5
6.5
5.5
4.5
3.5
2.5
1.5
0.5
-0.5
f1 (ppm)

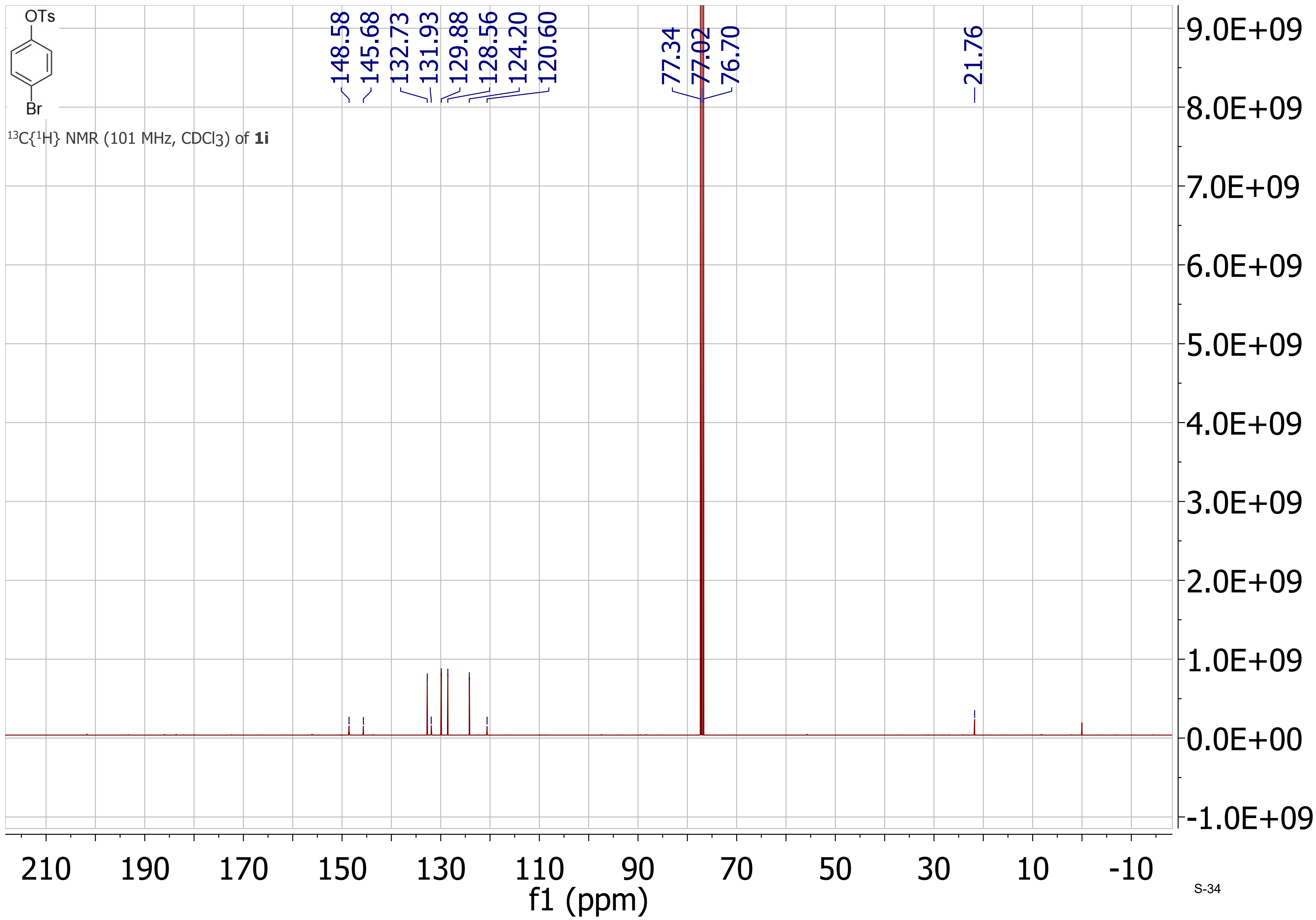
OTs
Br
$^{13}C\{^1H\}$ NMR (101 MHz, $CDCl_3$) of **1i**
148.58
145.68
132.73
131.93
129.88
128.56
124.20
120.60
77.34
77.02
76.70
21.76
9.0E+09
8.0E+09
7.0E+09
6.0E+09
5.0E+09
4.0E+09
3.0E+09
2.0E+09
1.0E+09
0.0E+00
-1.0E+09
210
190
170
150
130
110
90
70
50
30
10
-10
f1 (ppm)

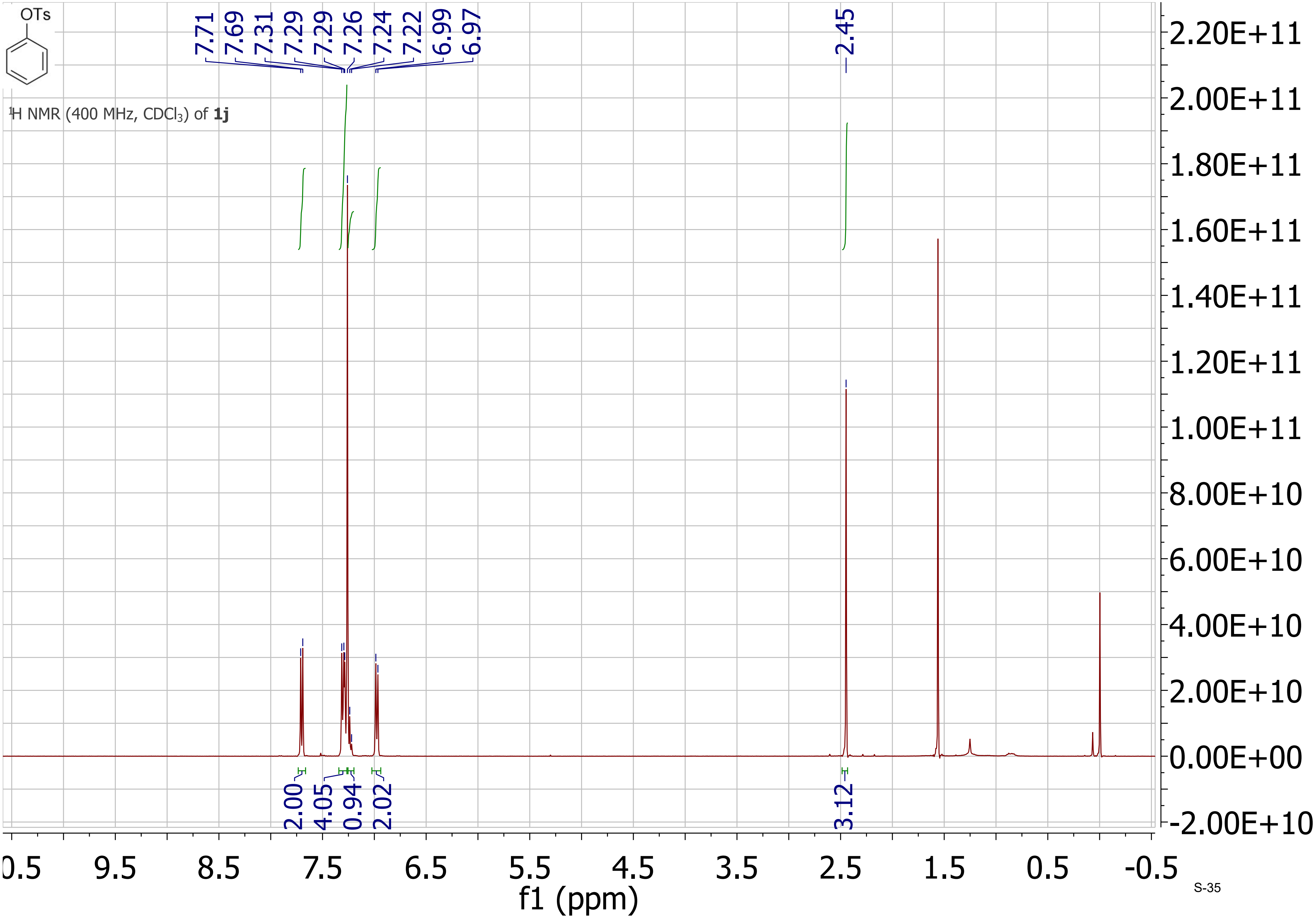
OTs
¹H NMR (400 MHz, CDCl₃) of 1j
7.71
7.69
7.31
7.29
7.29
7.26
7.24
7.22
6.99
6.97
2.45
2.00
4.05
0.94
2.02
3.12
2.20E+11
2.00E+11
1.80E+11
1.60E+11
1.40E+11
1.20E+11
1.00E+11
8.00E+10
6.00E+10
4.00E+10
2.00E+10
0.00E+00
-2.00E+10
0.5
9.5
8.5
7.5
6.5
5.5
4.5
3.5
2.5
1.5
0.5
-0.5
f1 (ppm)

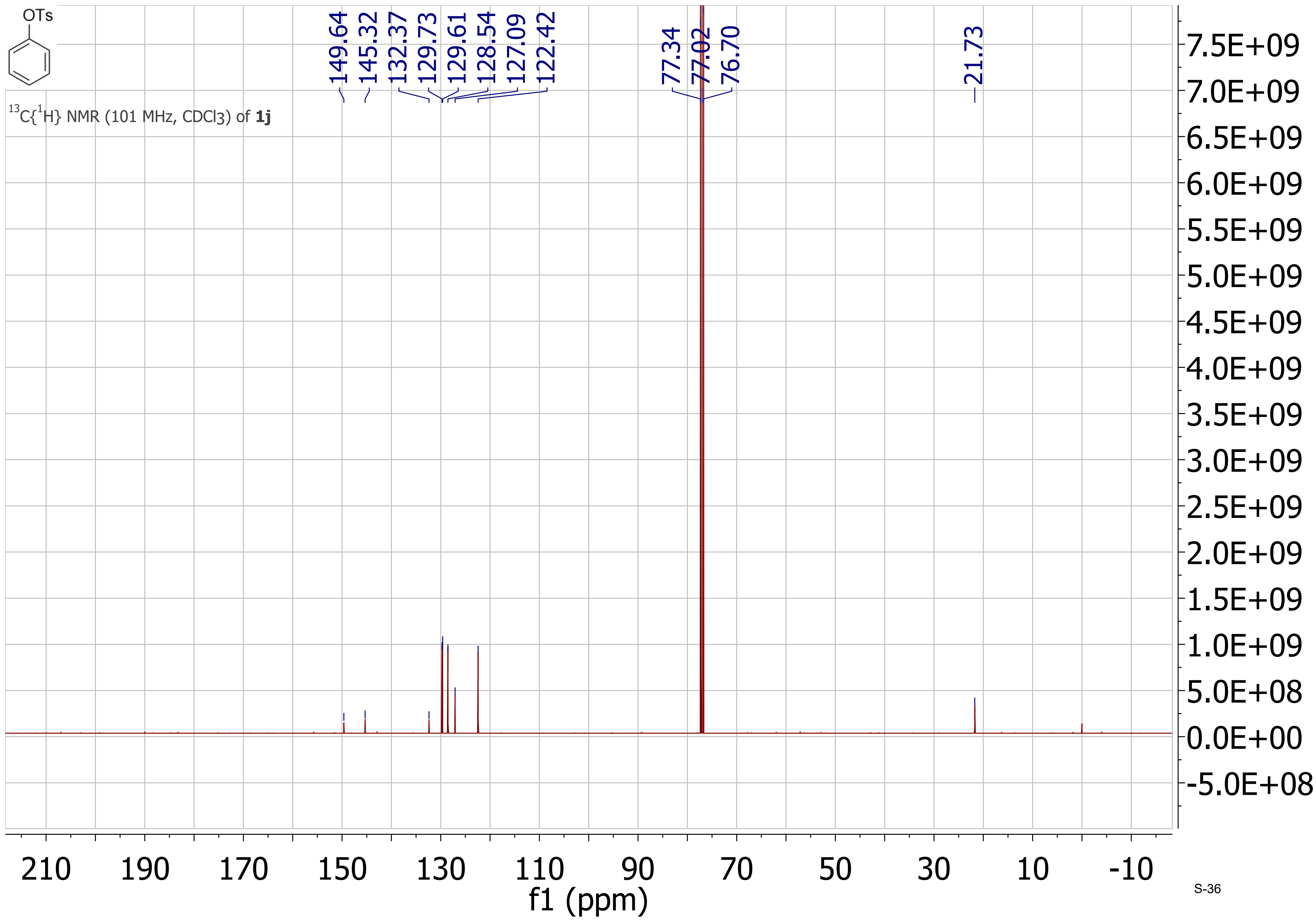
OTs
$^{13}C\{^{1}H\}$ NMR (101 MHz, CDCl3) of **1j**
149.64
145.32
132.37
129.73
129.61
128.54
127.09
122.42
77.34
77.02
76.70
21.73
7.5E+09
7.0E+09
6.5E+09
6.0E+09
5.5E+09
5.0E+09
4.5E+09
4.0E+09
3.5E+09
3.0E+09
2.5E+09
2.0E+09
1.5E+09
1.0E+09
5.0E+08
0.0E+00
-5.0E+08
210
190
170
150
130
110
90
70
50
30
10
-10
f1 (ppm)

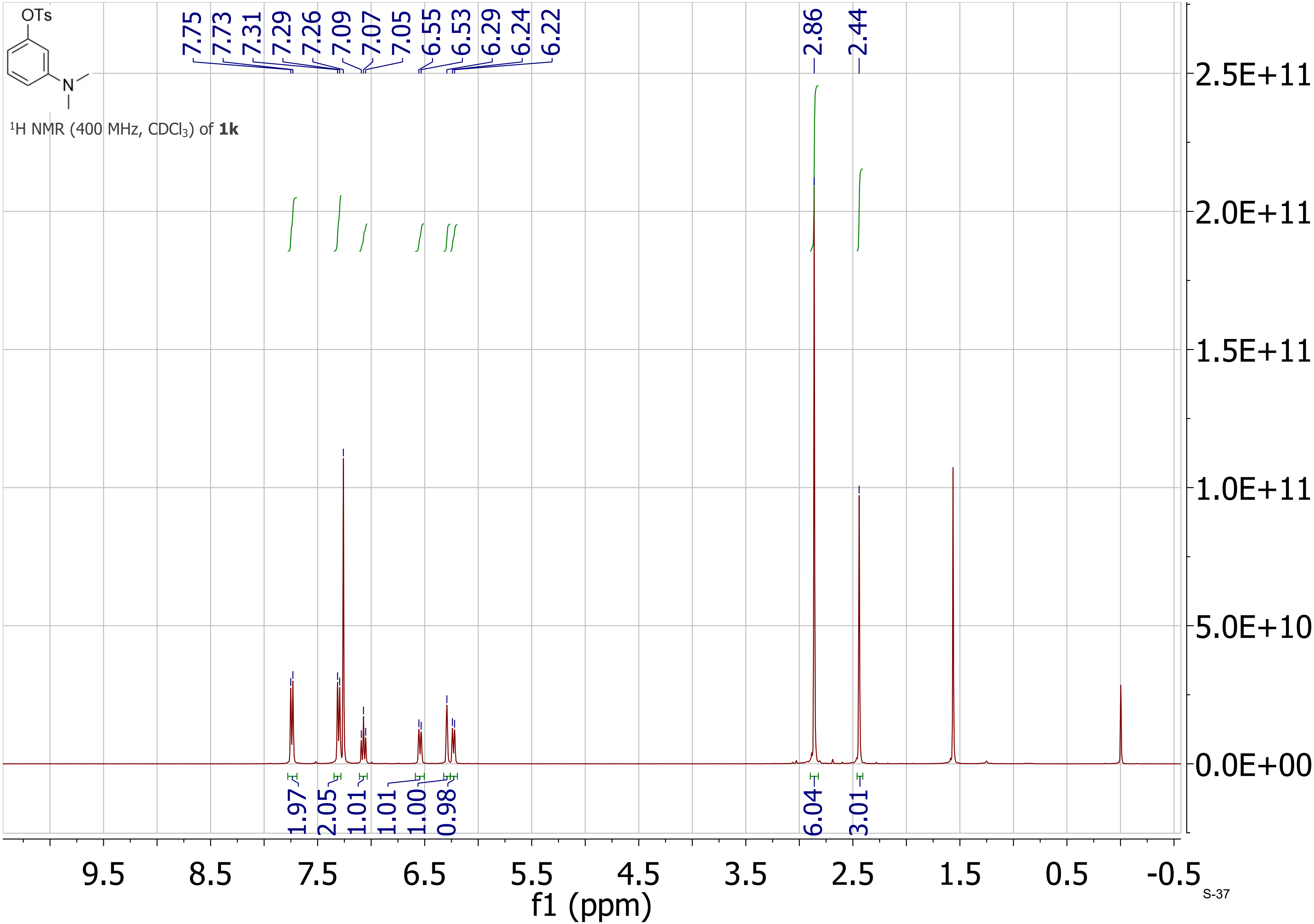

OTs
N
¹H NMR (400 MHz, CDCl3) of 1k
7.75
7.73
7.31
7.29
7.26
7.09
7.07
7.05
6.55
6.53
6.29
6.24
6.22
2.86
2.44
1.97
2.05
1.01
1.01
1.00
0.98
6.04
3.01
2.5E+11
2.0E+11
1.5E+11
1.0E+11
5.0E+10
0.0E+00
9.5
8.5
7.5
6.5
5.5
4.5
3.5
2.5
1.5
0.5
-0.5
f1 (ppm)

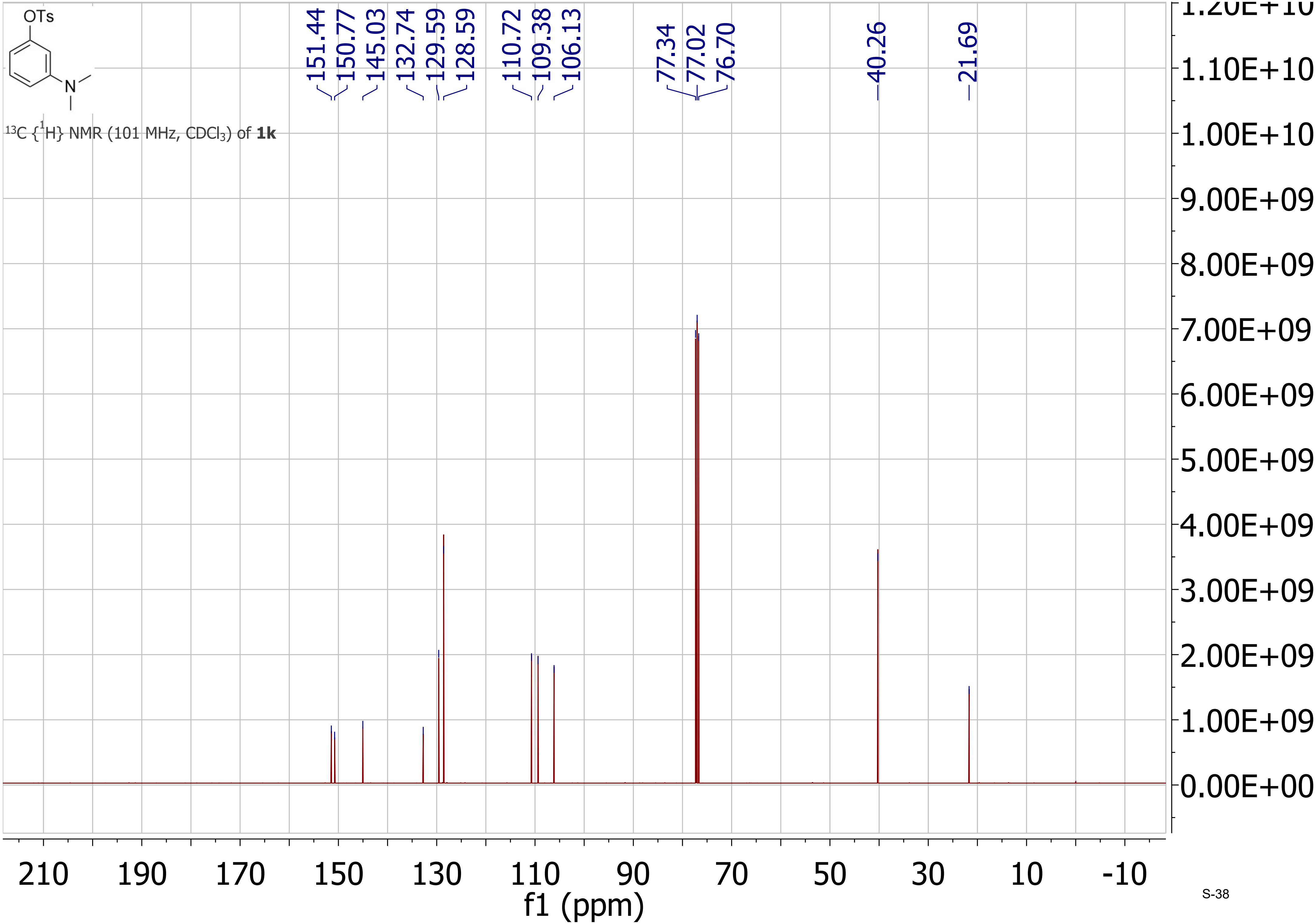
OTs
N
13C {1H} NMR (101 MHz, CDCl3) of 1k
151.44
150.77
145.03
132.74
129.59
128.59
110.72
109.38
106.13
77.34
77.02
76.70
40.26
21.69
f1 (ppm)

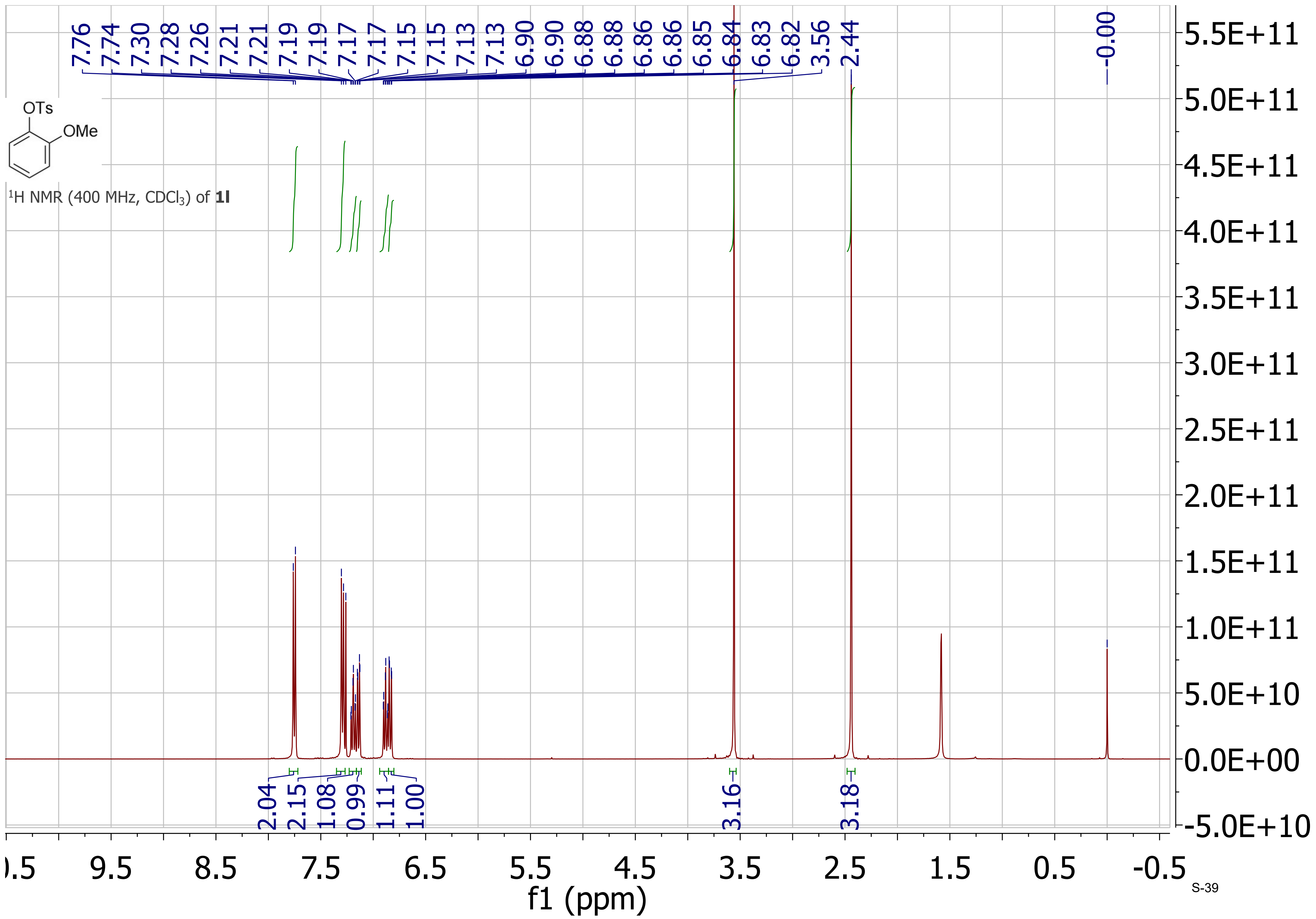
7.76
7.74
7.30
7.28
7.26
7.21
7.21
7.19
7.19
7.17
7.17
7.15
7.15
7.13
7.13
6.90
6.90
6.88
6.88
6.86
6.86
6.85
6.84
6.83
6.82
3.56
2.44
-0.00
OTs
OMe
^{1}H NMR (400 MHz, $CDCl_3$) of **1l**
2.04
2.15
1.08
0.99
1.11
1.00
3.16
3.18
5.5E+11
5.0E+11
4.5E+11
4.0E+11
3.5E+11
3.0E+11
2.5E+11
2.0E+11
1.5E+11
1.0E+11
5.0E+10
0.0E+00
-5.0E+10
9.5
8.5
7.5
6.5
5.5
4.5
3.5
2.5
1.5
0.5
-0.5
f1 (ppm)

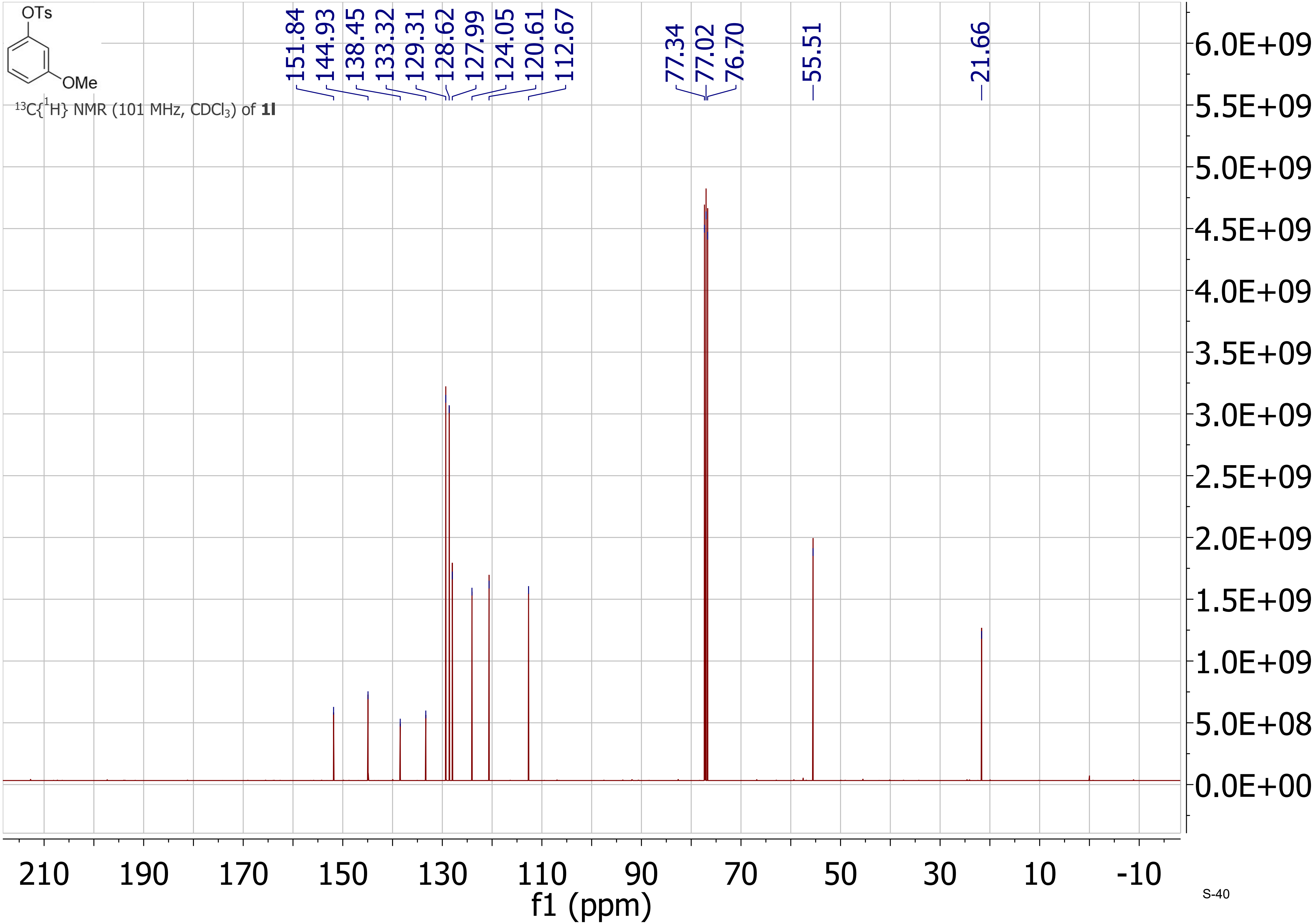
OTs
OMe
$^{13}C\{^1H\}$ NMR (101 MHz, $CDCl_3$) of **1l**
151.84
144.93
138.45
133.32
129.31
128.62
127.99
124.05
120.61
112.67
77.34
77.02
76.70
55.51
21.66
6.0E+09
5.5E+09
5.0E+09
4.5E+09
4.0E+09
3.5E+09
3.0E+09
2.5E+09
2.0E+09
1.5E+09
1.0E+09
5.0E+08
0.0E+00
210
190
170
150
130
110
90
70
50
30
10
-10
f1 (ppm)

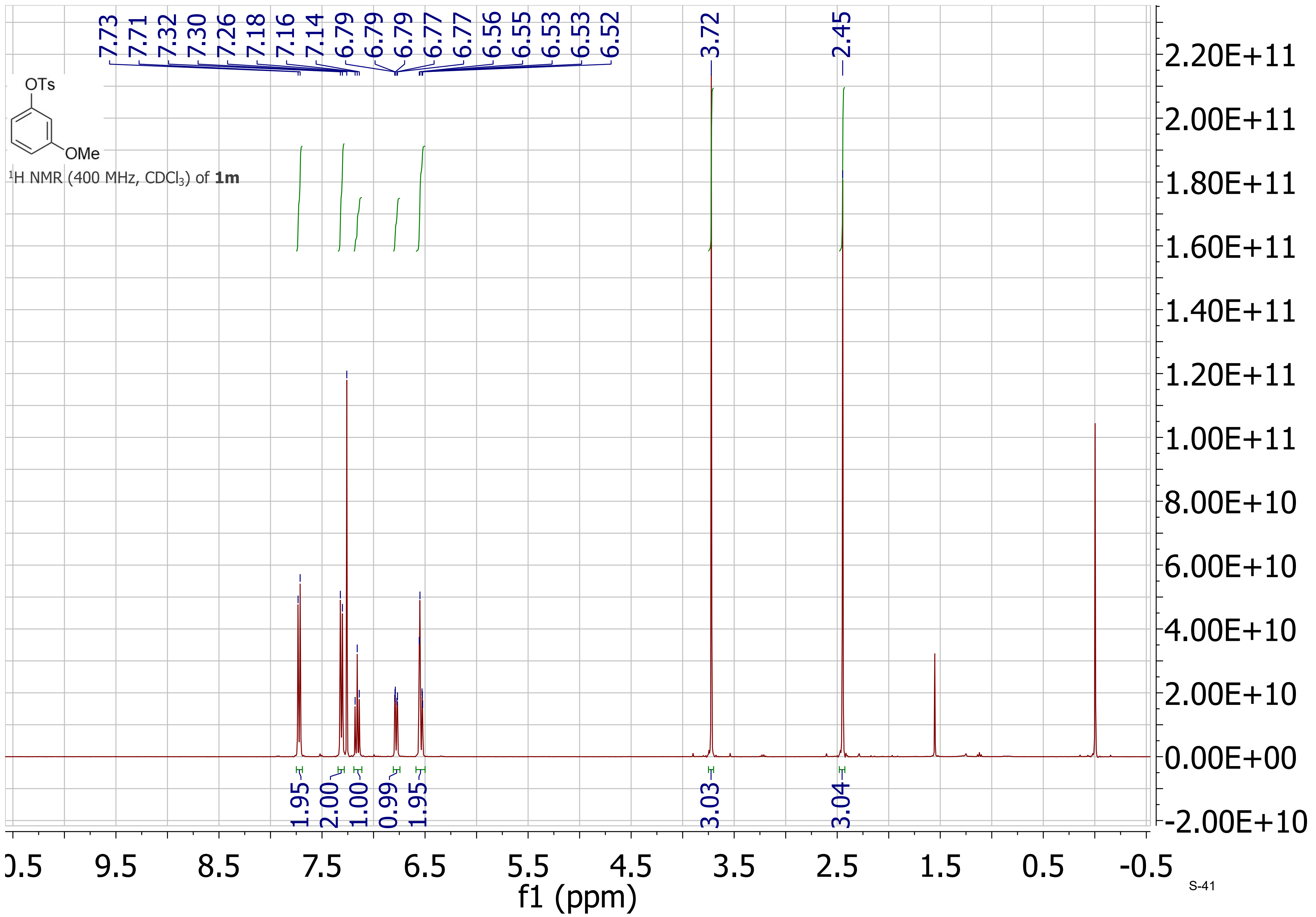

OTs
OMe
^{1}H NMR (400 MHz, CDCl$_3$) of **1m**
7.73
7.71
7.32
7.30
7.26
7.18
7.16
7.14
6.79
6.79
6.79
6.77
6.77
6.56
6.55
6.53
6.53
6.52
3.72
2.45
1.95
2.00
1.00
0.99
1.95
3.03
3.04
2.20E+11
2.00E+11
1.80E+11
1.60E+11
1.40E+11
1.20E+11
1.00E+11
8.00E+10
6.00E+10
4.00E+10
2.00E+10
0.00E+00
-2.00E+10
9.5
8.5
7.5
6.5
5.5
4.5
3.5
2.5
1.5
0.5
-0.5
f1 (ppm)

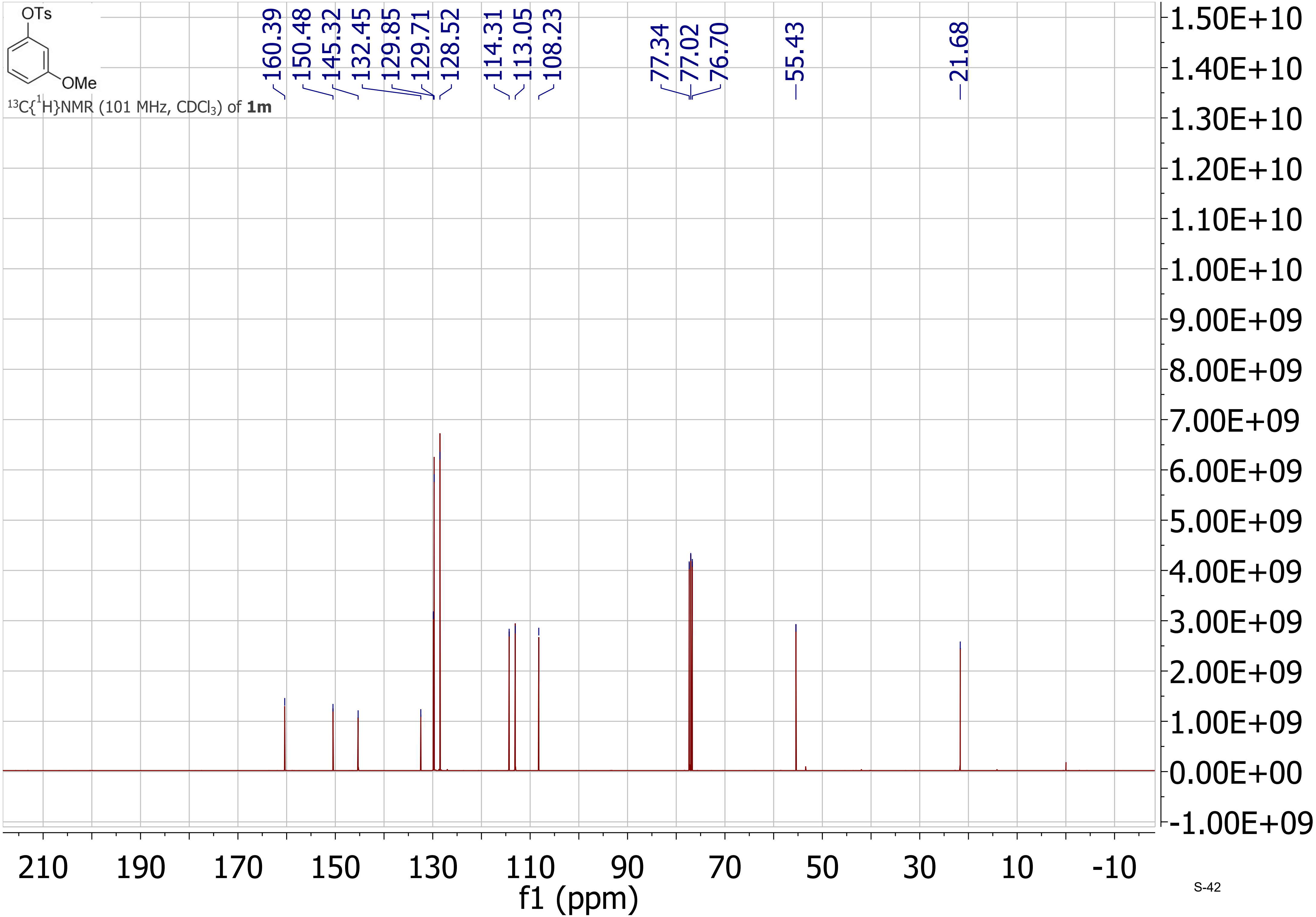
OTs
OMe
$^{13}C\{^{1}H\}$NMR (101 MHz, $CDCl_3$) of **1m**
160.39
150.48
145.32
132.45
129.85
129.71
128.52
114.31
113.05
108.23
77.34
77.02
76.70
55.43
21.68
1.50E+10
1.40E+10
1.30E+10
1.20E+10
1.10E+10
1.00E+10
9.00E+09
8.00E+09
7.00E+09
6.00E+09
5.00E+09
4.00E+09
3.00E+09
2.00E+09
1.00E+09
0.00E+00
-1.00E+09
210 190 170 150 130 110 90 70 50 30 10 -10
f1 (ppm)

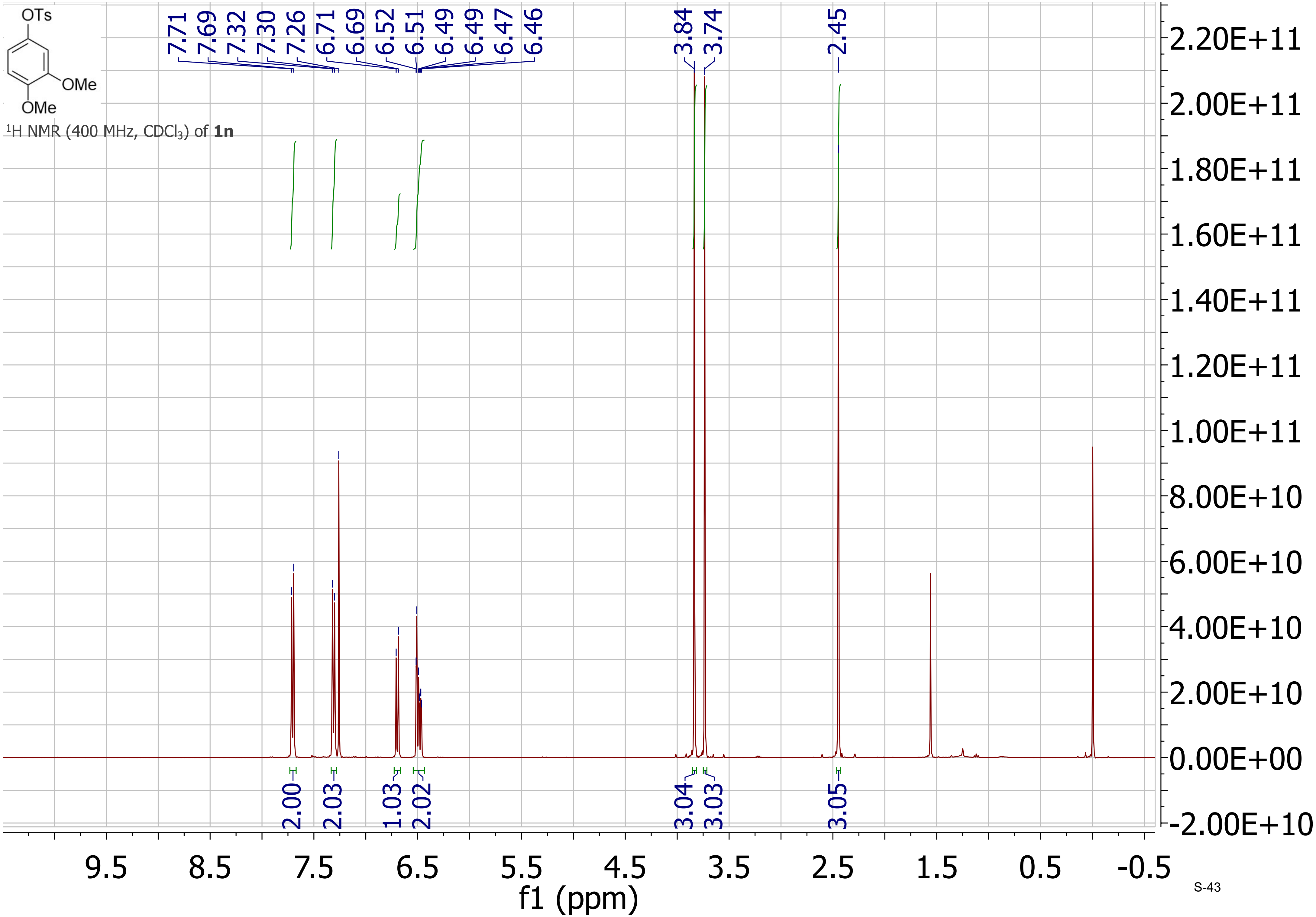
OTs
OMe
OMe
^{1}H NMR (400 MHz, CDCl3) of 1n
7.71
7.69
7.32
7.30
7.26
6.71
6.69
6.52
6.51
6.49
6.49
6.47
6.46
3.84
3.74
2.45
2.00
2.03
1.03
2.02
3.04
3.03
3.05
2.20E+11
2.00E+11
1.80E+11
1.60E+11
1.40E+11
1.20E+11
1.00E+11
8.00E+10
6.00E+10
4.00E+10
2.00E+10
0.00E+00
-2.00E+10
9.5
8.5
7.5
6.5
5.5
4.5
3.5
2.5
1.5
0.5
-0.5
f1 (ppm)

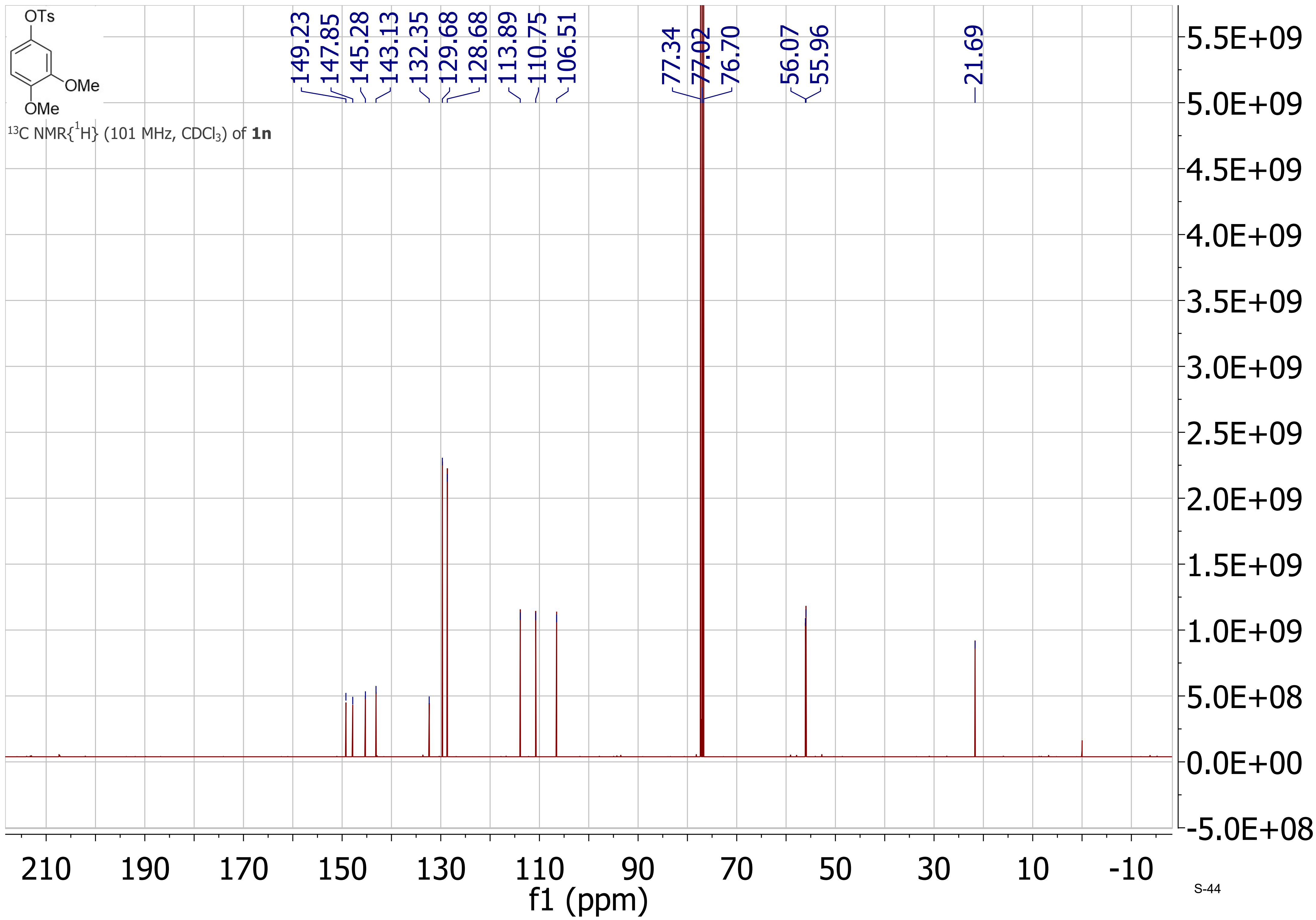

OTs
OMe
OMe
^{13}C NMR{^{1}H} (101 MHz, CDCl$_3$) of **1n**
149.23
147.85
145.28
143.13
132.35
129.68
128.68
113.89
110.75
106.51
77.34
77.02
76.70
56.07
55.96
21.69
5.5E+09
5.0E+09
4.5E+09
4.0E+09
3.5E+09
3.0E+09
2.5E+09
2.0E+09
1.5E+09
1.0E+09
5.0E+08
0.0E+00
-5.0E+08
210
190
170
150
130
110
90
70
50
30
10
-10
f1 (ppm)

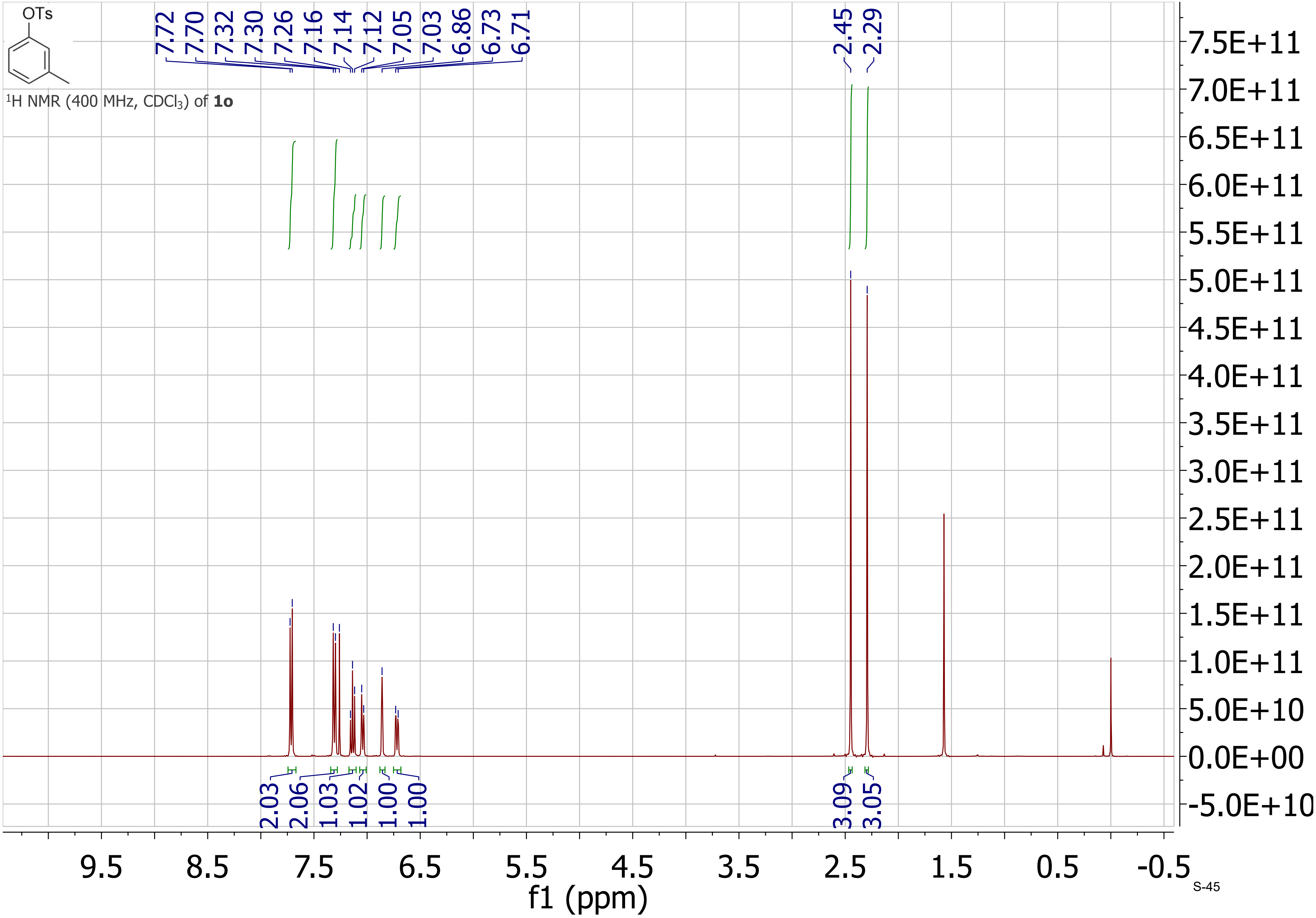

OTs
¹H NMR (400 MHz, CDCl3) of 1o
7.72
7.70
7.32
7.30
7.26
7.16
7.14
7.12
7.05
7.03
6.86
6.73
6.71
2.45
2.29
2.03
2.06
1.03
1.02
1.00
1.00
3.09
3.05
f1 (ppm)

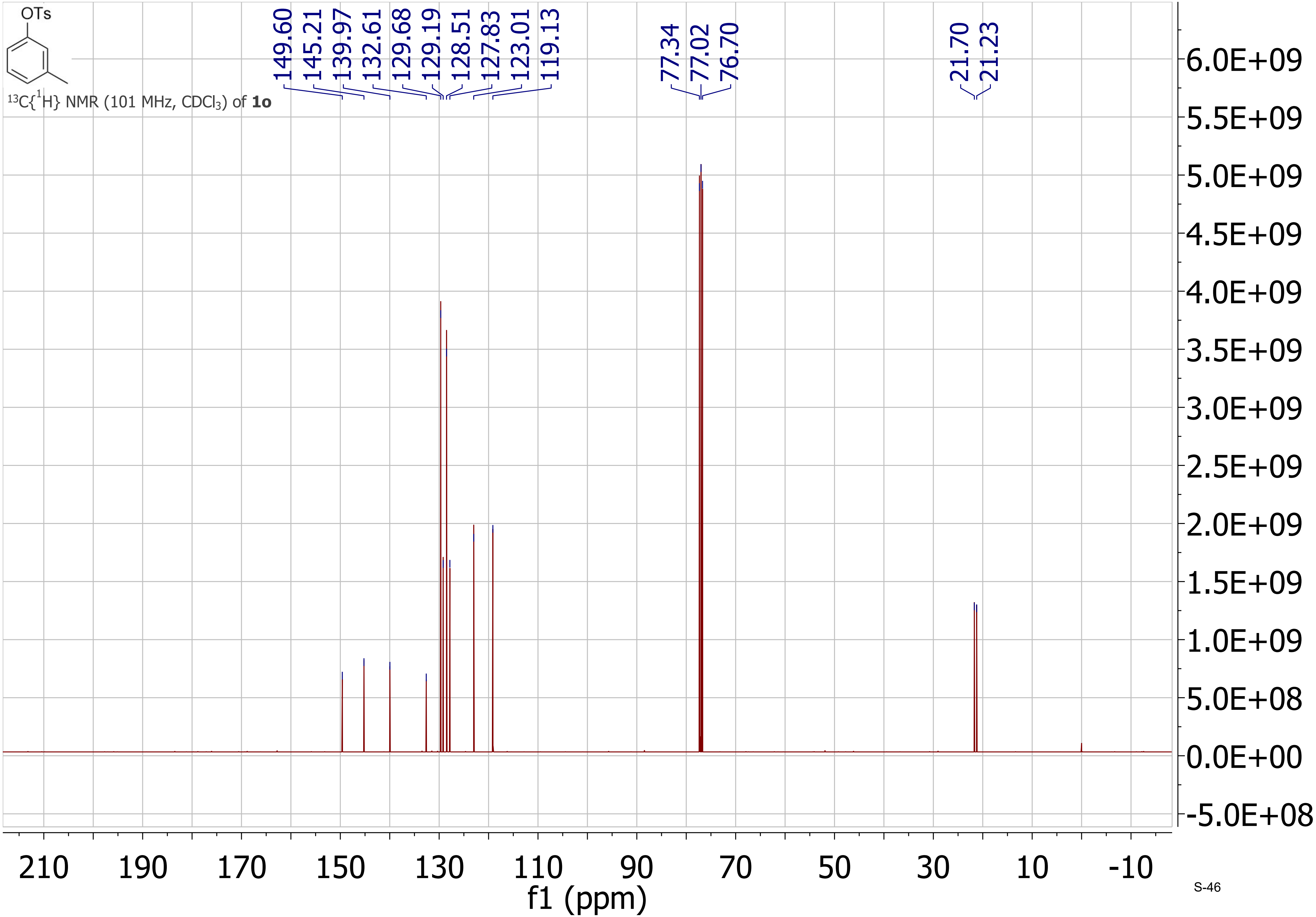

$^{13}C\{^{1}H\}$ NMR (101 MHz, $CDCl_3$) of **1o**

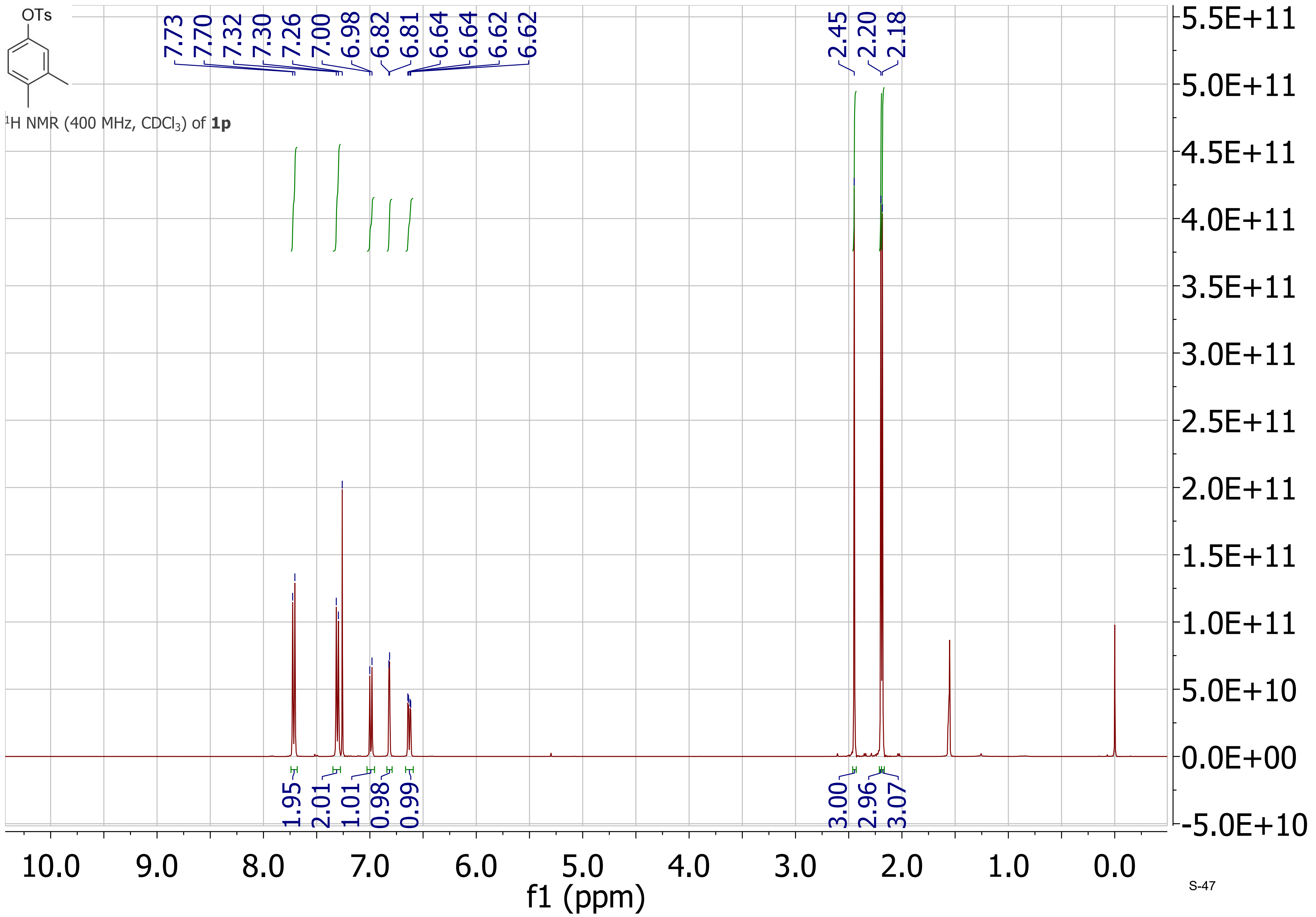

OTs
¹H NMR (400 MHz, CDCl3) of **1p**
7.73
7.70
7.32
7.30
7.26
7.00
6.98
6.82
6.81
6.64
6.64
6.62
6.62
2.45
2.20
2.18
1.95
2.01
1.01
0.98
0.99
3.00
2.96
3.07
10.0
9.0
8.0
7.0
6.0
5.0
4.0
3.0
2.0
1.0
0.0
f1 (ppm)
5.5E+11
5.0E+11
4.5E+11
4.0E+11
3.5E+11
3.0E+11
2.5E+11
2.0E+11
1.5E+11
1.0E+11
5.0E+10
0.0E+00
-5.0E+10

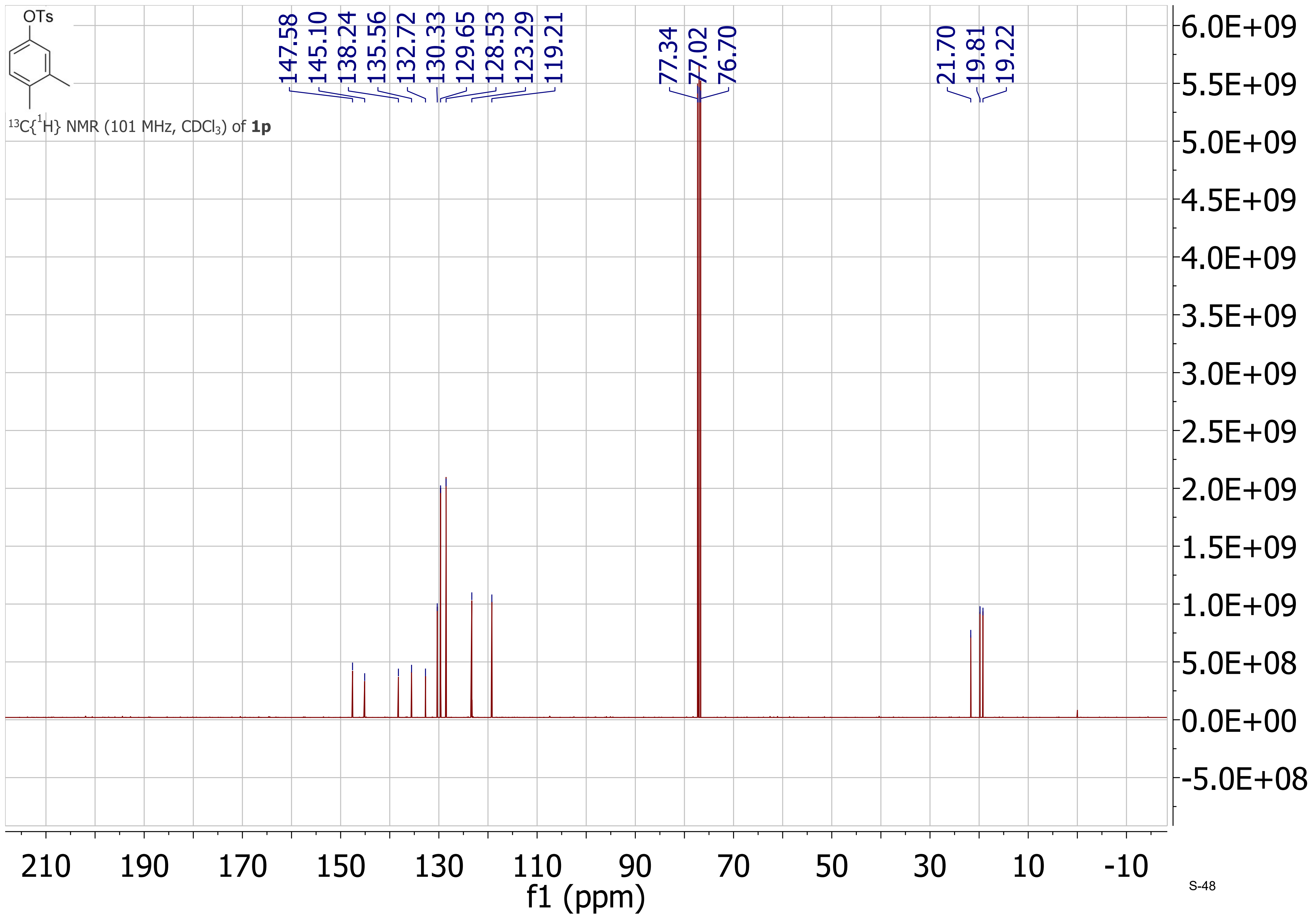

OTs
13C{1H} NMR (101 MHz, CDCl3) of 1p
147.58
145.10
138.24
135.56
132.72
130.33
129.65
128.53
123.29
119.21
77.34
77.02
76.70
21.70
19.81
19.22
f1 (ppm)

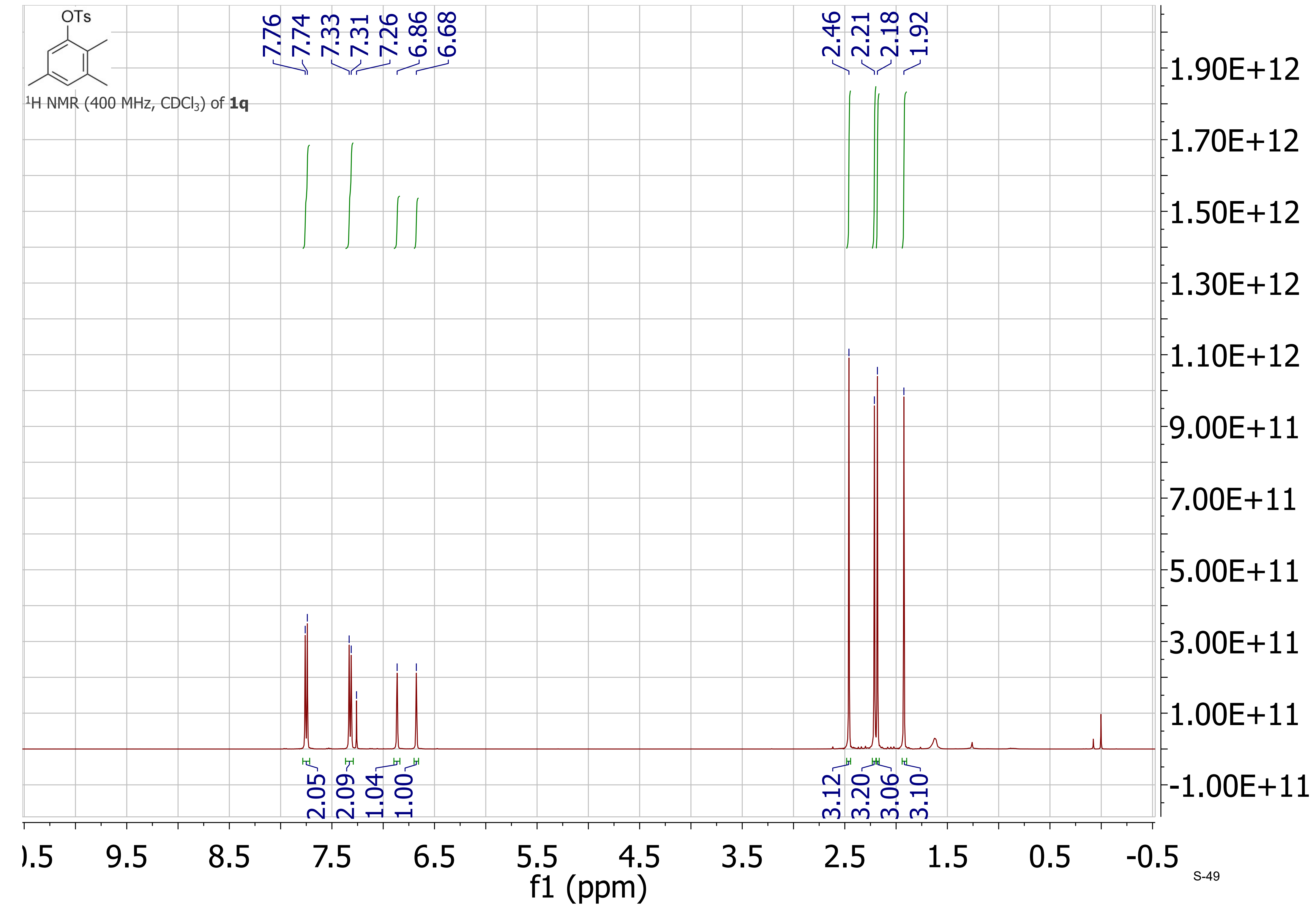
OTs
^{1}H NMR (400 MHz, $CDCl_3$) of **1q**
7.76
7.74
7.33
7.31
7.26
6.86
6.68
2.46
2.21
2.18
1.92
2.05
2.09
1.04
1.00
3.12
3.20
3.06
3.10
f1 (ppm)

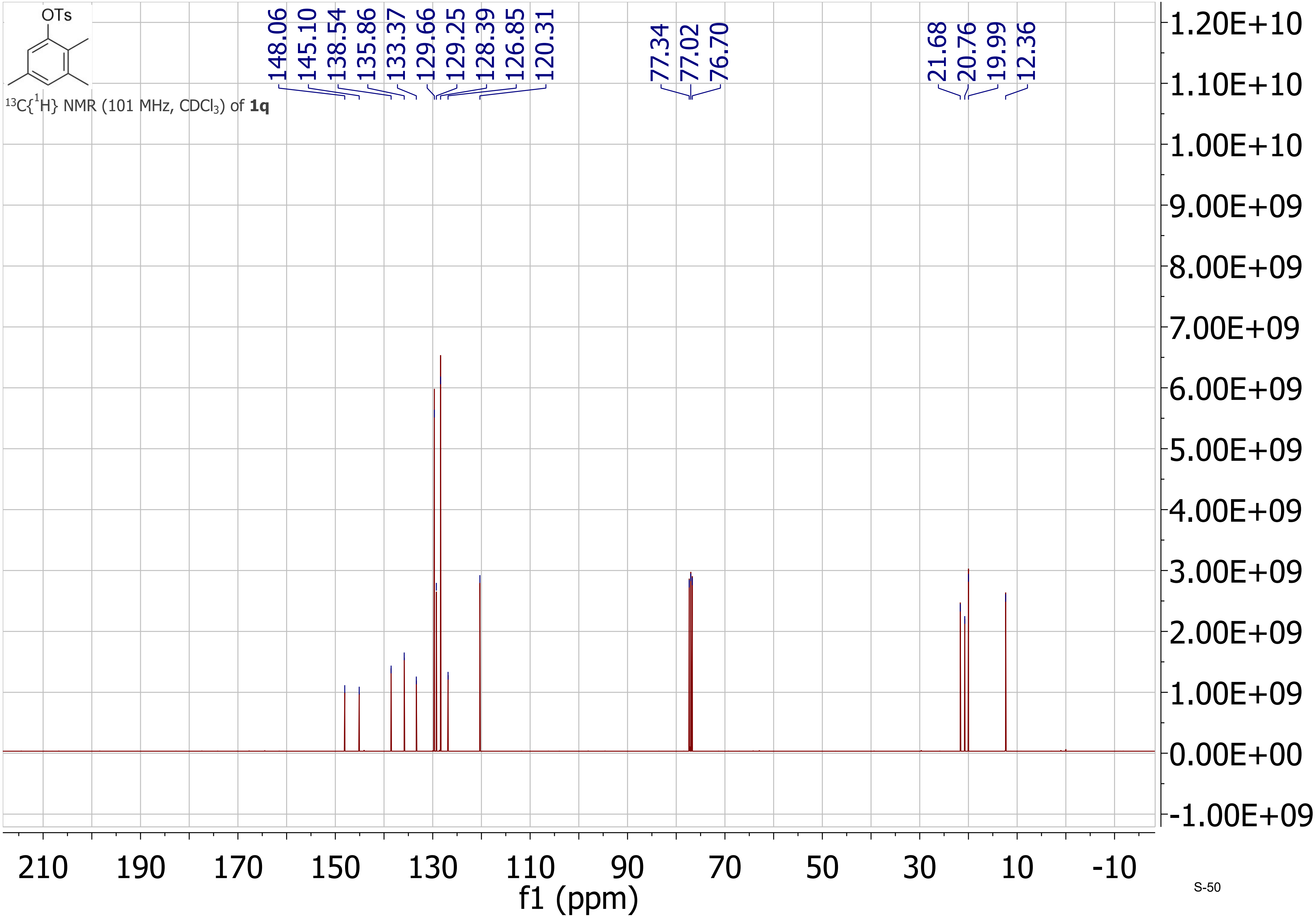
OTs
$^{13}C\{^{1}H\}$ NMR (101 MHz, $CDCl_3$) of **1q**
148.06
145.10
138.54
135.86
133.37
129.66
129.25
128.39
126.85
120.31
77.34
77.02
76.70
21.68
20.76
19.99
12.36
1.20E+10
1.10E+10
1.00E+10
9.00E+09
8.00E+09
7.00E+09
6.00E+09
5.00E+09
4.00E+09
3.00E+09
2.00E+09
1.00E+09
0.00E+00
-1.00E+09
210
190
170
150
130
110
90
70
50
30
10
-10
f1 (ppm)

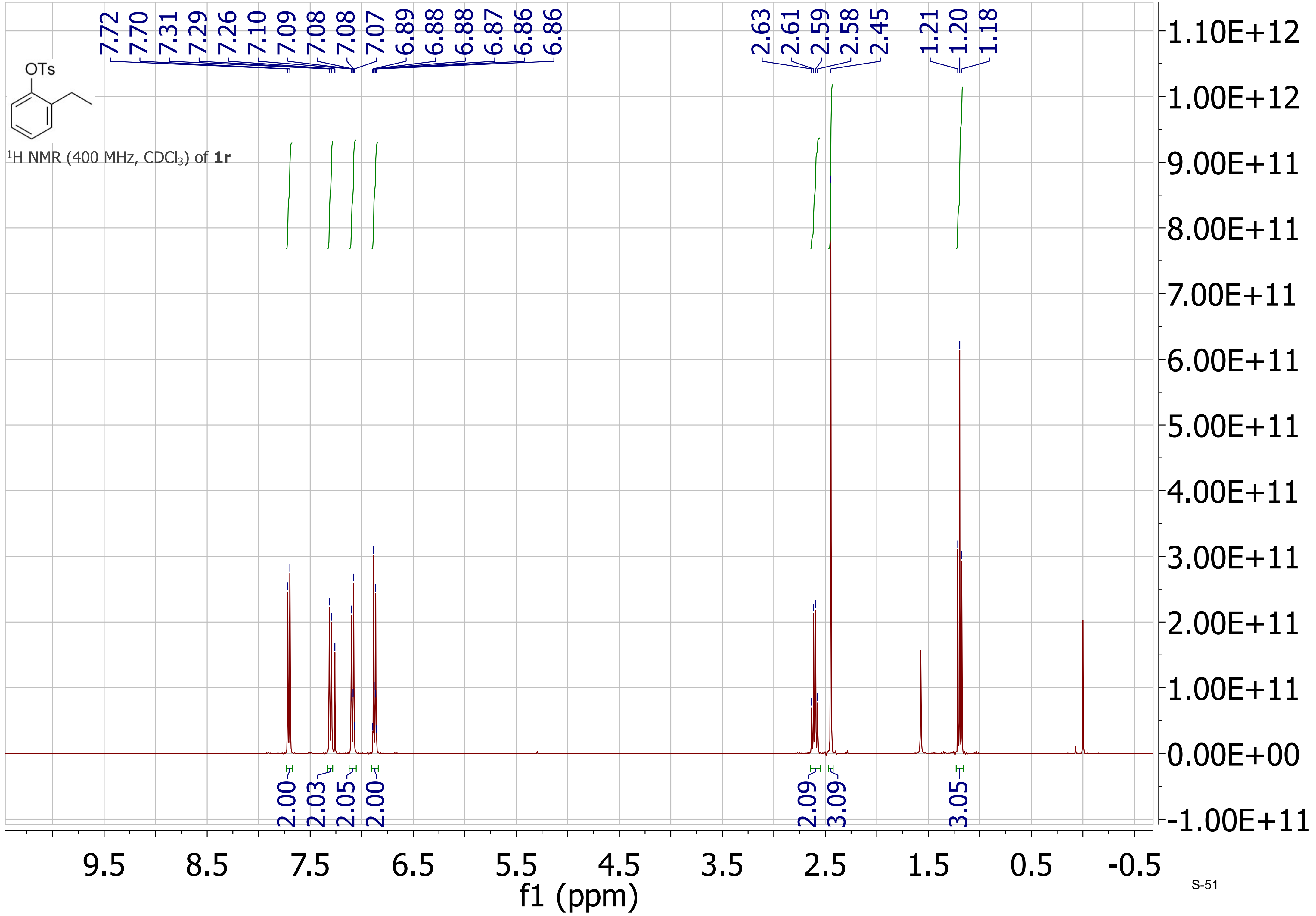
OTs
^{1}H NMR (400 MHz, $CDCl_3$) of **1r**
7.72 7.70 7.31 7.29 7.26 7.10 7.09 7.08 7.08 7.07 6.89 6.88 6.88 6.87 6.86 6.86
2.63 2.61 2.59 2.58 2.45
1.21 1.20 1.18
2.00 2.03 2.05 2.00
2.09 3.09
3.05
1.10E+12
1.00E+12
9.00E+11
8.00E+11
7.00E+11
6.00E+11
5.00E+11
4.00E+11
3.00E+11
2.00E+11
1.00E+11
0.00E+00
-1.00E+11
9.5 8.5 7.5 6.5 5.5 4.5 3.5 2.5 1.5 0.5 -0.5
f1 (ppm)

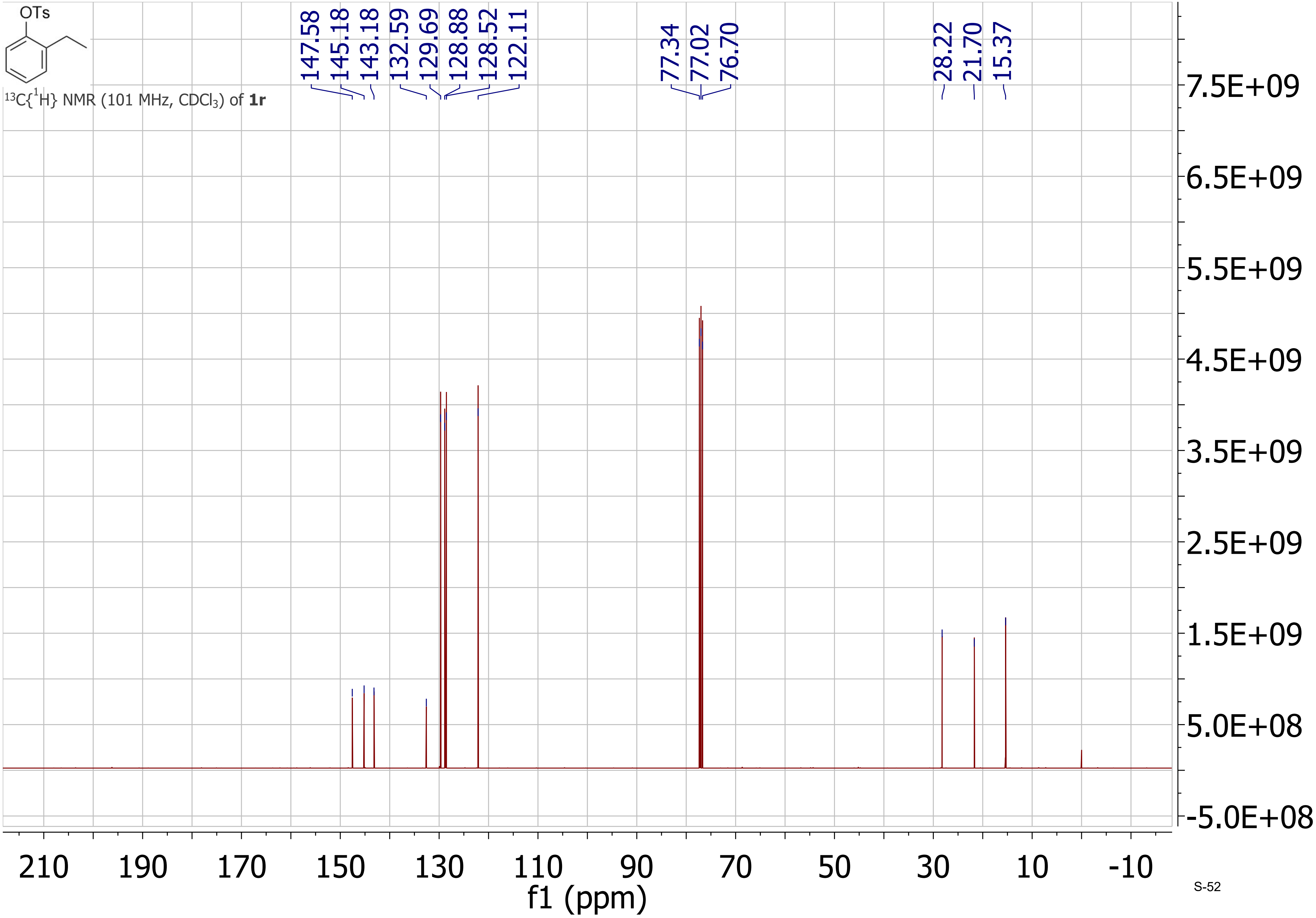
OTs
13C{1H} NMR (101 MHz, CDCl3) of 1r
147.58
145.18
143.18
132.59
129.69
128.88
128.52
122.11
77.34
77.02
76.70
28.22
21.70
15.37
7.5E+09
6.5E+09
5.5E+09
4.5E+09
3.5E+09
2.5E+09
1.5E+09
5.0E+08
-5.0E+08
210
190
170
150
130
110
90
70
50
30
10
-10
f1 (ppm)

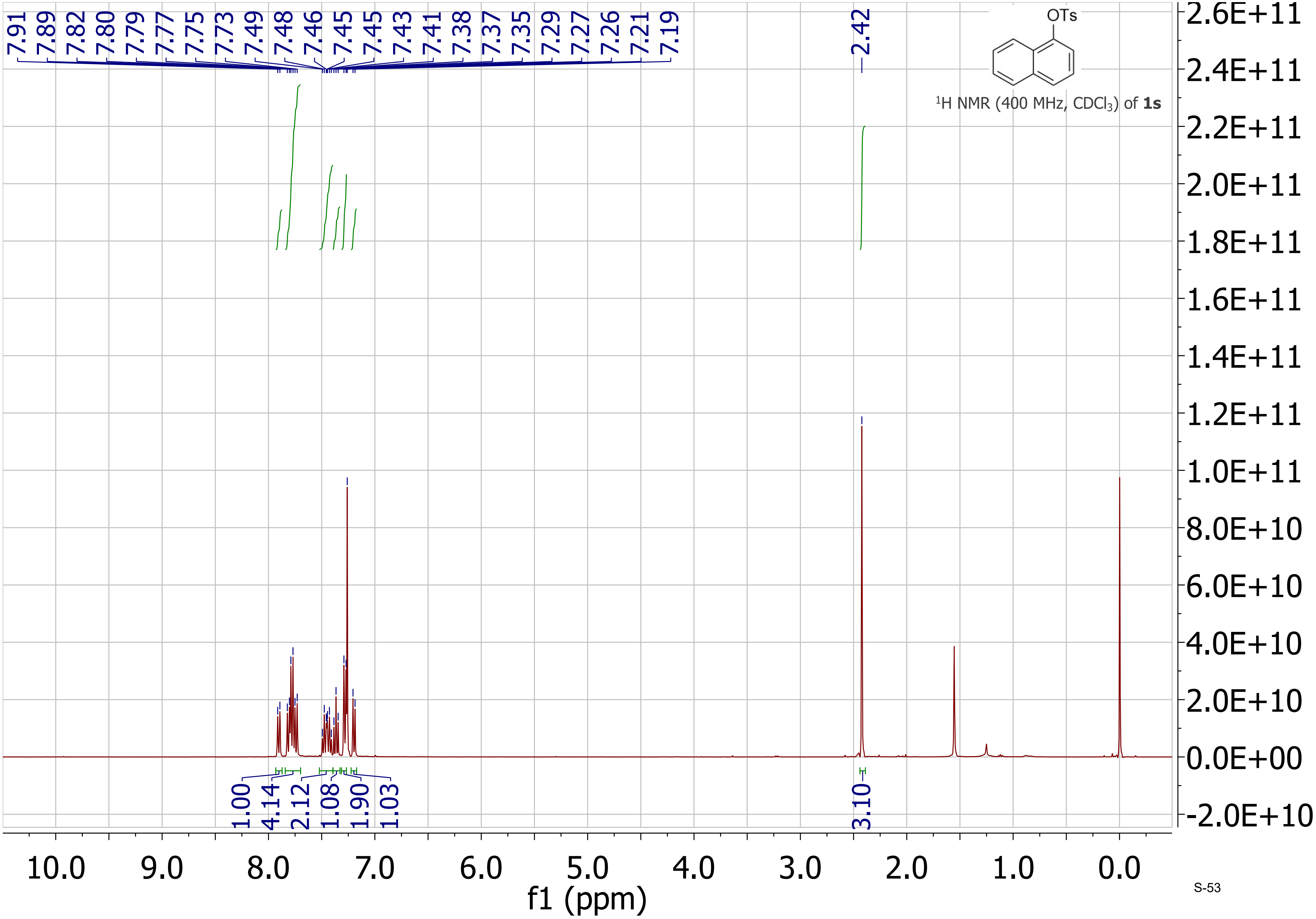
7.91
7.89
7.82
7.80
7.79
7.77
7.75
7.73
7.49
7.48
7.46
7.45
7.45
7.43
7.41
7.38
7.37
7.35
7.29
7.27
7.26
7.21
7.19
2.42
OTs
¹H NMR (400 MHz, CDCl₃) of 1s
1.00
4.14
2.12
1.08
1.90
1.03
3.10
f1 (ppm)

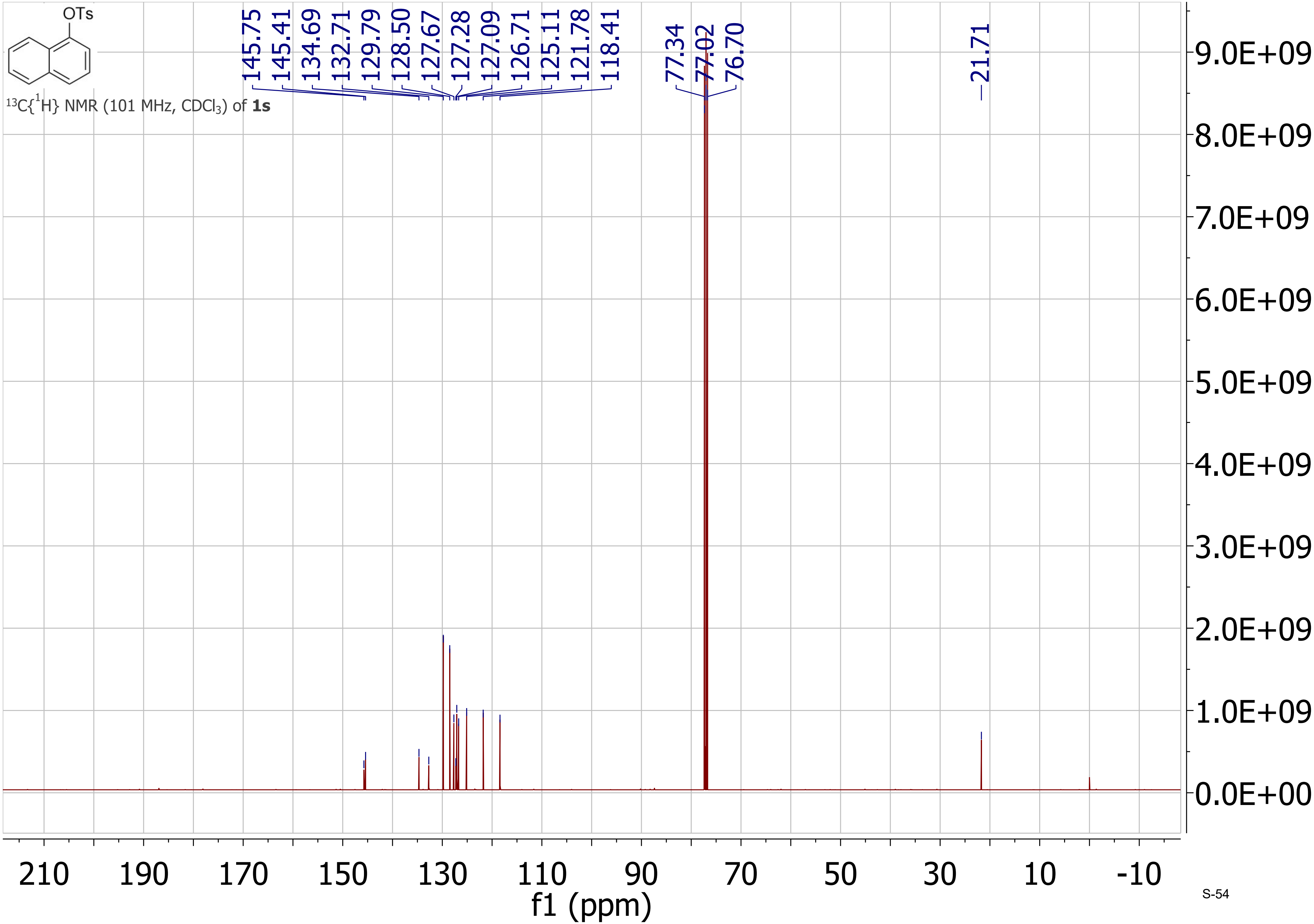
OTs
$^{13}C\{^1H\}$ NMR (101 MHz, $CDCl_3$) of **1s**
145.75
145.41
134.69
132.71
129.79
128.50
127.67
127.28
127.09
126.71
125.11
121.78
118.41
77.34
77.02
76.70
21.71
9.0E+09
8.0E+09
7.0E+09
6.0E+09
5.0E+09
4.0E+09
3.0E+09
2.0E+09
1.0E+09
0.0E+00
210
190
170
150
130
110
90
70
50
30
10
-10
f1 (ppm)

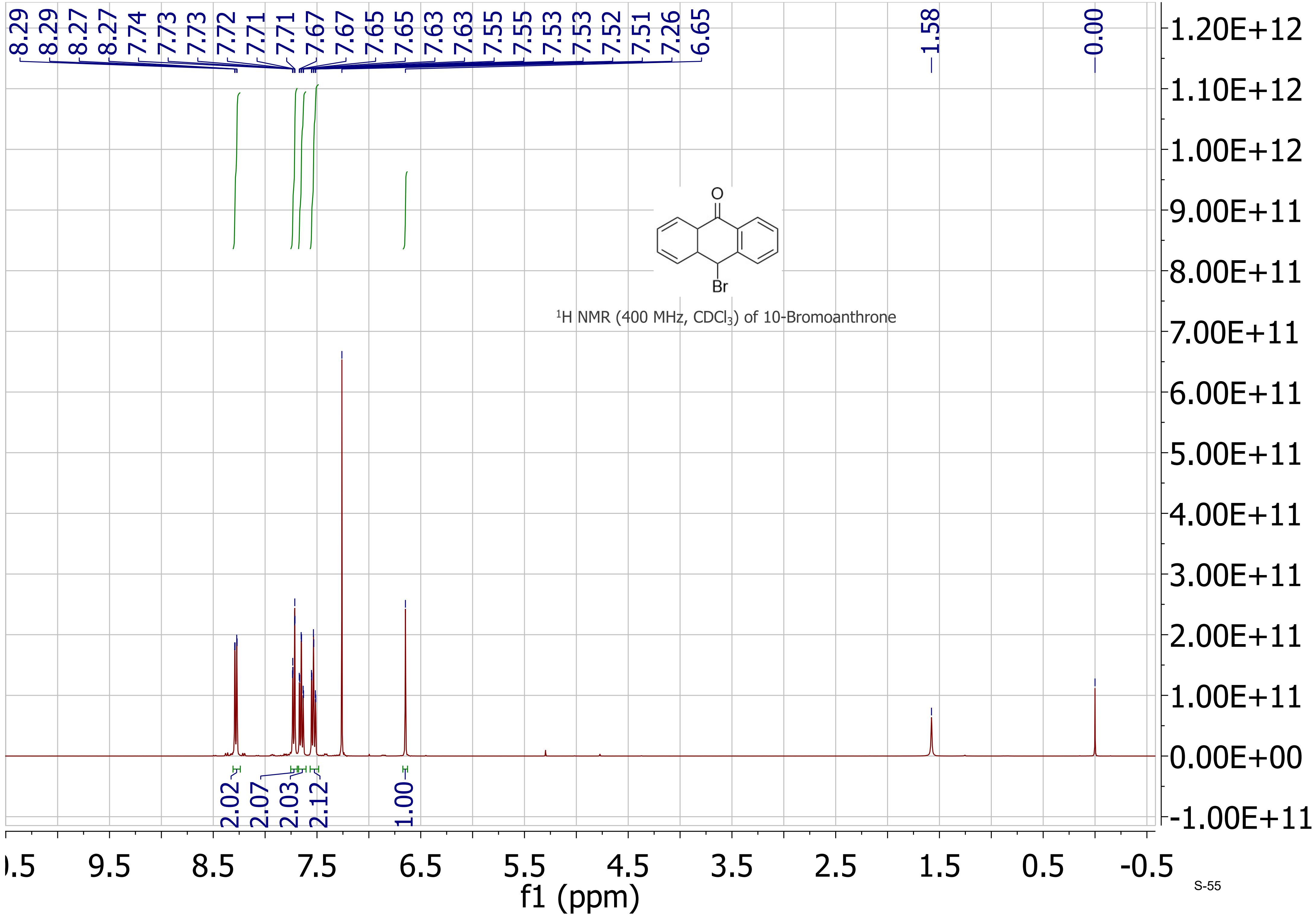

^{1}H NMR (400 MHz, $CDCl_3$) of 10-Bromoanthrone

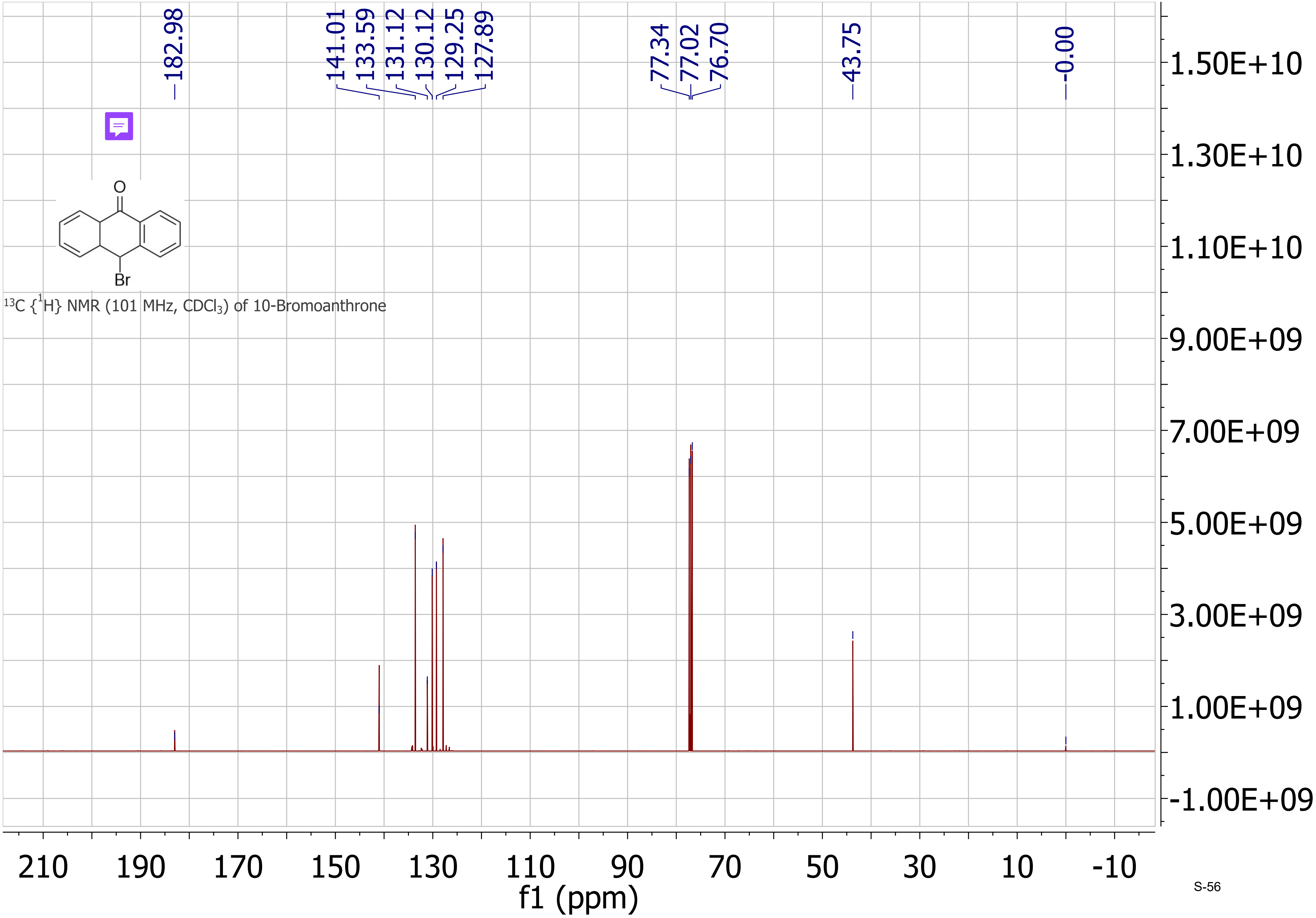
182.98
141.01
133.59
131.12
130.12
129.25
127.89
77.34
77.02
76.70
43.75
0.00
O
Br
13C {1H} NMR (101 MHz, CDCl3) of 10-Bromoanthrone
1.50E+10
1.30E+10
1.10E+10
9.00E+09
7.00E+09
5.00E+09
3.00E+09
1.00E+09
-1.00E+09
210
190
170
150
130
110
90
70
50
30
10
-10
f1 (ppm)

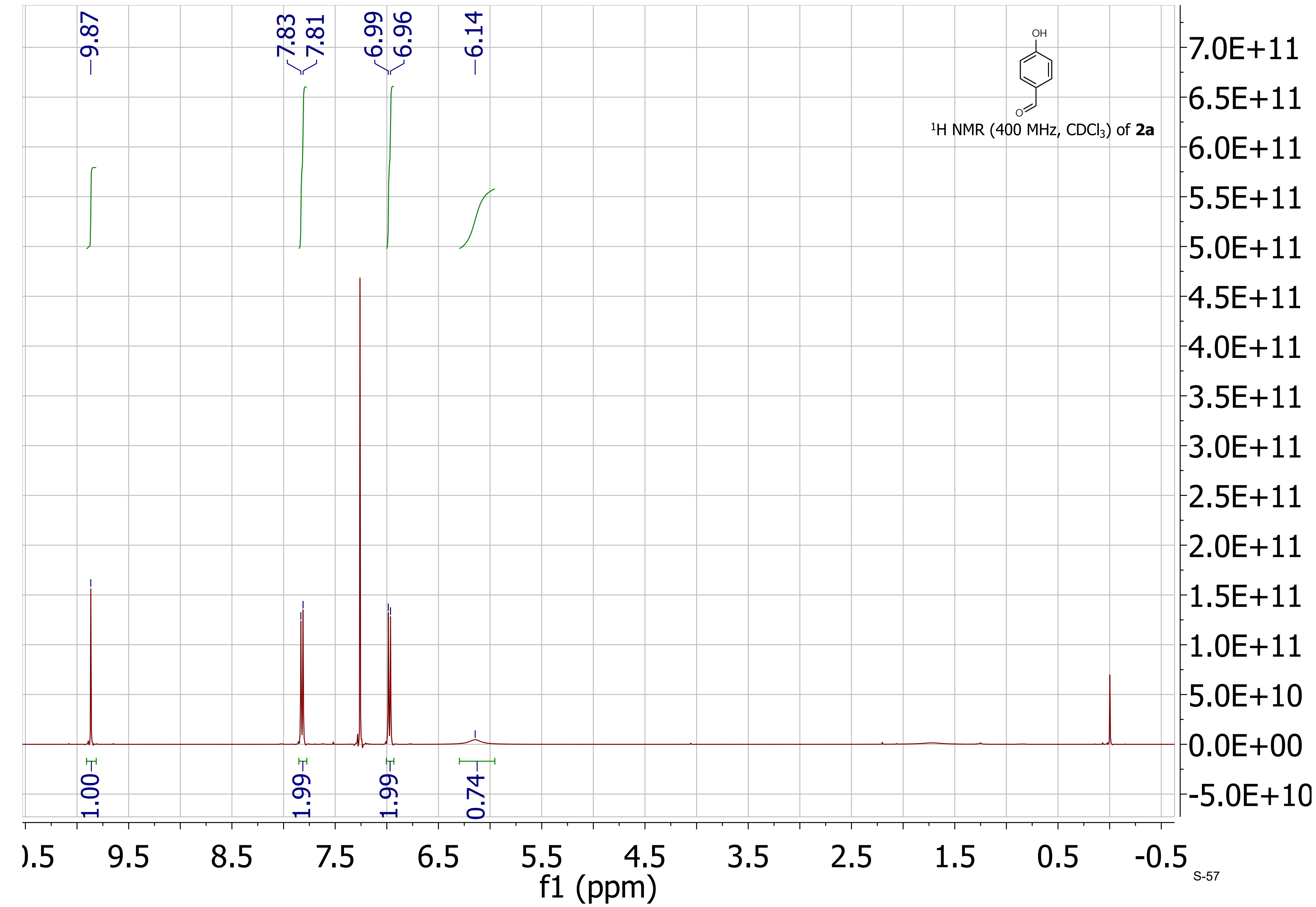
9.87
7.83
7.81
6.99
6.96
6.14
OH
O
^{1}H NMR (400 MHz, $CDCl_3$) of **2a**
1.00
1.99
1.99
0.74
f1 (ppm)

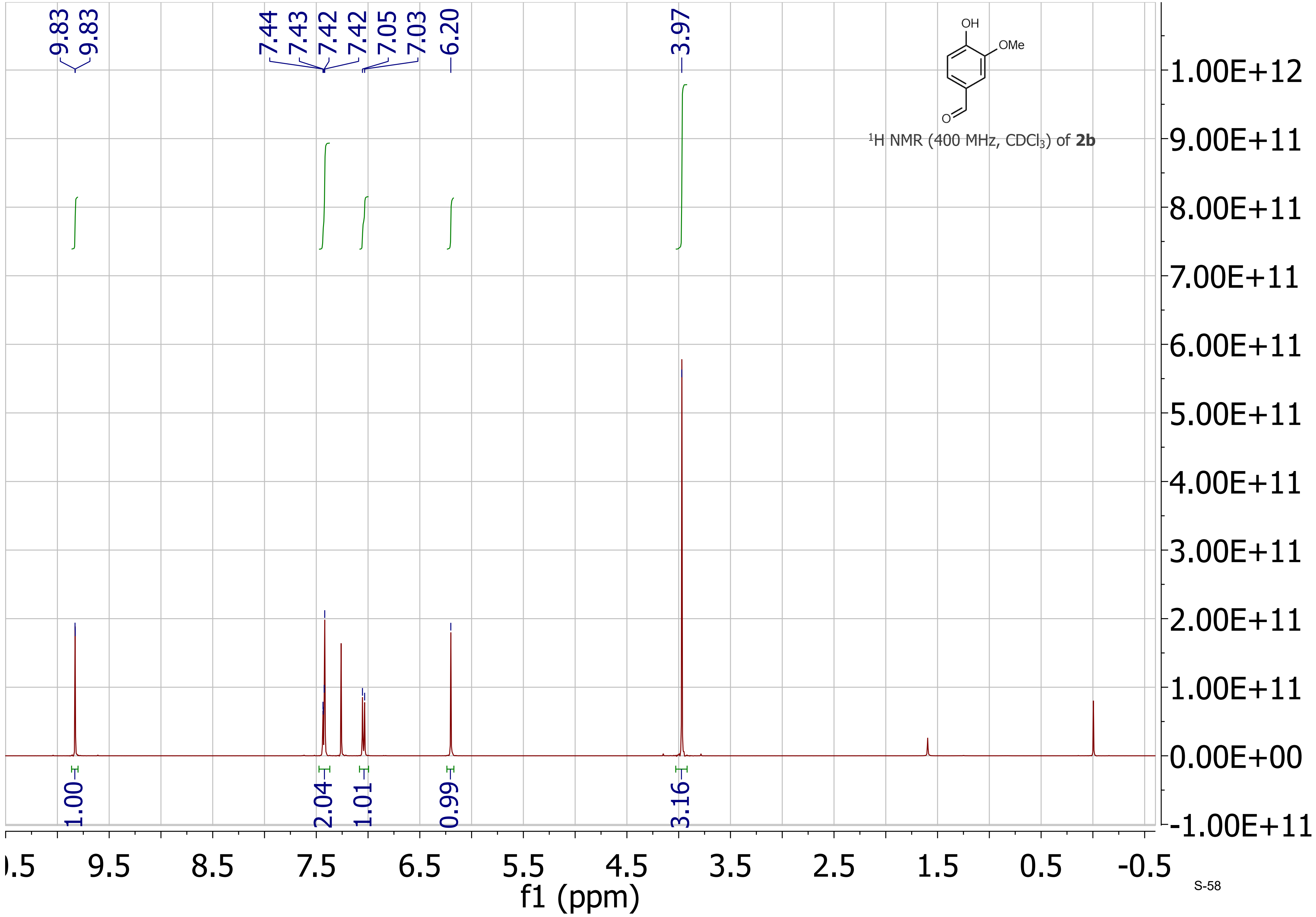
9.83
9.83
7.44
7.43
7.42
7.42
7.05
7.03
6.20
3.97
OH
OMe
O
¹H NMR (400 MHz, CDCl₃) of 2b
1.00
2.04
1.01
0.99
3.16
1.00E+12
9.00E+11
8.00E+11
7.00E+11
6.00E+11
5.00E+11
4.00E+11
3.00E+11
2.00E+11
1.00E+11
0.00E+00
-1.00E+11
9.5
8.5
7.5
6.5
5.5
4.5
3.5
2.5
1.5
0.5
-0.5
f1 (ppm)

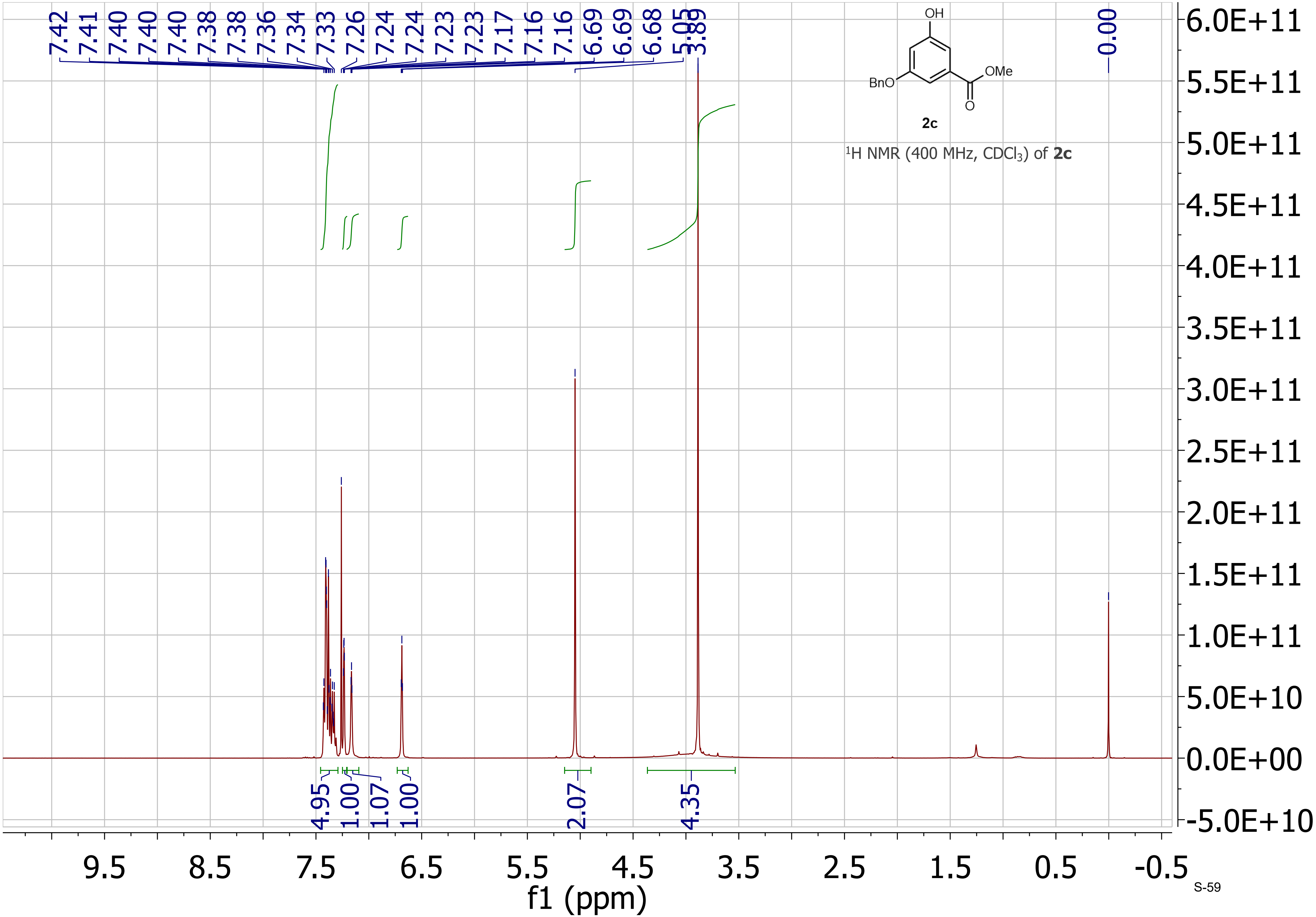

^1H NMR (400 MHz, $CDCl_3$) of **2c**

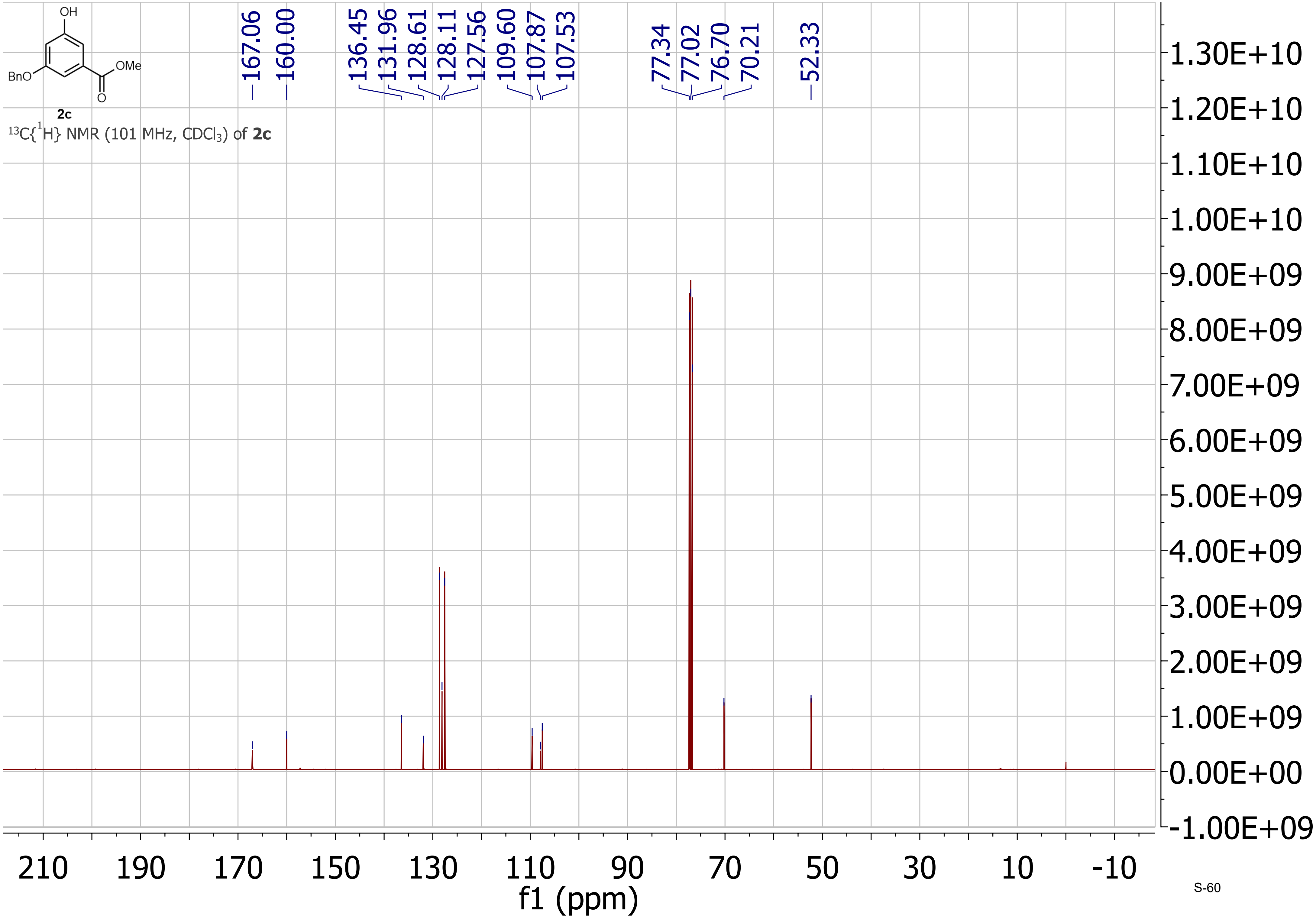
OH
BnO
OMe
O
2c
13C{1H} NMR (101 MHz, CDCl3) of 2c
167.06
160.00
136.45
131.96
128.61
128.11
127.56
109.60
107.87
107.53
77.34
77.02
76.70
70.21
52.33
1.30E+10
1.20E+10
1.10E+10
1.00E+10
9.00E+09
8.00E+09
7.00E+09
6.00E+09
5.00E+09
4.00E+09
3.00E+09
2.00E+09
1.00E+09
0.00E+00
-1.00E+09
210
190
170
150
130
110
90
70
50
30
10
-10
f1 (ppm)

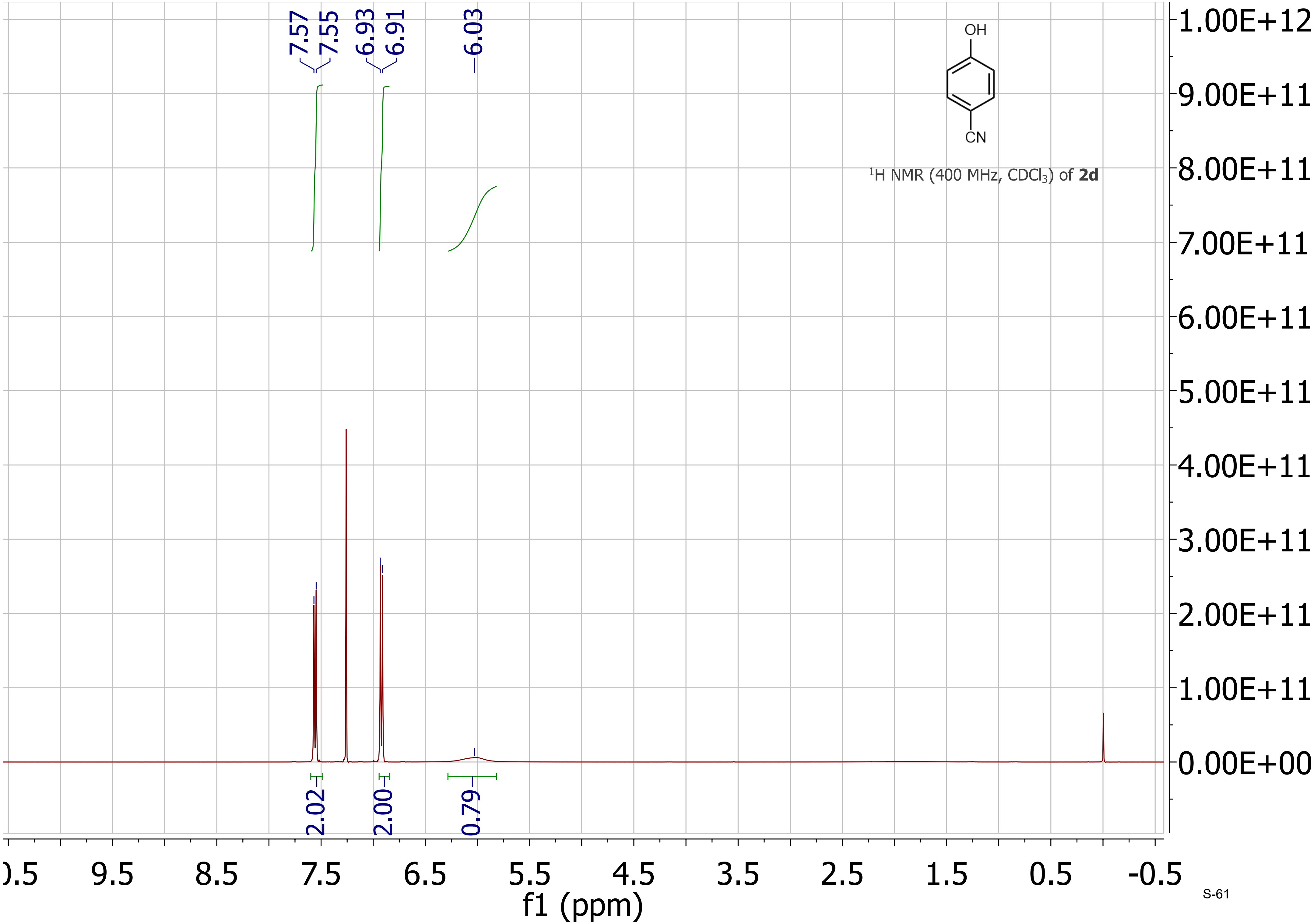

^1H NMR (400 MHz, $CDCl_3$) of **2d**

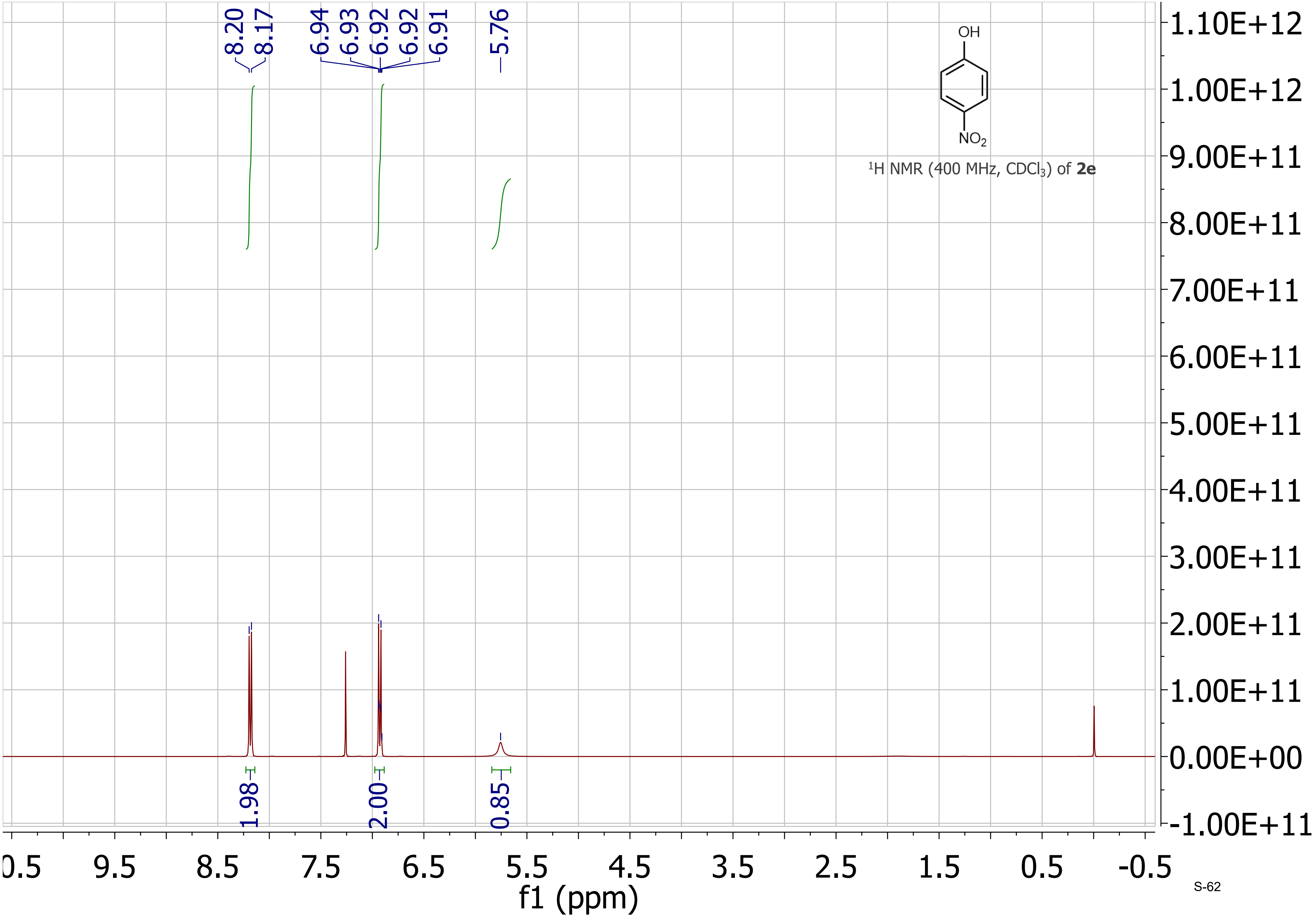
8.20
8.17
6.94
6.93
6.92
6.92
6.91
5.76
OH
NO2
1H NMR (400 MHz, CDCl3) of 2e
1.98
2.00
0.85
1.10E+12
1.00E+12
9.00E+11
8.00E+11
7.00E+11
6.00E+11
5.00E+11
4.00E+11
3.00E+11
2.00E+11
1.00E+11
0.00E+00
-1.00E+11
9.5
8.5
7.5
6.5
5.5
4.5
3.5
2.5
1.5
0.5
-0.5
f1 (ppm)

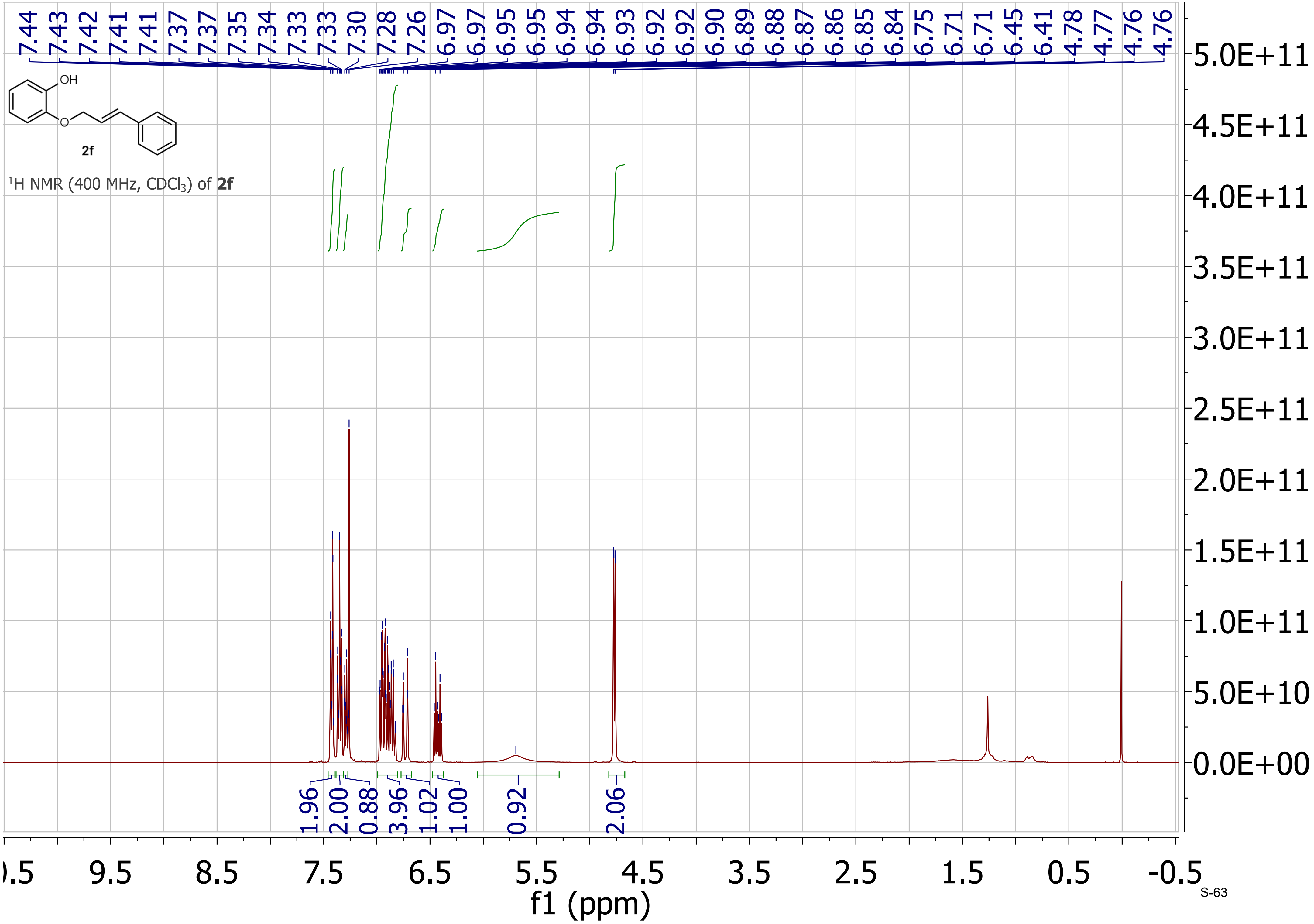
7.44 7.43 7.42 7.41 7.41 7.37 7.37 7.35 7.34 7.33 7.33 7.30 7.28 7.26 6.97 6.97 6.95 6.95 6.94 6.94 6.93 6.92 6.92 6.90 6.89 6.88 6.87 6.86 6.85 6.84 6.75 6.71 6.71 6.45 6.41 4.78 4.77 4.76 4.76
OH
O
2f
1H NMR (400 MHz, CDCl3) of 2f
1.96 2.00 0.88 3.96 1.02 1.00 0.92 2.06
f1 (ppm)
5.0E+11 4.5E+11 4.0E+11 3.5E+11 3.0E+11 2.5E+11 2.0E+11 1.5E+11 1.0E+11 5.0E+10 0.0E+00
9.5 8.5 7.5 6.5 5.5 4.5 3.5 2.5 1.5 0.5 -0.5

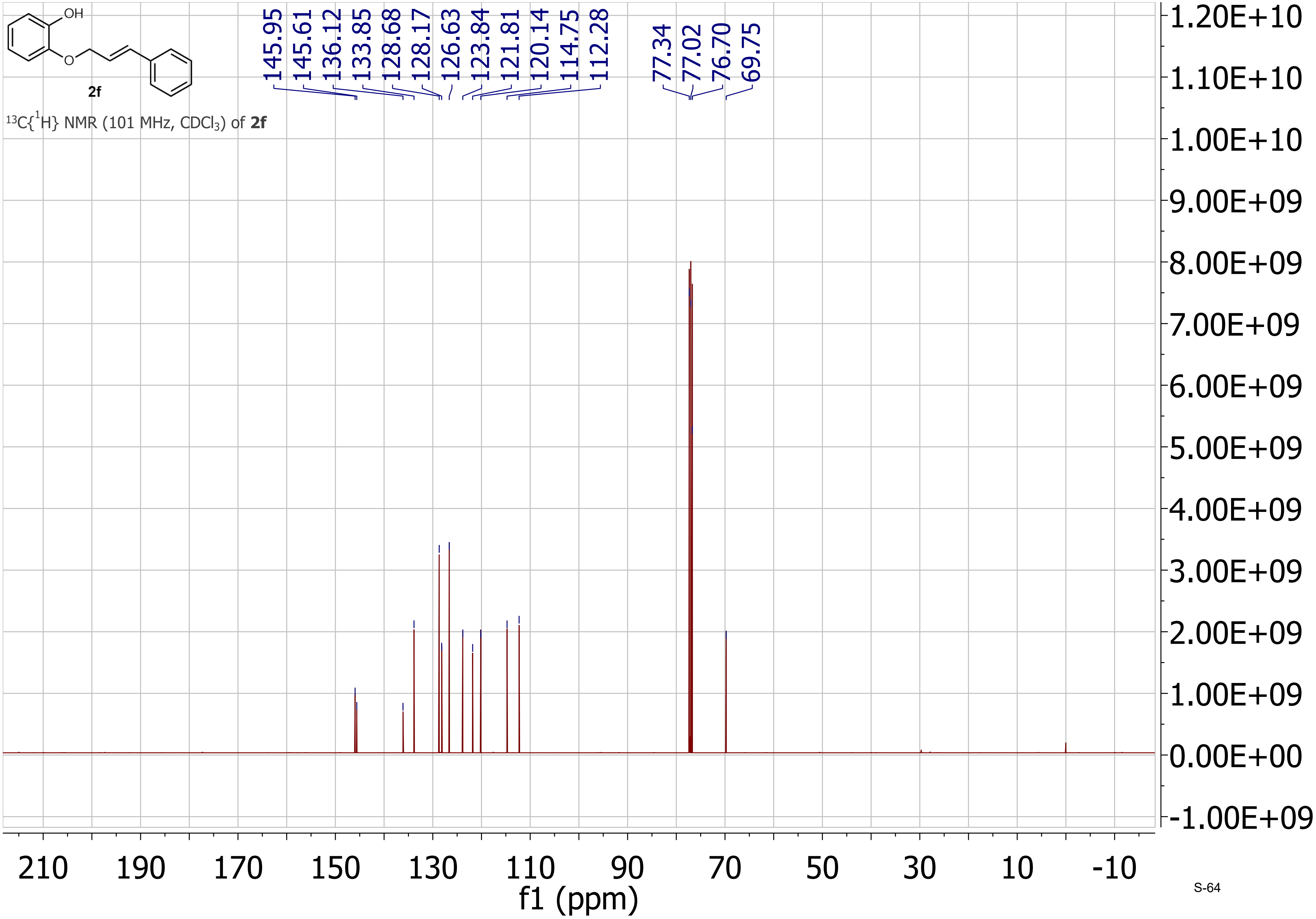
OH
O
2f
13C{1H} NMR (101 MHz, CDCl3) of 2f
145.95
145.61
136.12
133.85
128.68
128.17
126.63
123.84
121.81
120.14
114.75
112.28
77.34
77.02
76.70
69.75
1.20E+10
1.10E+10
1.00E+10
9.00E+09
8.00E+09
7.00E+09
6.00E+09
5.00E+09
4.00E+09
3.00E+09
2.00E+09
1.00E+09
0.00E+00
-1.00E+09
210
190
170
150
130
110
90
70
50
30
10
-10
f1 (ppm)

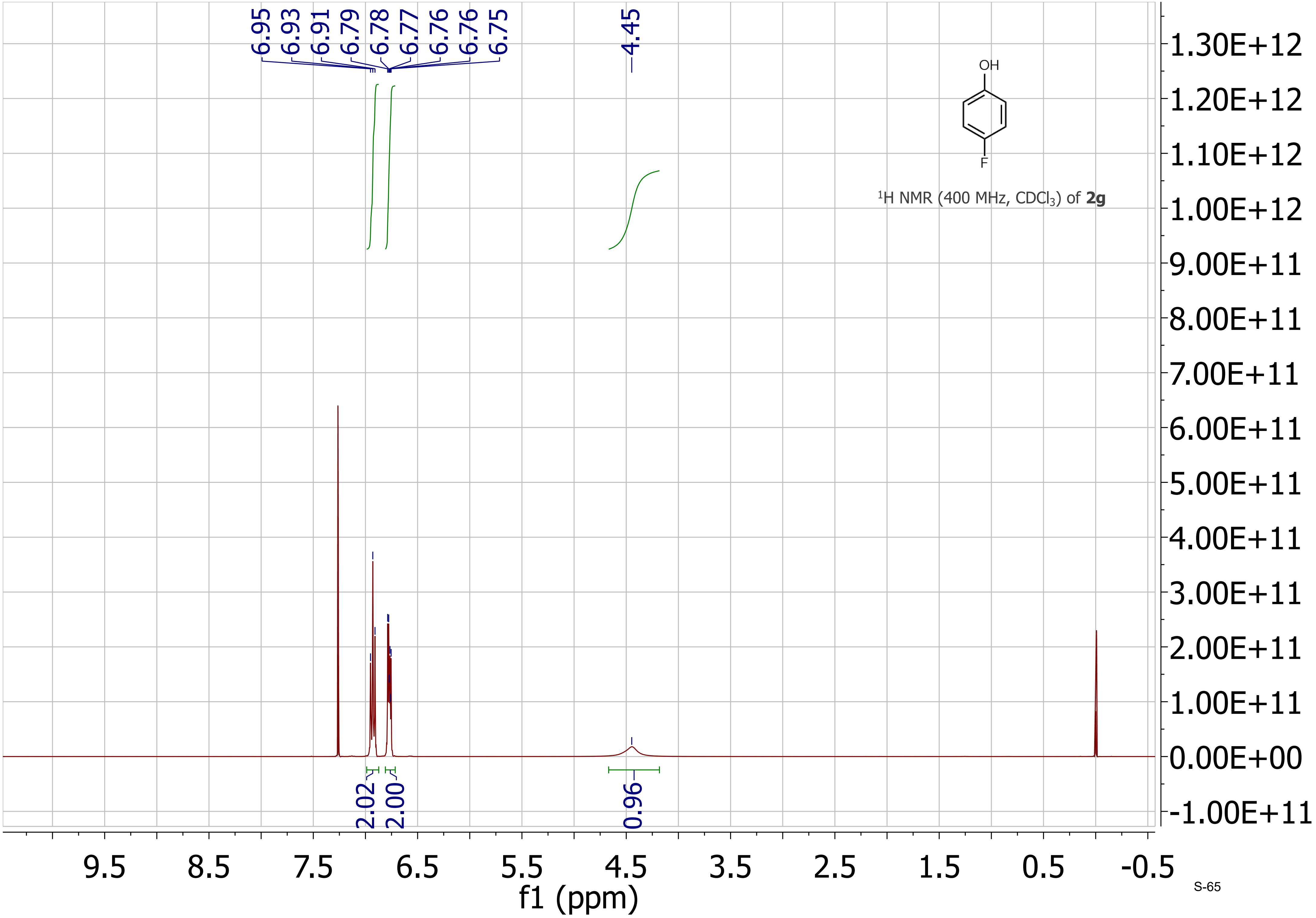

6.95
6.93
6.91
6.79
6.78
6.77
6.76
6.76
6.75
4.45
OH
F
¹H NMR (400 MHz, CDCl3) of **2g**
2.02
2.00
0.96
1.30E+12
1.20E+12
1.10E+12
1.00E+12
9.00E+11
8.00E+11
7.00E+11
6.00E+11
5.00E+11
4.00E+11
3.00E+11
2.00E+11
1.00E+11
0.00E+00
-1.00E+11
9.5
8.5
7.5
6.5
5.5
4.5
3.5
2.5
1.5
0.5
-0.5
f1 (ppm)

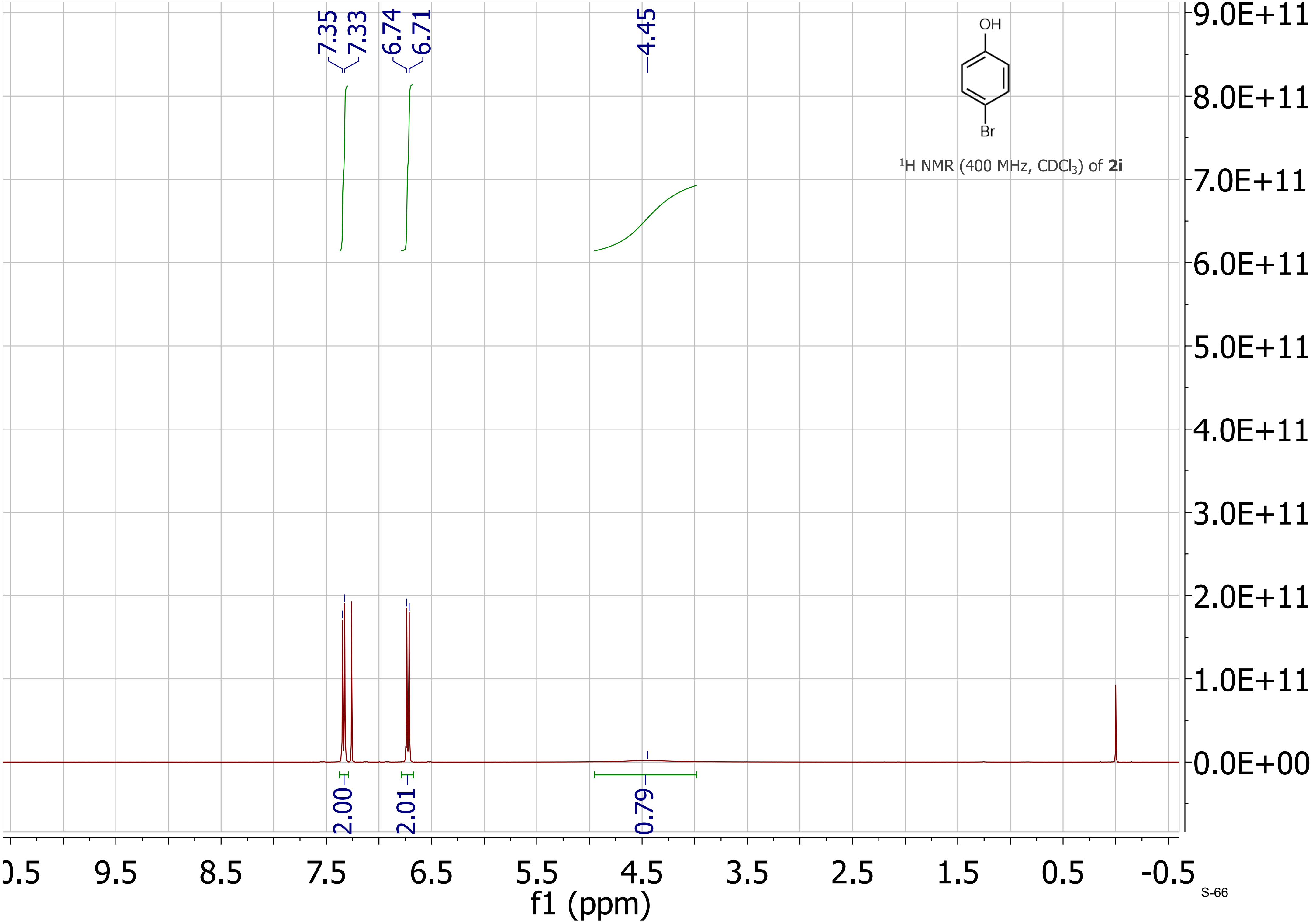

^{1}H NMR (400 MHz, $CDCl_3$) of **2i**

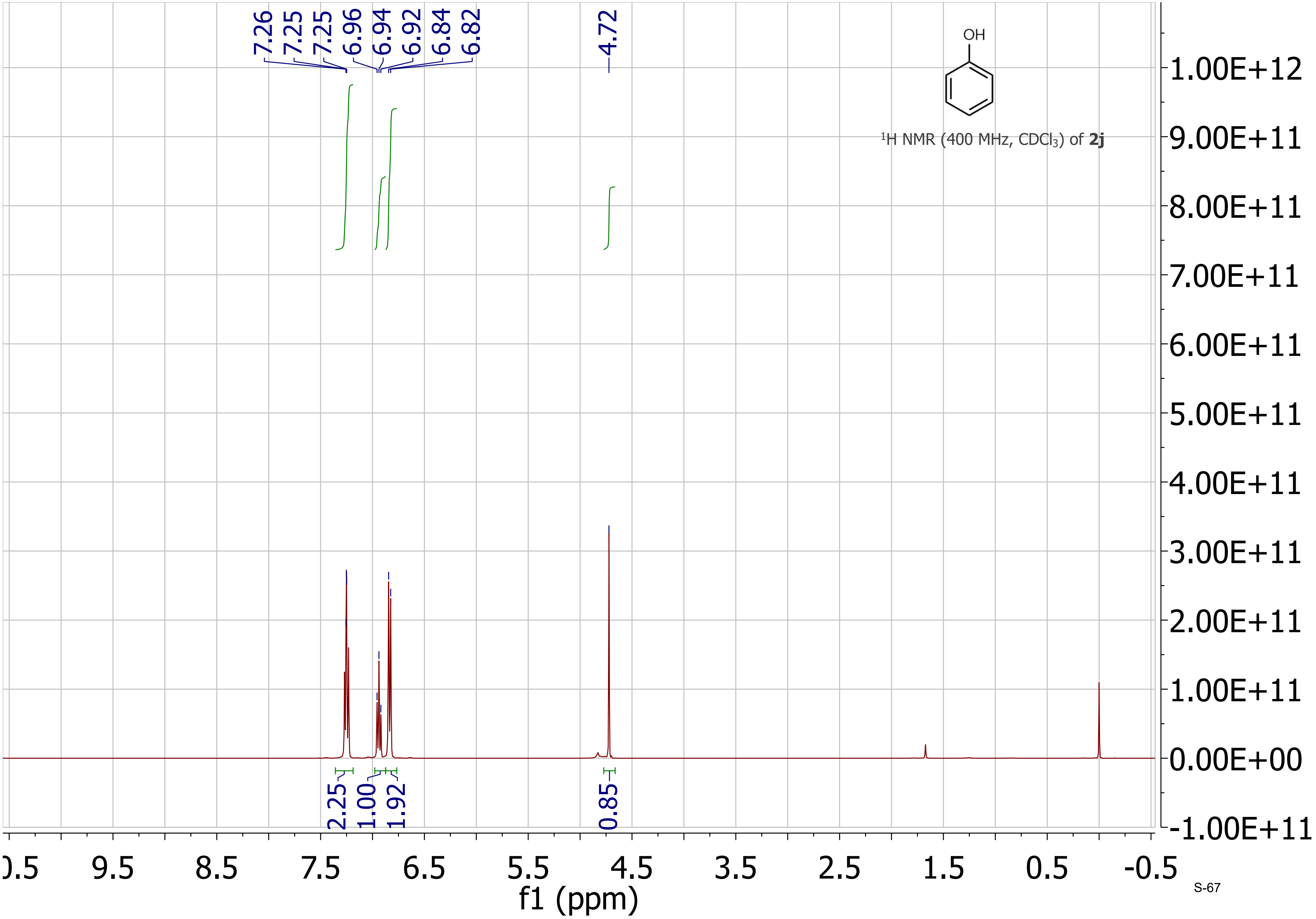
7.26
7.25
7.25
6.96
6.94
6.92
6.84
6.82
4.72
OH
^{1}H NMR (400 MHz, CDCl$_3$) of **2j**
2.25
1.00
1.92
0.85
f1 (ppm)

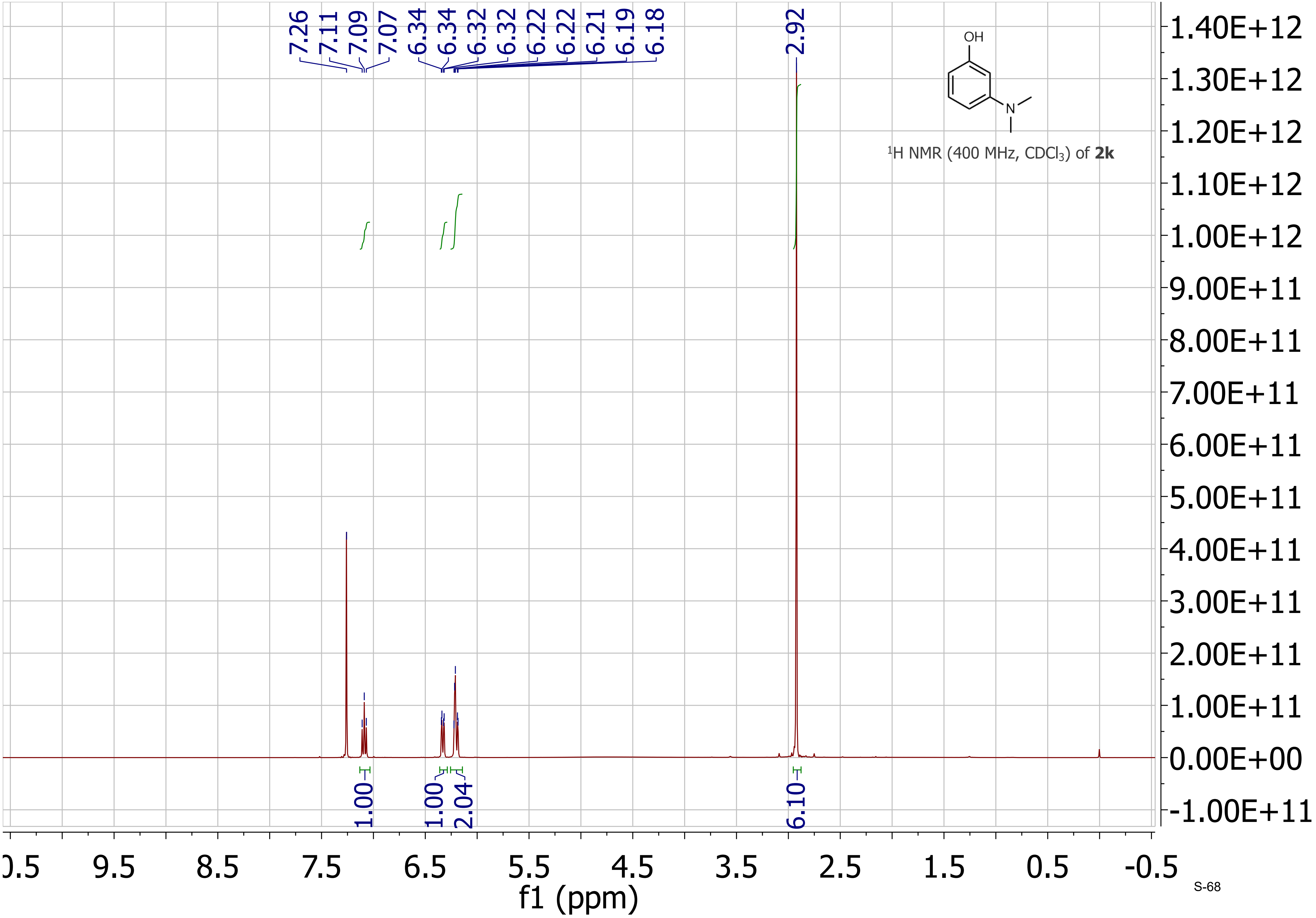
7.26
7.11
7.09
7.07
6.34
6.34
6.32
6.32
6.22
6.22
6.21
6.19
6.18
2.92
OH
N
^{1}H NMR (400 MHz, $CDCl_3$) of **2k**
1.40E+12
1.30E+12
1.20E+12
1.10E+12
1.00E+12
9.00E+11
8.00E+11
7.00E+11
6.00E+11
5.00E+11
4.00E+11
3.00E+11
2.00E+11
1.00E+11
0.00E+00
-1.00E+11
1.00
1.00
2.04
6.10
9.5
8.5
7.5
6.5
5.5
4.5
3.5
2.5
1.5
0.5
-0.5
f1 (ppm)

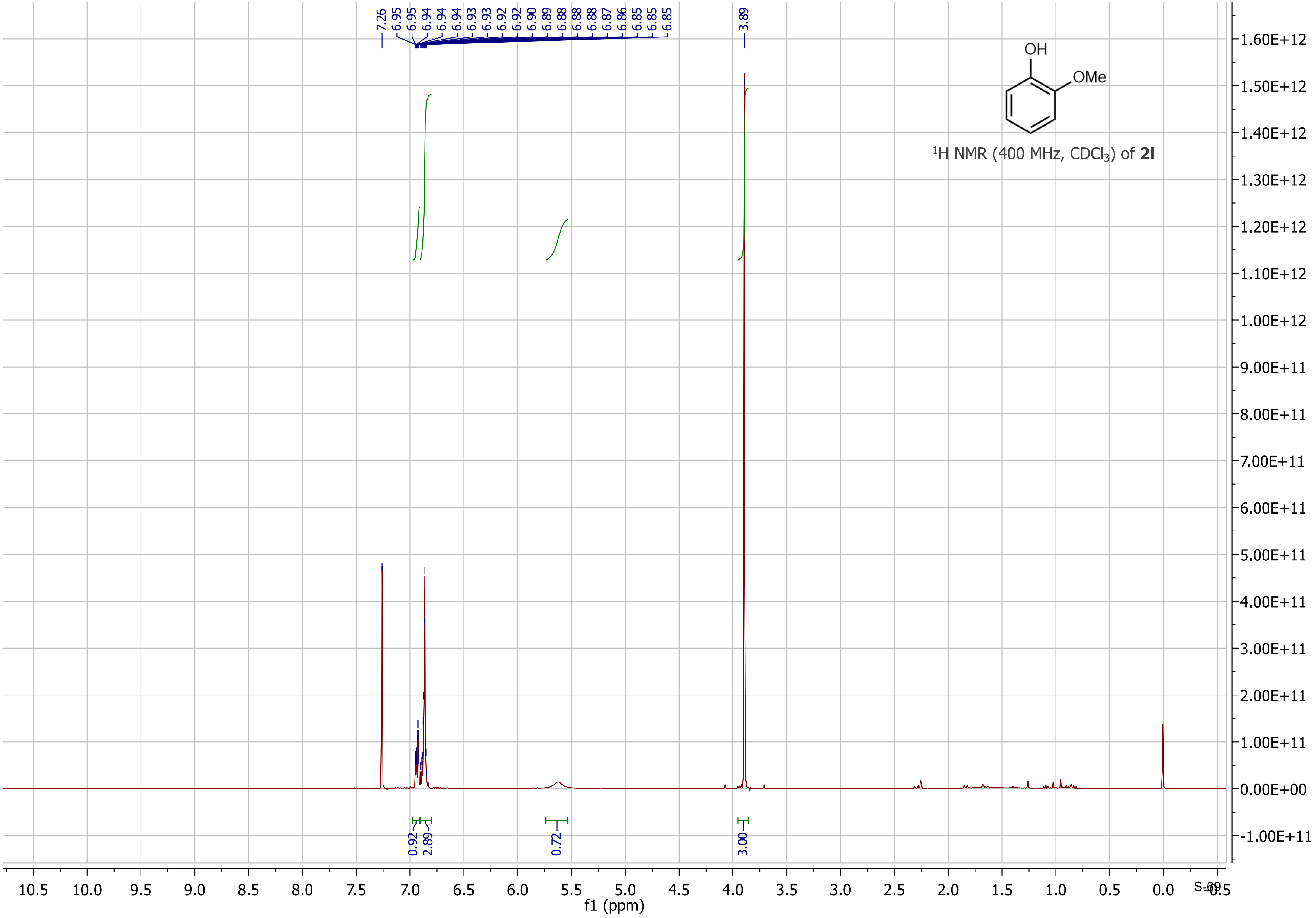

OH
OMe
^{1}H NMR (400 MHz, $CDCl_3$) of **2l**
7.26
6.95
6.95
6.94
6.94
6.93
6.93
6.92
6.92
6.90
6.89
6.88
6.88
6.88
6.87
6.86
6.85
6.85
6.85
3.89
0.92
2.89
0.72
3.00
f1 (ppm)

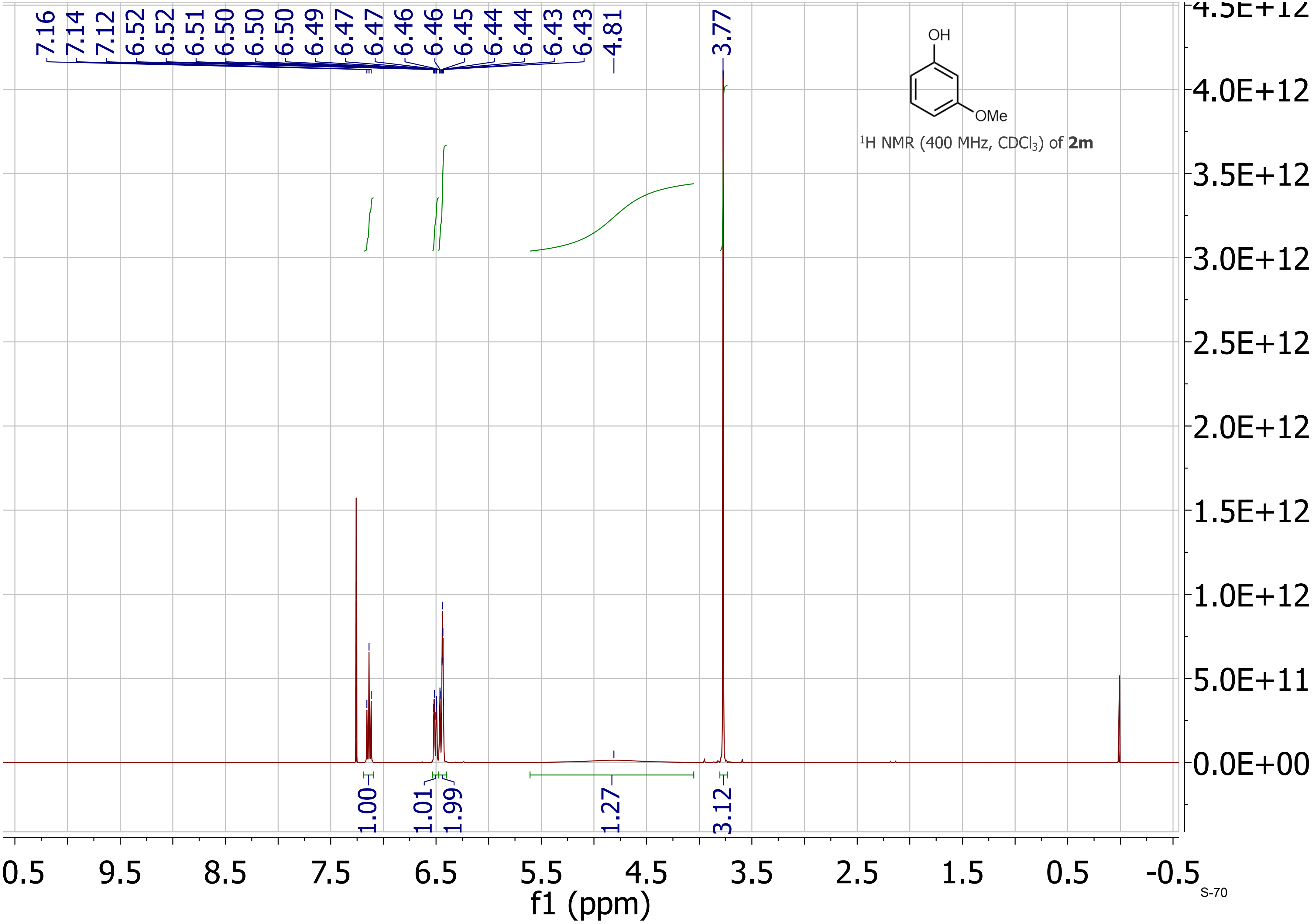
7.16
7.14
7.12
6.52
6.52
6.51
6.50
6.50
6.50
6.49
6.47
6.47
6.46
6.46
6.45
6.44
6.44
6.43
6.43
4.81
3.77
OH
OMe
^{1}H NMR (400 MHz, $CDCl_3$) of **2m**
1.00
1.01
1.99
1.27
3.12
f1 (ppm)

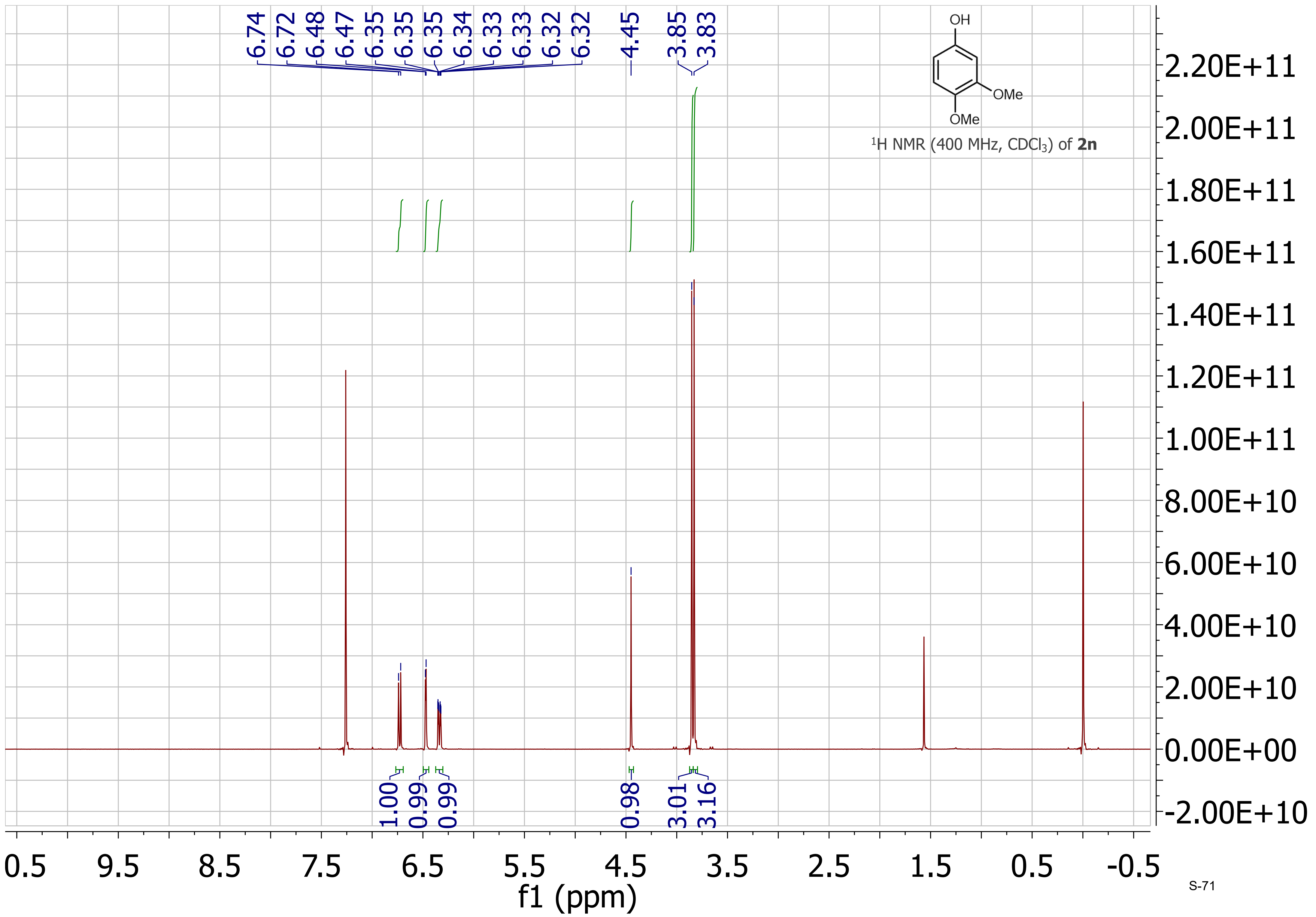
6.74
6.72
6.48
6.47
6.35
6.35
6.35
6.34
6.33
6.33
6.32
6.32
4.45
3.85
3.83
OH
OMe
OMe
^{1}H NMR (400 MHz, $CDCl_3$) of **2n**
1.00
0.99
0.99
0.98
3.01
3.16
f1 (ppm)

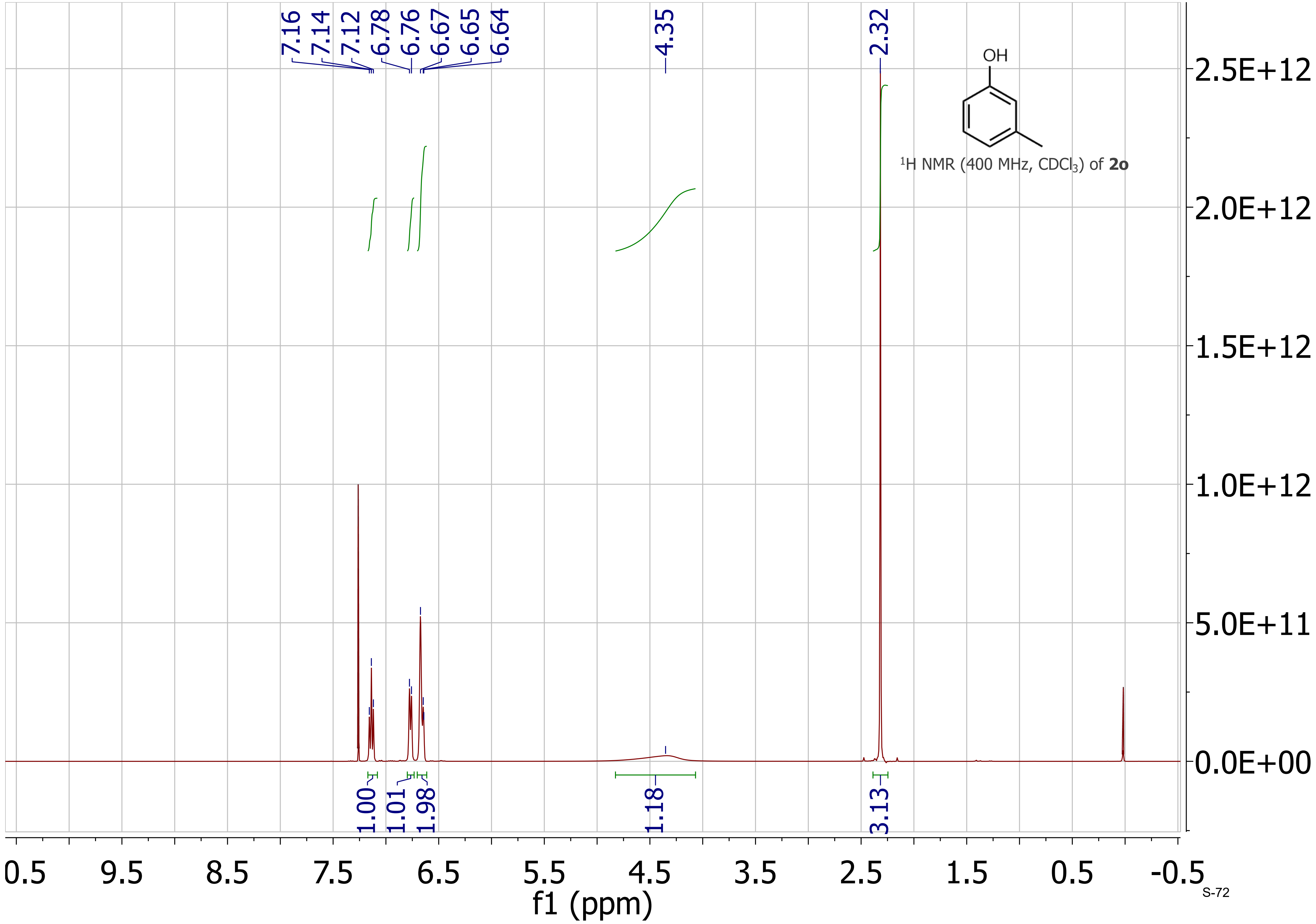
7.16
7.14
7.12
6.78
6.76
6.67
6.65
6.64
4.35
2.32
OH
1H NMR (400 MHz, CDCl3) of 2o
2.5E+12
2.0E+12
1.5E+12
1.0E+12
5.0E+11
0.0E+00
1.00
1.01
1.98
1.18
3.13
0.5
9.5
8.5
7.5
6.5
5.5
4.5
3.5
2.5
1.5
0.5
-0.5
f1 (ppm)

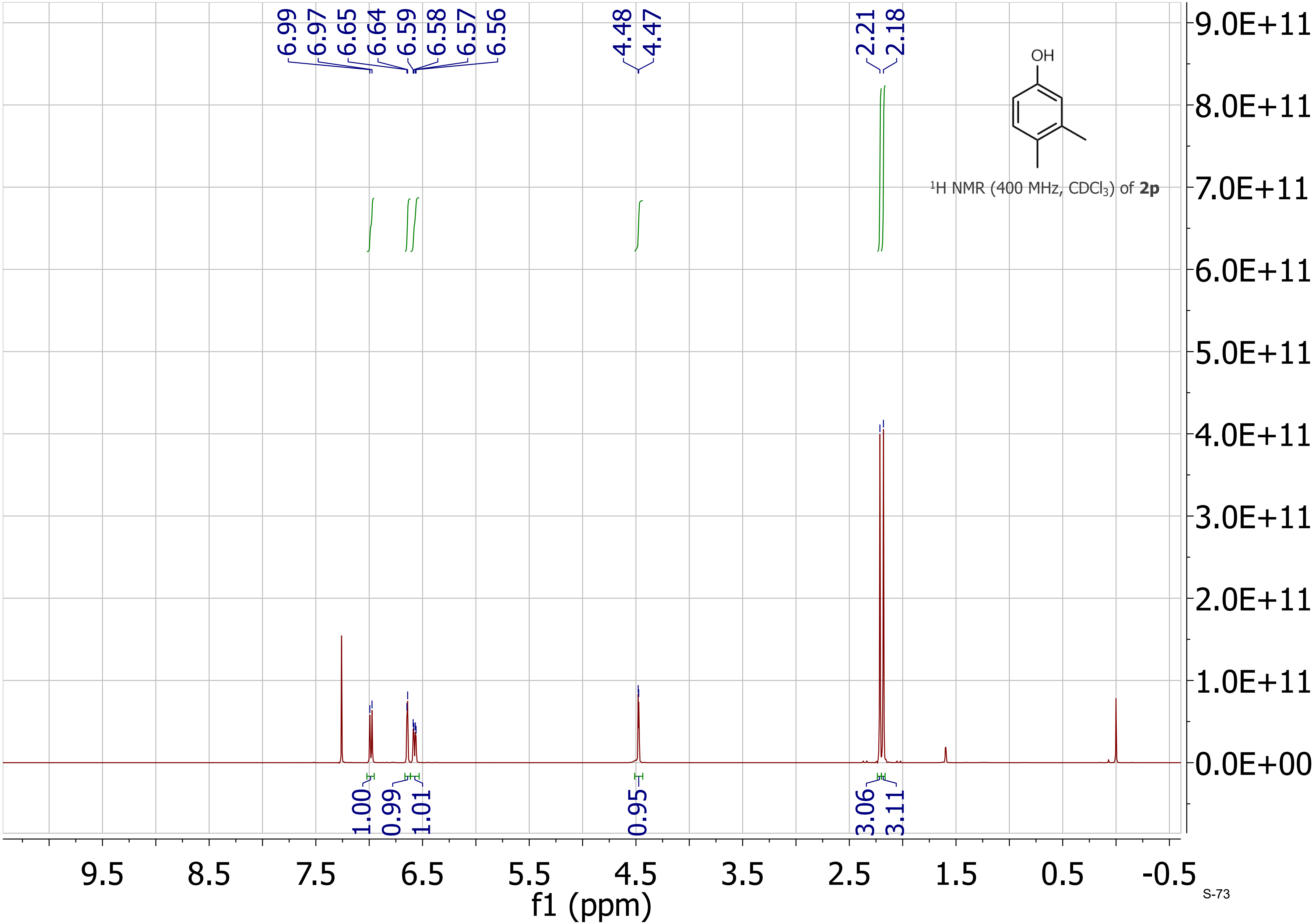
6.99
6.97
6.65
6.64
6.59
6.58
6.57
6.56
4.48
4.47
2.21
2.18
OH
¹H NMR (400 MHz, CDCl₃) of **2p**
1.00
0.99
1.01
0.95
3.06
3.11
9.0E+11
8.0E+11
7.0E+11
6.0E+11
5.0E+11
4.0E+11
3.0E+11
2.0E+11
1.0E+11
0.0E+00
9.5
8.5
7.5
6.5
5.5
4.5
3.5
2.5
1.5
0.5
-0.5
f1 (ppm)

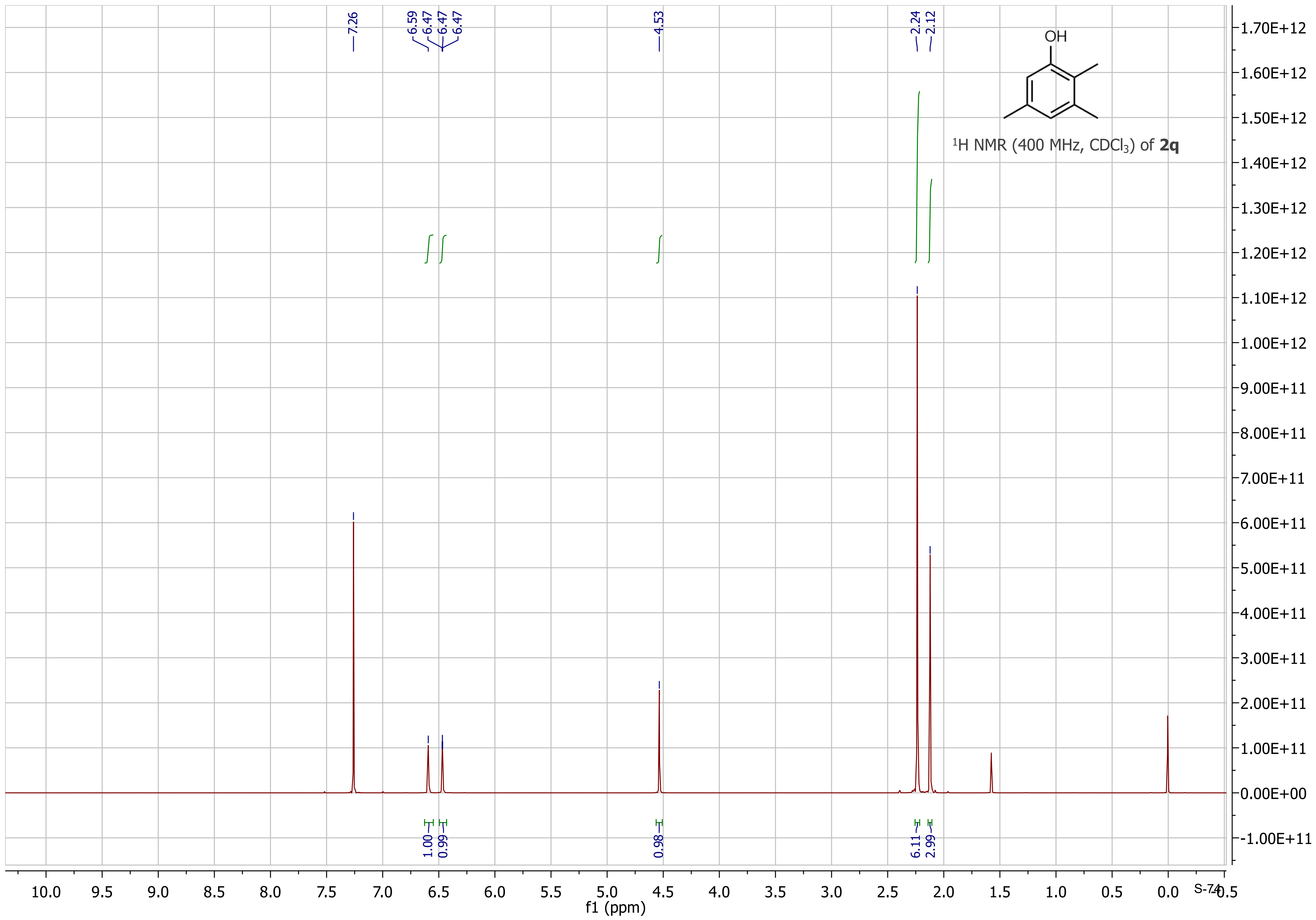

7.26
6.59
6.47
6.47
6.47
4.53
2.24
2.12
OH
1H NMR (400 MHz, CDCl3) of 2q
1.00
0.99
0.98
6.11
2.99
f1 (ppm)

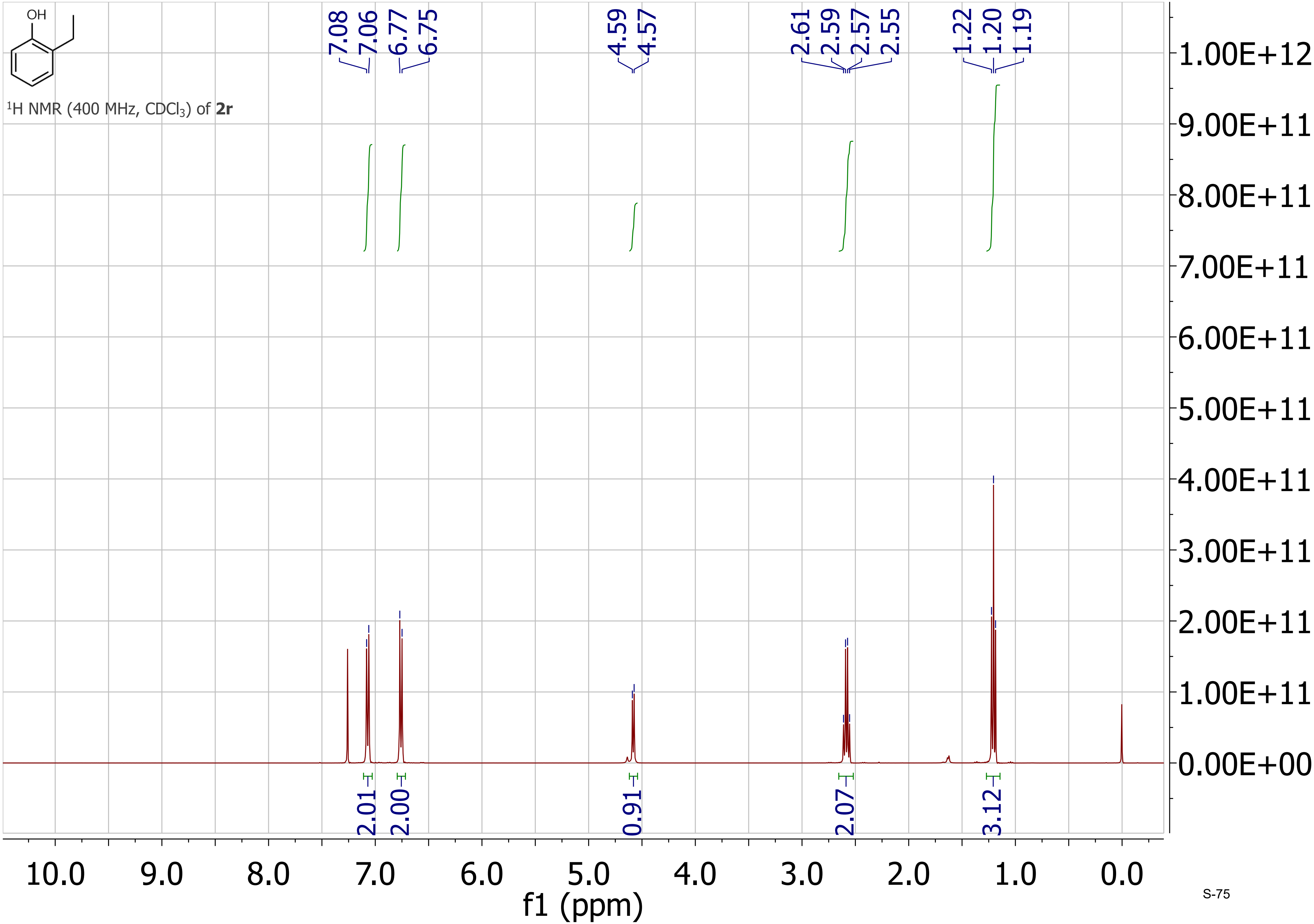

OH
1H NMR (400 MHz, CDCl3) of 2r
7.08
7.06
6.77
6.75
4.59
4.57
2.61
2.59
2.57
2.55
1.22
1.20
1.19
2.01
2.00
0.91
2.07
3.12
1.00E+12
9.00E+11
8.00E+11
7.00E+11
6.00E+11
5.00E+11
4.00E+11
3.00E+11
2.00E+11
1.00E+11
0.00E+00
10.0
9.0
8.0
7.0
6.0
5.0
4.0
3.0
2.0
1.0
0.0
f1 (ppm)

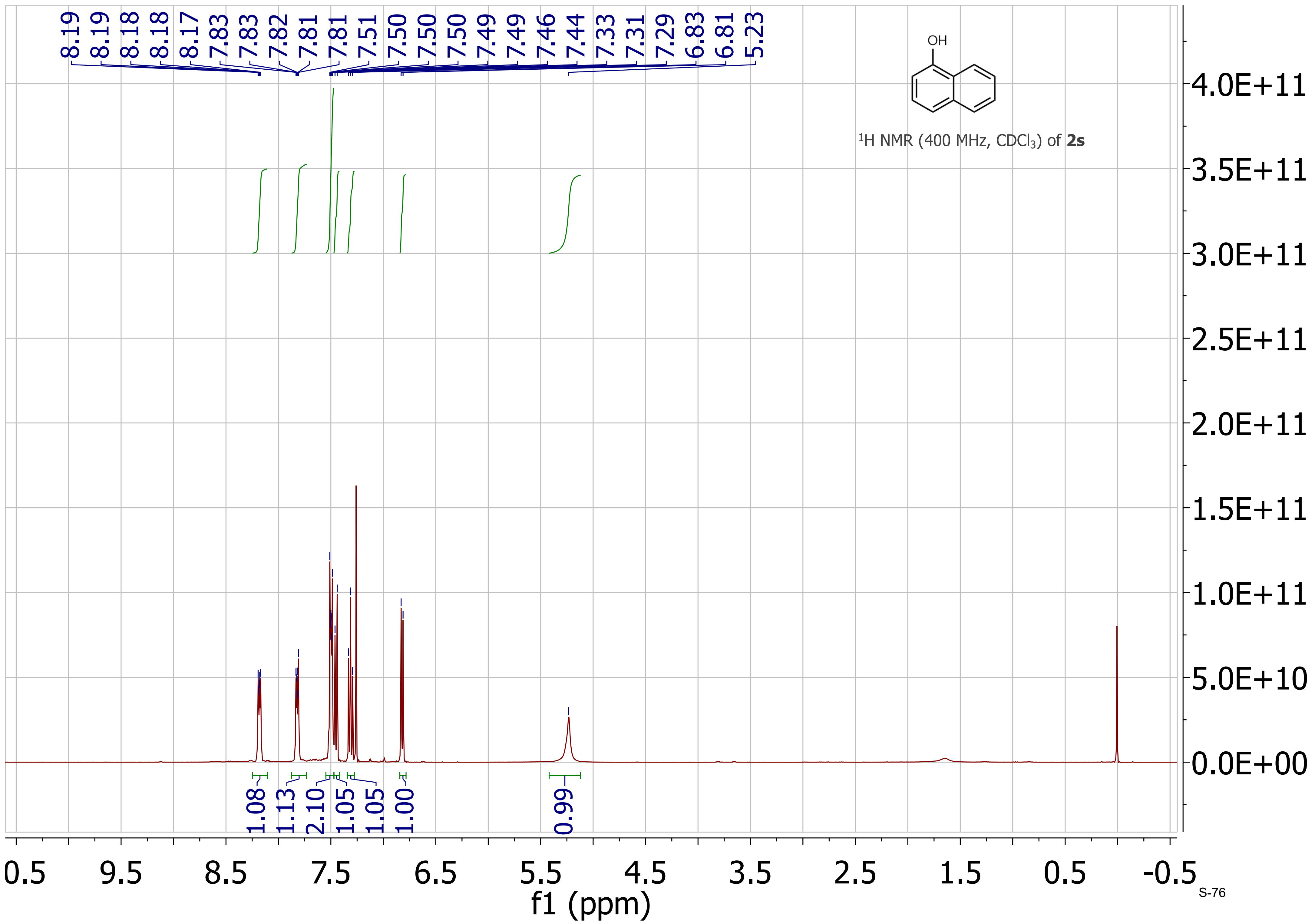

8.19
8.19
8.18
8.18
8.17
7.83
7.83
7.82
7.81
7.81
7.51
7.50
7.50
7.50
7.49
7.49
7.46
7.44
7.33
7.31
7.29
6.83
6.81
5.23
OH
¹H NMR (400 MHz, CDCl3) of 2s
1.08
1.13
2.10
1.05
1.05
1.00
0.99
4.0E+11
3.5E+11
3.0E+11
2.5E+11
2.0E+11
1.5E+11
1.0E+11
5.0E+10
0.0E+00
0.5
9.5
8.5
7.5
6.5
5.5
4.5
3.5
2.5
1.5
0.5
-0.5
f1 (ppm)

Spectrum Plot Report

Name	RDQ3-142a	Rack Pos.		Instrument	Instrument 1	Operator	
Inj. Vol. (ul)	0.5	Plate Pos.		IRM Status	Some ions missed		
Data File	RDQ3-142a.d	Method (Acq)	DAG 200-2000 Method.m	Comment		Acq. Time (Local)	8/14/2025 1:03:47 PM (UTC-04:00)

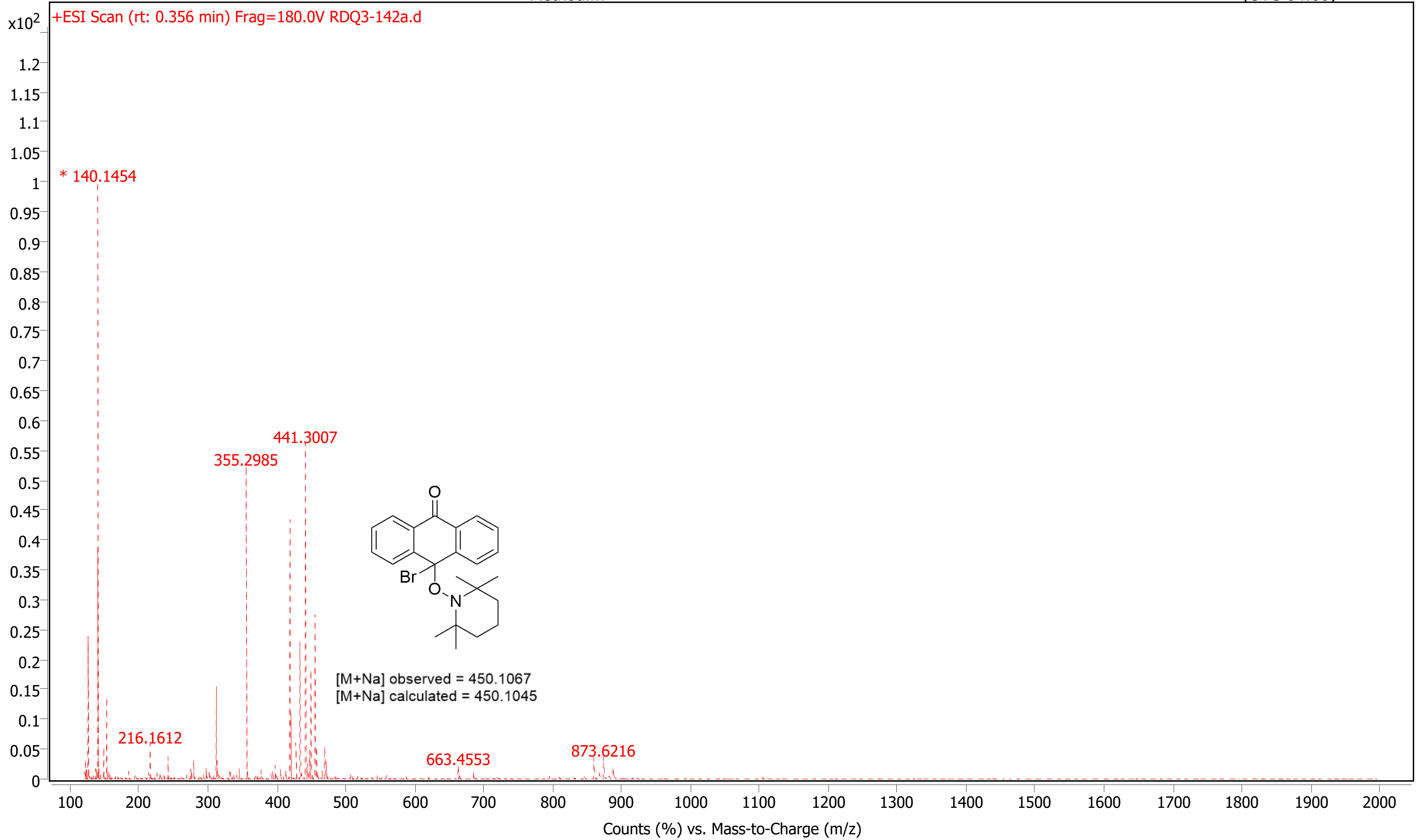

Generated at 2:52 PM on 8/14/2025

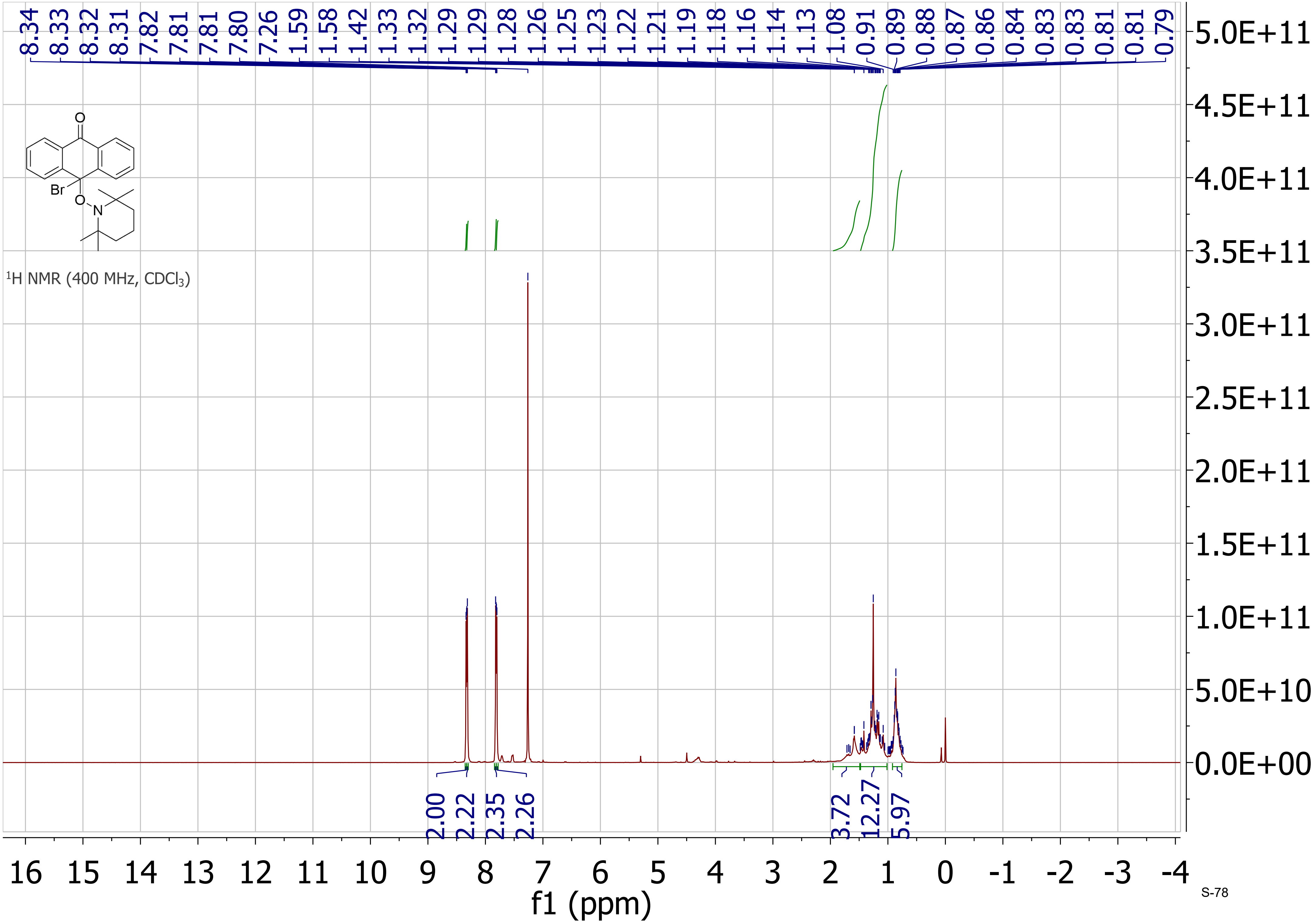

8.34
8.33
8.32
8.31
7.82
7.81
7.81
7.80
7.26
1.59
1.58
1.42
1.33
1.32
1.29
1.29
1.28
1.26
1.25
1.23
1.22
1.21
1.19
1.18
1.16
1.14
1.13
1.08
0.91
0.89
0.88
0.87
0.86
0.84
0.83
0.83
0.81
0.81
0.79
O
Br
O
N
¹H NMR (400 MHz, CDCl₃)
2.00
2.22
2.35
2.26
3.72
12.27
5.97
5.0E+11
4.5E+11
4.0E+11
3.5E+11
3.0E+11
2.5E+11
2.0E+11
1.5E+11
1.0E+11
5.0E+10
0.0E+00
16 15 14 13 12 11 10 9 8 7 6 5 4 3 2 1 0 -1 -2 -3 -4
f1 (ppm)

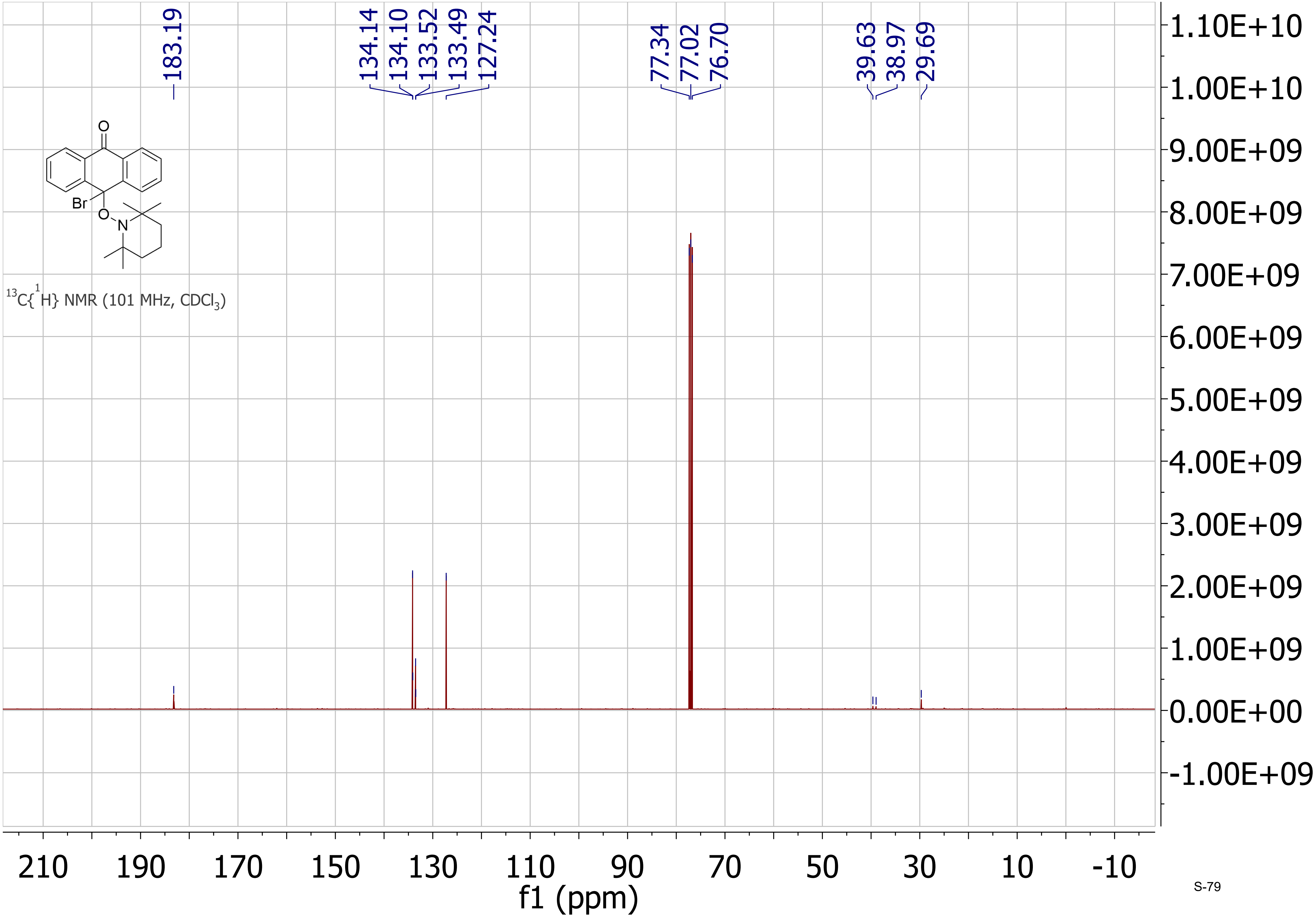

183.19
134.14
134.10
133.52
133.49
127.24
77.34
77.02
76.70
39.63
38.97
29.69
O
Br
O
N
13C{1H} NMR (101 MHz, CDCl3)
1.10E+10
1.00E+10
9.00E+09
8.00E+09
7.00E+09
6.00E+09
5.00E+09
4.00E+09
3.00E+09
2.00E+09
1.00E+09
0.00E+00
-1.00E+09
210
190
170
150
130
110
90
70
50
30
10
-10
f1 (ppm)
